# Towards conceptualizing patients as partners in health systems: a systematic review and descriptive synthesis

**DOI:** 10.1186/s12961-022-00954-8

**Published:** 2023-01-25

**Authors:** Meredith Vanstone, Carolyn Canfield, Cara Evans, Myles Leslie, Mary Anne Levasseur, Maggie MacNeil, Manisha Pahwa, Janelle Panday, Paula Rowland, Shipra Taneja, Laura Tripp, Jeonghwa You, Julia Abelson

**Affiliations:** 1 Department of Family Medicine, McMaster University, 1280 Main St W, Hamilton, ON L8S 4K1 Canada; 2 Centre for Health Economics and Policy Analysis (CHEPA), McMaster University, 1280 Main St W, Hamilton, ON L8S 4K1 Canada; 3 Patient Advisors Network (PAN), Toronto, ON Canada; 4 Department of Family Practice, University of British Columbia, 5950 University Boulevard, Vancouver, BC V6T 1Z3 Canada; 5 Health Policy PhD Program, McMaster University, 1280 Main St W, Hamilton, ON L8S 4K1 Canada; 6 School of Public Policy, University of Calgary, 906 8Th Avenue S.W., Calgary, AB T2P1H9 Canada; 7 Occupational Cancer Research Centre, Cancer Care Ontario, Ontario Health, 505 University Avenue, Toronto, ON Canada; 8 Wilson Centre and Department of Occupational Science and Occupational Therapy, Temerty Faculty of Medicine, University of Toronto, Canada 1 King’s College Circle, Toronto, ON M5S 1A8 Canada; 9 Department of Health Research Methods, Evidence and Impact, McMaster University, 1280 Main St W, Hamilton, ON L8S 4K1 Canada

**Keywords:** Patient engagement, Systematic review, Health systems

## Abstract

**Background:**

With the sharp increase in the involvement of patients (including family and informal caregivers) as active participants, collaborators, advisors and decision-makers in health systems, a new role has emerged: the patient partner. The role of patient partner differs from other forms of patient engagement in its longitudinal and bidirectional nature. This systematic review describes extant work on how patient partners are conceptualized and engaged in health systems. In doing so, it furthers the understanding of the role and activities of patient partners, and best practices for future patient partnership activities.

**Methods:**

A systematic review was conducted of peer-reviewed literature published in English or French that describes patient partner roles between 2000 and 2021 in any country or sector of the health system. We used a broad search strategy to capture descriptions of longitudinal patient engagement that may not have used words such as “partner” or “advisor”.

**Results:**

A total of 506 eligible papers were identified, representing patient partnership activities in mostly high-income countries. These studies overwhelmingly described patient partnership in health research. We identified clusters of literature about patient partnership in cancer and mental health. The literature is saturated with single-site descriptive studies of patient partnership on individual projects or initiatives. There is a lack of work synthesizing impacts, facilitating factors and outcomes of patient partnership in healthcare.

**Conclusions:**

There is not yet a consolidated understanding of the role, activities or impacts of patient partners. Advancement of the literature has been stymied by a lack of consistently used terminology. The literature is ready to move beyond single-site descriptions, and synthesis of existing pockets of high-quality theoretical work will be essential to this evolution.

**Supplementary Information:**

The online version contains supplementary material available at 10.1186/s12961-022-00954-8.

## Background

The last two decades have seen a sharp increase in the involvement of patients, family and informal caregivers as active participants, collaborators, advisors and decision-makers in health systems, catalysed by large initiatives in several countries [1–4]. Family and informal caregivers are those who have significant personal relationships and provide a broad range of assistance for those with health concerns [5].

While the domains of healthcare and quality improvement have long been recognized areas for active involvement, patients, family and caregivers are now formally contributing to all facets of the health system including the training of health professionals, health research, policy-making, governance and regulation [6, 7]. As the patient engagement movement has evolved over time, emphasis has shifted to include more collaborative partnership models in addition to traditional consultation roles [8, 9]. These partnership roles aim to place patients on an equal footing with healthcare professionals, researchers, managers and/or policy-makers [10–12]. These roles are given various labels, such as *patient partner* or *patient advisor*. In this paper, we use “patient” as an overarching term which includes those with personal health concerns and their friends, family and other informal caregivers who together engage with health and organizational systems [13]. By patient partner, we mean people with lived experience who have longitudinal, bidirectional involvement with health organizations for the purpose of system change.

While there is evidence that this type of collaborative partnership activity with patients is growing, [14, 15] the nature and impact of these roles is not well understood. Although there are many papers describing patient partnership, few move beyond single-site descriptions of involvement [14, 16–20]. As a result, the literature on the activities of patient partners is voluminous but there has been little synthesis to describe the role of patient partners across multiple health system domains. The small body of literature that examines the role, impact or outcomes of patient partners and advisors indicates that a partnership approach, when contrasted with less collaborative roles, is well positioned to influence care process and outcomes [8, 21, 22]. It also suggests that patient partners generally view their roles positively and experience increased self-esteem and feelings of empowerment and independence, and some continue to seek greater and more meaningful involvement [8, 19].

A fuller understanding of the barriers, facilitating factors, benefits and drawbacks of patient partnership is obstructed by the lack of consistent understanding of the various ways that the role of the patient partner can be operationalized. We have no consolidated understanding of how the patient partner role has been defined and how these individuals have been engaged, in what roles, to participate in which activities, or how patients have experienced these roles. To help fill this gap in knowledge, we conducted a systematic review to answer the research question, “*how are patients conceptualized and engaged as partners in health system decision-making*?” This is a purposefully wide question, designed to elicit a broad swathe of literature so that we may offer a high-level description of what is known about both the patient partner role and patient partnership activities within the health sector. To this end, the objective of this review is to characterize the literature on patient partnership, define how this emerging group of people is conceptualized and describe the way they are engaged by delineating activities of this group.

## Methods

We performed a systematic review of literature on patient partner involvement in health systems. This review was registered with PROSPERO (CRD42020171742), and the protocol is available through that site. The current manuscript answers the first research question listed in the registered protocol.

### Partnership practices

We engaged in patient partnership in the current study, incorporating two research team members with lived experience of the healthcare system as caregivers and more saliently, a long history of working as patient partners (MAL, CC). MAL and CC are cofounders of the Patient Advisors Network, a national peer-led patient partner network [23, 24]. The aim of involving patient partners was to ensure that our research was relevant and resonant with this stakeholder community. As full research team members, MAL and CC were integral contributors to the conception, design and execution of the current study. They were instrumental in designing the research question, defining the eligibility criteria and boundaries of the search, and in helping to interpret the data and frame the manuscript. CC participated in the origination of the idea and application for funding, and both MAL and CC attended research team meetings and provided regular written and oral feedback as the project developed. MAL and CC continue to participate in similar ways (strategic planning, operational and interpretive guidance) on the broader programme of research on patient partnership which this study involves, including a nationwide survey of patient partners and a qualitative study of patient partners and organizational staff who work with patient partners [23, 24].

### Literature search

The search strategy (Additional file 1: Appendix S1) was developed by a research librarian in collaboration with multiple topic experts. On 16 January 2020 we searched the following databases: MEDLINE, HealthSTAR, Cumulative Index to Nursing and Allied Health Literature (CINAHL) and Social Sciences Citation Index. The search strategy comprised both controlled vocabulary (e.g. Medical Subject Headings) and keywords. The search was updated through 31 May 2021.

Eligible articles were peer-reviewed and published in English or French between 1 January 2000 and 31 May 2021. We did not apply any geographical or methodological limits and included both theoretical and empirical articles drawing on either primary or secondary data. We did not restrict eligibility based on particular diseases or demographics, instead including articles which described partnership with people with lived experience of illness or caregiving who had ongoing involvement with a health organization for the purpose of system change. Eligibility criteria are described in Table 1.

**Table 1:** Eligibility criteria

General category	Specific category for this project
Disease	No limits
Subcategory of disease population	N/A
Relevant perspectives	Any
Age	N/A
Gender	N/A
Country	No limits
Language	English or French
Publication status	Published (e.g. no theses). Peer-reviewed
Dates	2000–2021
Role	Patient partner or patient advisor: people with lived experience (as a patient, caregiver, parent). Partners/advisors have ongoing engagement with the system, which means multiple points of involvement over time. They are broadly engaged in making system change within the health system or an organization, which means they are involved in changing healthcare delivery for individuals other than themselves
Domain	Health system, engagement in health research, health technology assessment, policy-making, drug and device decisions and implementation, etc. Must be about HEALTH and must occur at a system or organization level. Other than that, no restrictions

Given that the nature of our search was to understand the broad variety of ways in which the literature describes the role of patient partners, we required an operationalized definition of patient partner that would be inclusive, but still useful in differentiating literature on patient partnership from the broader field of patient engagement and involvement. However, we did not wish to define a priori what a patient partner was, given that defining this emerging category of people was one of the goals of the review. Instead, we created a negative definition, choosing to exclude articles that did not include people with lived experience of the healthcare system pertinent to the issues on which they were engaged. For example, we excluded papers describing involvement of members of the lay public unless that involvement was relevant to experiences with the healthcare system that were ubiquitous in that population, such as access to primary care or participation in population-based screening programmes. To operationalize our “ongoing involvement” criterion, we excluded articles where engagement with the organization was not ongoing and dialogic (e.g. one-time engagements, responding to surveys). We operationalized “involvement for the purpose of system change” to exclude articles which only described patient involvement in their own care (e.g. participation in shared decision-making about individual treatment plans).

The adequacy of the search strategy was confirmed by asking topic experts to provide a list of five papers they would expect us to find. These topic experts are academics, organizational leaders and patient partners with expertise in different (and often multiple) domains of the health system. They were members of the authorship team and the advisory board for the broader study. We then identified whether those papers were retrieved and, if not, examined the search criteria and adjusted accordingly.

### Article selection

EndNote software was used to manage the article screening and selection process. Article screening and selection was performed independently and in duplicate by MV, JP, MP, LT, JA, ST, CE, JY and MN. Each reviewer screened a selection of references, reviewing full-text articles when the title and abstract did not provide enough information to make a decision about eligibility. A final review was conducted by topic experts JA and LT to ensure that the type of patient partnership described in the article met our definition for eligibility.

### Data extraction

Using a structured form (Additional file 2: Appendix S2), we extracted data from each paper relevant to methodology and topic. This included data about the year and country of publication, the number and type of patient partners, and the domain of health system activity. Methodological data included whether the paper was empirical or theoretical, and what type of data and study design was used. We also extracted data about the terminology used for “patient partner”, criteria for that role, and the activities those people engaged in, for what types of organizations, and with what purpose and objectives. For this last category, we extracted verbatim descriptions from each paper. We also noted whether the paper offered a theoretical rationale or comment on the impact of the patient partner role.

Data were extracted by one reviewer (MV, JP, MP, LT, ST, CE, JY, MN). Three reviewers (JP, MV, LT) spot-checked 30% of the extraction, using randomly assigned portions from the data extraction sheet. Where issues were identified, all data from that reviewer, or in that category, were reconfirmed by two additional reviewers from the broader group.

Typically, researchers engaging in reviews of studies using multiple methods must engage in a process of data transformation in order to ensure that different types of data can be integrated and considered holistically. Because we extracted definitions and descriptions of patient partner roles and engagement, all extracted data were in narrative form and did not require transformation to enable integrated analysis.

### Data analysis

We conducted two types of analysis. First, descriptive analyses were conducted to describe the distribution of included papers by time period, geography and participants. Second, inductive analyses following a conventional content analysis technique were conducted on narrative data to synthesize information about the roles and activities of patient partners [25].

The descriptive, inductive approach to analysis of narrative data allowed us to identify common categories of concepts, theories and arguments across the data set. We used a staged coding process similar to that of grounded theory, but adapted to retain a descriptive rather than interpretive or theory-building aim. Initial descriptive coding was conducted to first identify the points of information that were consistently present across the data set, pertaining to the roles, definitions and types of involvement of patient partners. We refined that analysis through subsequent iterations of focused coding. We then engaged in comparative analysis, grouping data by the domain of the health system the patient was involved in, to create “profiles” of engagement in each domain which could then be compared [26]. Multiple analysts participated in coding (MV, JP, JY, MP, CE, MN), with the whole team engaged to provide formative insight and feedback at various points in the analytical process.

### Critical appraisal

This review describes the extant literature on patient partners, offering synthesized information about the roles and activities of patient partners active in various domains of the health system. We did not synthesize data about the outcomes, impacts or prevalence of patient partnership. Because we focused on examining how the roles and activities of patient partners are described, critical appraisal and risk-of-bias assessments were not appropriate and so were not conducted.

## Results

**Fig. 1:**
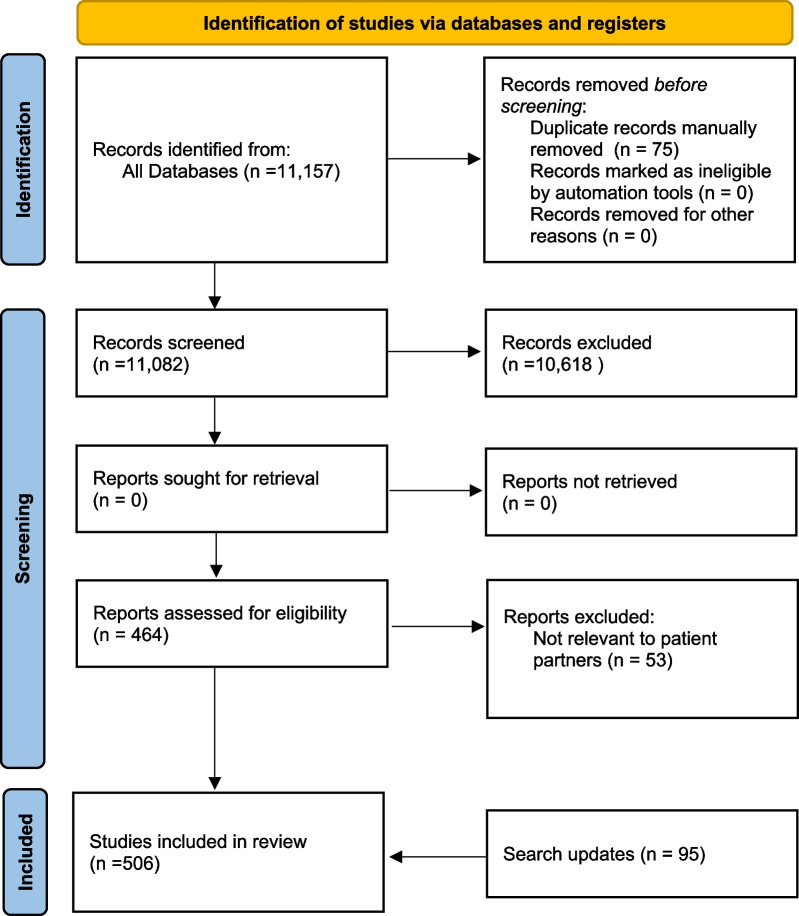
Preferred Reporting Items for Systematic Reviews and Meta-Analyses (PRISMA) [86] diagram showing article selection process. *Databases searched included MEDLINE, HealthSTAR, Cumulative Index to Nursing and Allied Health Literature (CINAHL), Social Sciences Citation Index

The initial search returned 11,157 articles for screening, of which 411 were deemed to be eligible (Fig. 1). A search update performed on 31 May 2021 resulted in the inclusion of an additional 95 articles, for a total of 506 included papers. A list of the main features of each included study is included in Additional file 3: Appendix S3. This collection of literature described patient partner involvement in several different health system domains (Table 2), with most (92%) describing activities in the health research and health planning/service design domains.

**Table 2:** Body of evidence by domain of health system

Domain of health system	Number of eligible studies
Education (health professions)	19
Education (patient)	23
Health planning/service design/quality improvement	152
Health policy/governance	56
Health research	314
Health technology assessment	21
Other	15
Total^a^	600

^a^Some studies identified more than one domain

These papers described the experiences of over 6000 patients who acted as partners with health system organizations. Some articles did not provide a specific number of patient partners engaged, in many cases because they were nonempirical or review articles. These patient partners had a wide variety of conditions or experiences which brought them into contact with the healthcare system and provided the lived experience they drew on in their patient partner roles (Table 3). We note clusters of papers describing patient partners with experience of cancer (*n* = 49) and mental health conditions (*n* = 80). A sizeable number of papers (*n* = 164) discussed patient partners without reference to specific diseases or interactions with the healthcare system, including most of the review (secondary empirical) papers. When compared with papers about other types of lived experience, the cluster of mental health papers has a higher proportion published before 2013, and from Australia, and a lower proportion from the United States of America.

**Table 3:** Body of evidence by lived experience with healthcare system

Disease/condition	Number of eligible studies
Aging/older adults	7
Cancer	49
Cardiovascular (advanced heart failure, atherosclerotic cardiovascular disease, cardiovascular disease, congenital heart disease, Fontan circulation, myocardial infarction with nonobstructive coronary arteries, stroke)	11
Chronic conditions (chronic illness, chronic respiratory disease, diabetes)	15
Determinants of health (health inequalities, homelessness, indigenous health policy, patient safety, transitions in care)	6
Disabilities (developmental disabilities, intellectual disabilities, learning disabilities, physical disability, psychiatric disability)	9
Experience of hospital ward or unit (critical illness, emergency care, emergency medicine, hospital intensive care unit, hospital-acquired infection)	7
HIV/AIDS	7
Mental health and addictions (attention-deficit/hyperactivity disorder [ADHD], child loss/bereavement, depression, maternal depression, psychiatric genomics, psychosis, schizophrenia, alcohol and drug addictions)	80
Multiple^a^	52
Nephrology (chronic kidney disease, kidney disease, kidney failure)	11
Neurodegenerative diseases (dementia, Parkinson’s)	11
Neuromuscular disease	14
Not specified	164
Other (asthma, bronchiectasis, COVID-19, cystic fibrosis, drug development, female hypoactive sexual desire disorder, haemophilia, hepatitis C, pressure ulcer, podiatry conditions, liver disease, Lyme disease, deceased organ donations)	15
Paediatric (child health, marginalized children and youth, breastfeeding, paediatric intensive care, preterm birth)	12
Primary care (palliative care, pain management, pregnancy)	11
Rare diseases	5
Rehabilitation	3
Rheumatic diseases	17

^a^Multiple diseases include patient partners with a combination of lived experiences, such as cancer, arthritis, diabetes, stroke, mental health

In terms of their personal characteristics, most papers described patient partners who were adults (*n* = 386), with smaller numbers further identified as older adults (*n* = 21), as children (*n* = 11) or as adolescents (*n* = 16). Information on the gender, race, education or income level of patient partners was not frequently provided. For example, only 46 of 506 papers offered any information on the race or ethnicity of the patient partner group they described. Many of the papers providing information about race or ethnicity had a focus on increasing diversity in patient partnership (e.g. [27]) or described partners who had experiences of social exclusion (e.g. homelessness) or highly stigmatized health conditions (e.g. schizophrenia, HIV/AIDS).

**Fig. 2:**
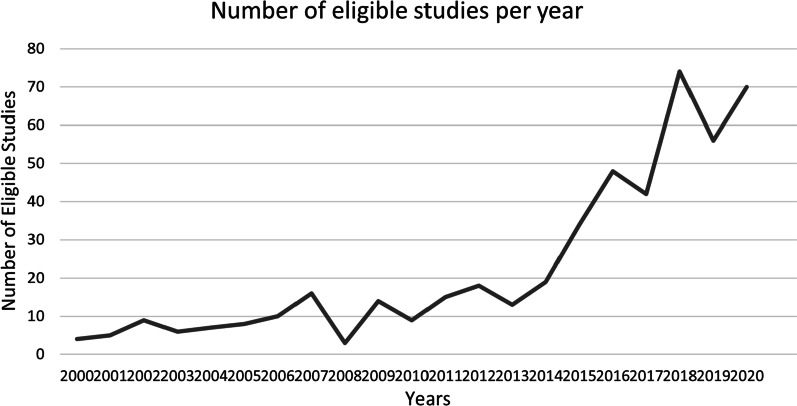
Body of evidence by publication date. Number of eligible studies published each year 2000–2020. 2021 is not portrayed here, since the search was last updated 31 May 2021

Examination of the numbers of articles published per year (Fig. 2) demonstrated a steep increase in eligible papers after 2013. Examining papers published between 2000 and 2012, we can see early dominance in this field from authors working in the Netherlands and the United Kingdom, and patient partners with lived experience of mental health and HIV/AIDS. This early literature was more likely to address patient partners working in the health planning/service delivery and health policy and governance domains. After 2013, the proportion of papers published in the United States of America and papers about patient partners in health research grew rapidly, as did the body of literature about patient partners with lived experience of cancer.

Included articles described research conducted in a variety of countries (Table 4), with most describing activities in single high-income, industrialized countries (e.g. United States, United Kingdom, Canada, Australia). Of our 506 included papers, only five described patient partnership activities in low- or middle-income countries (Indonesia, Malawi, Mali, South Africa—2).

**Table 4:** Body of evidence by country

Country	Number of eligible studies
Australia	26
Canada	95
Denmark	5
France	3
Ireland	6
Netherlands	16
Norway	4
South Africa	2
Sweden	7
Other^a^	9
United Kingdom	131
United States of America	122
Multiple countries	68
Multinational organization	12
Total	506

^a^Belgium, Finland, Germany, Indonesia, Japan, Malawi, Mali, Switzerland, Taiwan

This body of evidence included primary empirical (*n* = 332), secondary empirical (*n* = 63) and nonempirical (*n* = 111) papers (Table 5). The primary empirical papers were mostly qualitative (*n* = 271). Nonempirical papers included theoretical analyses as well as opinion, editorial and commentary pieces.

**Table 5:** Body of evidence by methodological approach

	Number of eligible studies
Primary empirical	332
Qualitative	271
Quantitative	14
Mixed methods	47
Nonempirical	108
Opinion/editorial/commentary	62
Theoretical	49
Secondary empirical (review)	63
Total	506

The 506 included papers offered a wide variety of terms to describe what we are calling patient partners. These terms often paired a word which described the person’s lived experience (e.g. patient, youth, service user, client, parent, community, consumer) with a word that described their organizational role (e.g. partner, advisor, leader, council or network member, expert, advocate, representative). Terms that described the person’s lived experience were broadly distributed, with “patient” the most popular, but service user, parent or family, community and consumer all occurring in at least 30 papers each. Of note, papers in the mental health cluster were unlikely to use “patient”, instead preferring “service user”, “consumer” or other terms specific to lived experience with mental health problems. “Partner” and “advisor” were the most popular terms for organizational role. More recently, terminology with the prefix “co-” is gaining popularity in the health research domain, including co-analyst, co-principal investigator, co-researcher, co-collaborators.

### Qualitative analysis

Our comparative analysis of the ways patient partners were engaged in each health system domain offers some insight on areas of saturation where we have a tremendous volume of information about how patient partners are engaged. It also highlights areas where information is lacking, where descriptions of patient partner involvement are absent or so vague that they are not helpful in developing a clear definition of the role of patient partners. For each health system domain, we describe the activities patient partners participated in, the goals or objectives of those activities and the organizational structures which gave shape to the ways patient partners engaged with others in the organization. This information is summarized in Table 6. Articles which described patient partnership in multiple domains were analysed in each relevant domain. While we coded 15 articles as belonging to “other” health system domains, analysis of these papers yielded findings that were not insightful in this comparative analysis. Throughout this section we have provided exemplar, rather than exhaustive references to papers which illustrate each finding. We chose the exemplar references based on their representativeness of other papers, influence, richness, utility. Where noted, the references may point to studies which were outliers in the data set.

**Table 6:** Summary of qualitative analysis of the activities, goals and organizational structures that characterize patient partnership in each domain of the health system

Domain (number of papers)	Activities	Goals or objectives	Organizational structures
Health research (314)	(1) Activities specific to the conduct of a particular research project (2) Activities related to priority-setting, governance, scoping of larger programmes of research	Contribute to the design and conduct of research responsive to the needs of patients and communities	(1) Research team members, collaborators, co-investigators interacting directly with other researchers (2) Advisory group, steering committee members
Health planning, service design, quality improvement (152)	Participated in activities to define and improve clinical pathways. Often only general description of what these activities were	Develop patient-centred clinical care processes to improve delivery of healthcare, contribute to improvement of organizational initiatives	Patient advisory committees, sometimes called stakeholder or community boards or councils, which typically provide input to a planning or service design process
Health policy and governance (56)	Wide array of activities, including offering opinions and perspectives, tangible assistance operationalizing programmes, co-constructing priorities and policies	Most commonly, improving safety and quality of care, assessing and implementing health services and technologies	Participation in clinical governance and guideline development groups, including autonomous patient-only groups or serving as representatives on governing boards and advisory councils which offer insight and/or oversight to decision-makers
Patient education (23)	Develop education material, study information, mentoring other patient partners, delivering education to patients, sharing journeys and experiences	Most commonly described in papers addressing multiple domains, where goals of patient partnership contributed to broader goals of research project or health service organization	Members of advisory board or council, research partners or collaborators
Health professional education (19)	Development, delivery, evaluation of education programming through creation of content and resources. Delivery of content and teaching to trainees	Ensure that learning content reflected patient experiences and preferences	Few structures described beyond participation in consultation meetings or acting in teaching roles as mentor or “co-tutor”
Health technology assessment (HTA) (21)	Few specific examples, general descriptions of “provide advice”, submit evidence, review protocols. Activities typically occurred in discrete phases of the HTA process	Support funding decisions about health technologies	Representatives of patient organizations or groups structured by organization to elicit patient insight, seldom interacting with organizational staff. Less commonly, members of advisory committees alongside other experts and organizational staff

#### Health research (*n *= 314)

The health research domain contributed the largest number of papers, and these papers provided very specific information about the types of activities patient partners participated in. Patient partners engaged in two types of research activities: activities specific to the design and conduct of an individual project (e.g. [28, 29]) and activities related to priority-setting, scoping and governance of a programme of research (e.g. [30, 31]). Concerning project-specific activities, patient partners were involved in activities relevant to their particular skills and interests. The lists of activities included nearly every activity required of a research project: study design, recruitment of participants, design of data collection materials, data collection, data analysis and knowledge translation. A small number of papers mentioned patient partners simultaneously engaging as partners and research participants [31–33], while others explicitly mentioned participation in research as an activity which did not constitute patient partnership [34–36]. In most studies, patient partners participated as members of the research team, most commonly named “co-researchers”, and interacted directly with other researchers, research participants and knowledge users. Many papers also mentioned patient partner participation in advisory groups or steering committees available for consultation by researchers who desired patient oversight on priority-setting and study design activities not tied to a specific project [37, 38].

#### Health planning, service design, quality improvement (*n* = 152)

In this domain, patient partners engaged in a variety of activities in service of developing patient-centred clinical care processes to improve the delivery of healthcare. Other studies described organizational goals such as improving collaboration for better quality improvement initiatives [39–43]. In pursuit of these goals, patient partners engaged in activities most commonly related to defining and improving clinical pathways, such as designing a new intake process for patients [44] or creating a diabetes registry and surveillance system [45]. Many studies provided only general descriptions of patient activities as related to ensuring patient centredness through feedback, mentoring or support [42, 46–48]. These activities were organized most commonly through patient advisory committees, although the terminology used for these groups of patients who reported to leadership varied and included stakeholder or community boards or councils. Many articles in this group made no reference to organizational structure. [43, 49–51].

#### Health policy and governance (*n* = 56)

Patient partner activities within this domain were typically in service of broader organizational goals and policies and took place within specific types of organizational structures, often contributing to multiple domains. Studies describing patient partnership activities in health policy and governance delineated these activities as spanning a wide spectrum with different degrees of engagement, from providing opinions, perspectives, experiences and insights [52–59], to tangible assistance in operationalizing programmes [56, 60], to co-constructing priorities, programmes and policies [58, 61–63]. Patients were involved in high-level activities (e.g. oversight and strategic planning) as well as smaller, discrete activities (e.g. producing patient materials) [58, 61, 64]. Patient partners participated in these activities in service of a number of different goals, most commonly those related to improving the safety and quality of care, assessing and implementing health services and technologies, and delivering services. Most commonly, patient partners were involved in offering guidance to policies aimed at reshaping the delivery of healthcare services, often via clinical governance policies and guideline development [65, 66]. Patient partners interacted through a variety of organizational structures. Some of these were autonomous entities composed only of patients, such as patient associations or networks. In other instances, patient partners served as representatives on groups such as governing boards or advisory councils which offered insight and oversight to decision-makers [64, 67]. There was limited evidence of direct communication between patient partners, policy-makers and managers [68], with communication typically mediated through a staff member of the organization. Where these examples did exist, they often arose from the pre-existence of established interest groups [68].

#### Health professional education (*n* = 19) and patient education (*n* = 23)

Patient partners in health professional education participated in the development [69–71], delivery [69, 71–73] and evaluation of educational programming [69, 71]. They helped develop curricula and resources through sharing of narratives and perspectives to inform the development of materials [70] and created curricular content [71]. Patient partners also delivered content and occupied teaching roles as “mentors” or “co-tutors” [73]. They assessed the impact of educational initiatives on learners and educators [71]. These activities were most frequently structured to ensure that educational programming reflected patient preferences and experiences. Few structures for engagement were described, beyond participation in consultation meetings [70] or teaching roles [73].

The domain of patient education was most often described in articles where multiple domains of patient partnership were involved (e.g. health research, service planning or policy initiatives). Patient education activities included the development of educational material [70, 74] and study information media [75, 76], mentoring or coaching other patient partners [77, 78], delivering education to other patients [79, 80], and sharing their journeys and experiences [81]. Organization-wise, these patient partners were often members of an advisory council or board [77], stakeholders [70], or research partners or collaborators [78]. Engagement levels were not often clearly described, although involvement ranged from giving opinions to being trainers.

#### Health technology assessment (*n* = 21)

Articles describing patient partners engaging with health technology assessment (HTA) organizations provided few specific examples of the activities in which patient partners engaged. Often, activities were described in general ways such as “provide advice” [82] or “guide health decision-making” [83]. The goals of these activities were in service of the broader goal of supporting funding decisions about health technologies, but mainly remained supportive (e.g. submitting evidence, reviewing protocols, co-conducting research) and occurred in discrete phases of the HTA process [83–86]. There were few examples of patient partners participating directly in funding or assessment decisions about specific technologies. When they did participate in decision-making it was typically at the operational stages rather than at the level of overall decision-making about a recommendation for a particular health technology [87]. The phase at which patients were involved in the process influenced the organizational structure of their involvement. Many patient partners were engaged as representatives of patient organizations [87], while others participated in advisory committees or groups structured by the HTA organization to elicit patient insight [86, 88].

## Discussion

Our systematic review of literature on patient partnership revealed a plethora of work in this area. Publication of research, theory and commentary about patient partnership has accelerated since 2013 and continues to grow rapidly, driven mainly by publications from the domain of health research. Different terms, concepts and contexts for longitudinal and continued integration of patient voices has limited the ability of the field to move beyond descriptive studies within individual organizations. One conclusion of this synthesis is that the next step for this field will be to build upon the foundation of existing work in a more systematic way in order to prioritize studies of impacts, outcomes and best practices beyond single organizations.

In this manuscript we have demonstrated domain-specific practices of patient partnership, highlighting different organizational structures at work in particular domains. For example, while patient partners are often integrated alongside decision-makers and other experts in health research projects, the literature in the health policy/governance and HTA domains documented that patient partners in these domains are less likely to interact directly with decision-makers and more likely to be engaged in structures where they interact mainly with other patients. Patient partnership in the education of health professionals is not widely discussed, and is typically limited to instruction and feedback on particular aspects of practice related to their specific lived experiences, rather than engagement in higher-level priority-setting or direction-setting work seen in some health research networks. The differences across domains demonstrate the opportunity to interrogate the taken-for-granted practices in one’s own organization or domain. These gaps represent opportunities for the respective fields to start addressing some of these challenges by, for example, structuring patient partnership to include engagement with decision-makers or to involve patient partners in more strategic planning of health professional education programmes.

It is important to acknowledge that the literature we identified through this systematic review came disproportionately from the domain of health research. We noted that the explosion of patient partnership literature in health research came after 2013, potentially reflecting the funding available through the United States-based Patient-Centered Outcomes Research Institute (PCORI), which has disbursed over US$ 3.6 billion between 2010 and 2020, and mandates partnership with patients in the entire research process [3]. While smaller in scale, other countries have also prioritized the inclusion of patients throughout the research process, likely contributing to the proliferation of publications in this area. [1] The profusion of papers in the health research domain also likely reflects the built-in incentives and infrastructure for peer-reviewed publications in the research domain. It may also mean that we are missing rich lessons from the experiences of patient partners in other health system settings that are more likely to be unpublished or published in the grey literature.

### Areas of sufficiency

We identified several areas where existing literature is robust and in some places duplicative. First, there is no need for more single-site descriptive studies of how patient partners have been engaged in particular health research projects. The recent creation of tools such as a patient and public involvement search strategy may assist in identifying extant literature and reducing duplication in new work [89]. That said, more rigorous, theory-driven case studies could be useful, demonstrating broader concepts such as general ethical or practical challenges in engaging patient partners [90, 91]. There were various constellations of studies about the engagement of patient partners with similar types of lived experience (e.g. cancer, mental health, multiple chronic diseases) which crossed domains of the health system. This focus on studying patient partners with similar types of lived experiences implies the importance of the particularities of that experience, but it also creates duplication in papers which produce similar findings about patient partners with different types of lived experience engaged in similar health system activities. For those wishing to learn about how to better enact patient partnership, it may be more useful to identify studies of patient partners in similar domains, organizational contexts, or pursuing similar goals rather than focusing on the types of lived experiences those patients have.

### Gaps or points of conflict

We noted several gaps in our review of the current literature. For example, there was next to no information available about how patient partnership occurs in low- and middle-income countries. Limiting eligibility to French and English language publications may have contributed to this gap in the review. Second, there was very little information about how patient partners are engaged in most domains, such as exactly what activities they participated in. While the literature on partnership in health research domains provided an overwhelming level of detail about the particular activities of patient partners, the majority of papers in other domains used opaque language such as “advise”, “guide”, or “provide perspective” without details about how that was accomplished, or how the information was solicited, integrated and used.

There were also very few studies describing the demographic features and experiences of patient partners, beyond describing their lived experience of the healthcare system. Among the papers which did provide some demographic information about their patient partners, few reflected that information against the larger population of patients those partners represented. This lack of attention to the social identities of patient partners presents a challenge to thinking through issues of representation, marginalization, and privilege. Opportunities for addressing this particular issue include gathering information about social identities of patient partners, and critically comparing this information against relevant populations to identify which perspectives may not be fulsomely included. This could be integrated as part of the “people involved” section of the long-form GRIPP2 tool for reporting patient involvement [92]. Beyond this descriptive approach, it will be critical to interrogate and remediate the barriers to participation faced by potential patient partners in order to design activities which are inclusive of a wider variety of individuals [8, 93].

### Implications for future research

An examination of the existing literature points to some priorities for future research. First, and most urgent, is the need for a definition or conceptualization of patient partner. The field of patient engagement has grown rapidly, and there is little conceptual distinction between patients who are engaged in a time-limited, unidirectional way, and those who have engaged in a more integrated, longitudinal way to influence decision-making processes. Given the acknowledgement that these longitudinal partnerships result in the development of expertise within the patient partner and are more likely to impact decision-making in the organization, it is useful to name and examine this form of patient engagement as distinct from other types. Differentiating between “transactional” and “relational” forms of patient partnership may encourage engagement where the purpose is cumulative and interactive learning that develops commitment and capacity among all. This may assist in the development of research about the impact patient partners are having, answering many questions about what types of impact patient partners have, to whom this impact is visible and what effect this impact has [94, 95]. We found very little information about the identities and perspectives of patient partners beyond their lived experience with the healthcare system, making it difficult to determine what perspectives these individuals are bringing to health system decision-making.

### Implications for policy and practice

In some domains, there is much learning possible from existing publications on ways that other groups have engaged patient partners. In other domains, there is very little literature about how patient partners have been engaged, and there is not yet enough information to learn from what others have done. This may reflect a lack of information in the peer-reviewed literature, as we did not search the grey literature for this information.

### Strengths and limitations

This is the first attempt we can identify to synthesize evidence on the role and activities of patient partners, establishing that this is a distinct type of role that exists across multiple domains, countries and types of lived experience. The comprehensiveness of this review allowed for comparative analysis of the diverse literature which exists on this new role. The comprehensiveness is also a limitation. Because there is no clear conceptualization of patient partnership, we needed to use a very broad search strategy and create our own definition to select articles, with the risk that we would find only what we set out to look for. The very large size of the data set and our desire to offer a summary of the whole collection of papers limited our ability to provide an interpretive synthesis of the richest papers. It also meant that in our analysis, not all perspectives could be covered. For instance, we did not analyse these papers with the aim of commenting on who defines the roles of patient partners in different health domains, or how situational expectations may change these roles across domain or context. Finally, we searched only the peer-reviewed literature. Rich data on this topic likely exist in the grey literature, as many organizations do not prioritize peer-reviewed publication and may instead share information via websites and reports. Searching the grey literature in this area will be an important next step for future research.

## Conclusion

Patient partnership is a unique and emerging role within the healthcare system, allowing organizations to benefit from prolonged engagement with people who have lived experience of the healthcare system. The 506 papers identified in this systematic review indicate the wide adoption of longitudinal forms of patient engagement, particularly within the domain of health research. This literature is growing exponentially, but mainly comes from a small group of high-income industrialized countries. In order to further generative engagement with patient partners, it will be important to learn from what already exists, and to build a theoretically informed foundation from which to examine the impacts of patient partners and create best practices for ethical, productive and mutually beneficial engagement.

## Supplementary Information

**Additional file 1**. **Appendix 1:** Literature search methods.

**Additional file 2**. **Appendix 2:** Data extraction form.

**Additional file 3**. **Appendix 3:** Summary of included studies.

## Data Availability

All data are publicly available. A full list of included papers is available in Additional file 3: Appendix S3.
