# Supplementary material for: Towards conceptualizing patients as partners in health systems: a systematic review and descriptive synthesis
Source: Health Res Policy Syst. 2023 Jan 25;21:12. doi: 10.1186/s12961-022-00954-8 (PMC9876419; doi:10.1186/s12961-022-00954-8)
Supplement: Supplementary file 1 — Additional file 1. Appendix 1: Literature search methods. [file 12961_2022_954_MOESM1_ESM.docx]

## **Additional file 1: Appendix S1 - Literature Search Methods**

A search for published literature was performed by a medical librarian on 16 January, 2020 using the following databases: MEDLINE (via Ovid), HealthStar (via Ovid), Cumulative Index to Nursing and Allied health Literature (CINAHL, via EBSCOhost), and Social Science Citation Index (SSCI, via Web of Science). The search strategy comprised both controlled vocabulary (e.g., Medical Subject Headings), and keywords. See Appendix/Supplemental material for literature search strategies, including all search terms.

## **Results (can also inform PRISMA flow reporting):**

Records identified through database searching (n = 27,434)

Records after duplicate records removed automatically and manually (n = 11,082)

**Additional file 1**

## **Literature Search Overview**

Date of Search: January 16, 2020

Databases: MEDLINE (1946-16-January 16, 2020) via Ovid

HealthStar (1966 to October 2019) via Ovid

CINAHL (1981 – January 16, 2020) via EBSCOhost

SSCI (1976 – January 16, 2020) via Web of Science

Limits: English and French language ; peer-reviewed publications excluding conference abstracts

## **Literature Search Strategies**

##

**Databases:** OVID Medline Epub Ahead of Print, In-Process & Other Non-Indexed Citations, Ovid MEDLINE(R) Daily and Ovid MEDLINE(R) 1946 to Present; Ovid Healthstar 1966 to October 2019

| # | Searches | Results |
| --- | --- | --- |
| 1 | exp *Consumer Participation/ | 49921 |
| 2 | *Patient Advocacy/ | 27478 |
| 3 | ((patient* adj3 partnership*) or "patient$1 partner$1" or "patient$1-as-partner$1" or "patient-as-a-partner$1").ti,ab,kf. | 5911 |
| 4 | (patient* adj2 (advisor* or advocate* or council$1 or leader* or voice*)).ti,ab,kf. | 9280 |
| 5 | (patient* adj2 (involvement or engagement or participation)).ti. | 5321 |
| 6 | (patient* adj2 (educator* or teacher* or co-teacher* or instructor* or co-instructor* or mentor$1 or facilitator* or co-facilitator* or co-design* or co-investigator*)).ti,ab,kf. | 2013 |
| 7 | ((patient* adj expert$1) or (expert adj patient*)).ti,ab,kf. | 959 |
| 8 | ((family or caregiver* or care-giver* or carer$1) adj2 (advisor* or advocate* or council$1 or leader* or voice* or partnership*)).ti,ab,kf. | 2411 |
| 9 | ((family or caregiver* or care-giver* or carer$1) adj2 (educator* or teacher* or co-teacher* or instructor* or co-instructor* or mentor$1 or facilitator* or co-facilitator*)).ti,ab,kf. | 2179 |
| 10 | or/1-9 | 99429 |
| 11 | *Health Services Research/ | 32670 |
| 12 | *Biomedical Research/ | 111812 |
| 13 | *Research Design/ | 75948 |
| 14 | ((health or healthcare or medical or clinical or nursing or empirical or qualitative or patient-oriented or effectiveness or patient-centred or protocol*) adj2 research).ti,ab,kf. | 332920 |
| 15 | *Education, Professional/ | 4003 |
| 16 | *Education, Medical/ | 81373 |
| 17 | *Education, Nursing/ | 46166 |
| 18 | *Health Occupations/ed | 5999 |
| 19 | *Health Personnel/ed | 10231 |
| 20 | (education adj2 (health professional* or health care professional* or healthcare professional* or doctor* or physician* or medical student* or nurse* or nursing or health personnel or healthcare personnel or health care provider* or healthcare provider*)).ti,ab,kf. | 58877 |
| 21 | *Teaching Rounds/ | 1497 |
| 22 | (round$1 adj1 (clinical or morning or attending or teaching or grand)).ti,ab,kf. | 3999 |
| 23 | *"Delivery of Health Care"/ | 119442 |
| 24 | *Technology Assessment, Biomedical/ | 12650 |
| 25 | ((technology adj2 assessment*) or HTA or HTAs).ti,ab,kf. | 16174 |
| 26 | *Health Policy/ | 74088 |
| 27 | (((health or healthcare) adj2 (polic* or reform$1 or transform*)) or (polic* adj1 development)).ti,ab,kf. | 115215 |
| 28 | *Decision Making, Organizational/ | 7934 |
| 29 | (decision-making adj1 organization*).ti,ab,kf. | 305 |
| 30 | *Government/ | 10405 |
| 31 | *Government Agencies/ | 10469 |
| 32 | *Regional Health Planning/ | 6914 |
| 33 | (((government* or ministr* or federal* or provinc* or region* or state$1) adj2 (health or healthcare)) or LHIN or LHINs or health authorit*).ti,ab,kf. | 121377 |
| 34 | *Quality Improvement/ | 24253 |
| 35 | ((quality or process*) adj improvement*).ti,ab,kf. | 70752 |
| 36 | *Patient Safety/ | 19267 |
| 37 | (patient* adj safet*).ti,ab,kf. | 56796 |
| 38 | *Health Planning/ | 25811 |
| 39 | exp *Health Planning Organizations/ | 6070 |
| 40 | ((health or healthcare) adj1 planning).ti,ab,kf. | 9067 |
| 41 | *Program Development/ | 16231 |
| 42 | ((program* or tool$1 or service$1) adj1 (design$1 or development)).ti,ab,kf. | 31946 |
| 43 | *Drug Approval/ | 13819 |
| 44 | ((drug$1 or pharmaceutical*) adj2 (approval* or funding or design* or development*)).ti,ab,kf. | 110328 |
| 45 | *Peer Group/ | 17353 |
| 46 | ((peer adj1 (support or mentor* or group*)) or "peer-to-peer").ti,ab,kf. | 15690 |
| 47 | exp *Policy Making/ | 18774 |
| 48 | (polic* adj1 (making or analys#s or development$1)).ti,ab,kf. | 22243 |
| 49 | (((advisory or steering) adj1 committee*) or task force* or review committee* or (government* adj2 commission*)).ti,ab,kf. | 54266 |
| 50 | exp *Governing Board/ | 8824 |
| 51 | ((governing adj2 (board* or bod*)) or (board* adj2 (director* or governor*)) or hospital* board* or trustee*).ti,ab,kf. | 12387 |
| 52 | *Organizational Objectives/ | 4678 |
| 53 | (organization* adj2 (goal* or objective* or mission* or strategic plan* or business plan*)).ti,ab,kf. | 3135 |
| 54 | or/11-53 | 1509225 |
| 55 | 10 and 54 | 19010 |
| 56 | (congress or clinical conference or news or newspaper article).pt. | 509693 |
| 57 | 55 not 56 | 18659 |
| 58 | limit 57 to english language | 17703 |
| 59 | 57 and french.lg. | 241 |
| 60 | 58 or 59 | 17919 |
| 61 | 60 use ppez | 8815 |
| 61 | 60 use hstr | 9104 |

**Database:** CINAHL via EBSCOhost Research Databases

| # | Query | Results |
| --- | --- | --- |
| S1 | (MM "Consumer Participation") | 10,526 |
| S2 | (MM "Patient Advocacy") | 5,510 |
| S3 | TI ((patient* N3 partnership*) or "patient partner" or "patient partners" or "patient*-as-partner*" or "patient*-as-a-partner") | 532 |
| S4 | AB ((patient* N3 partnership*) or "patient partner" or "patient partners" or "patient*-as-partner*" or "patient*-as-a-partner") | 1,591 |
| S5 | TI (patient* N2 (advisor* or advocate* or council or councils or leader* or voice*)) | 1,199 |
| S6 | AB (patient* N2 (advisor* or advocate* or council or councils or leader* or voice*)) | 3,335 |
| S7 | TI (patient* N2 (involvement or engagement or participation)) | 2,486 |
| S8 | TI (patient* N2 (educator* or teacher* or co-teacher* or instructor* or co-instructor* or mentor or mentors or facilitator* or co-facilitator* or co-design* or co-investigator*)) | 228 |
| S9 | AB (patient* N2 (educator* or teacher* or co-teacher* or instructor* or co-instructor* or mentor or mentors or facilitator* or co-facilitator* or co-design* or co-investigator*)) | 902 |
| S10 | TI (((patient* N1 (expert or experts)) or (expert N1 patient*)) | 202 |
| S11 | AB (((patient* N1 (expert or experts)) or (expert N1 patient*)) | 673 |
| S12 | TI ((family or caregiver* or care-giver* or carer or carers) N2 (advisor* or advocate* or council or councils or leader* or voice* or partnership*)) | 423 |
| S13 | AB ((family or caregiver* or care-giver* or carer or carers) N2 (advisor* or advocate* or council or councils or leader* or voice* or partnership*)) | 1,446 |
| S14 | TI ((family or caregiver* or care-giver* or carer or carers) N2 (educator* or teacher* or co-teacher* or instructor* or co-instructor* or mentor or mentors or facilitator* or co-facilitator*)) | 146 |
| S15 | AB ((family or caregiver* or care-giver* or carer or carers) N2 (educator* or teacher* or co-teacher* or instructor* or co-instructor* or mentor or mentors or facilitator* or co-facilitator*)) | 1,004 |
| S16 | S1 OR S2 OR S3 OR S4 OR S5 OR S6 OR S7 OR S8 OR S9 OR S10 OR S11 OR S12 OR S13 OR S14 OR S15 | 27,100 |
| S17 | (MM "Health Services Research") | 5,192 |
| S18 | (MM "Study Design") | 8,187 |
| S19 | TI ((health or healthcare or medical or clinical or nursing or empirical or qualitative or patient-oriented or effectiveness or patient-centred or protocol*) N2 research) | 25,375 |
| S20 | AB ((health or healthcare or medical or clinical or nursing or empirical or qualitative or patient-oriented or effectiveness or patient-centred or protocol*) N2 research) | 75,663 |
| S21 | (MM "Education, Medical") | 19,537 |
| S22 | (MM "Education, Nursing") | 26,409 |
| S23 | (MM "Health Occupations/ED") | 23 |
| S24 | (MM "Health Personnel/ED") | 3,321 |
| S25 | TI (education N2 (health professional* or health care professional* or healthcare professional* or doctor* or physician* or medical student* or nurse* or nursing or health personnel or healthcare personnel or health care provider* or healthcare provider*)) | 12,366 |
| S26 | AB (education N2 (health professional* or health care professional* or healthcare professional* or doctor* or physician* or medical student* or nurse* or nursing or health personnel or healthcare personnel or health care provider* or healthcare provider*)) | 22,640 |
| S27 | TI ((round or rounds) N1 (clinical or morning or attending or teaching or grand)) | 1,679 |
| S28 | AB ((round or rounds) N1 (clinical or morning or attending or teaching or grand)) | 518 |
| S29 | (MM "Health Care Delivery") | 29,235 |
| S30 | (MM "Device Approval") | 962 |
| S31 | TI ((technology N2 assessment*) or HTA or HTAs) | 1,773 |
| S32 | AB ((technology N2 assessment*) or HTA or HTAs) | 2,545 |
| S33 | (MM "Health Policy") | 24,226 |
| S34 | TI (((health or healthcare) N2 (polic* or reform or reforms or transform*)) or (polic* N1 development)) | 13,546 |
| S35 | AB (((health or healthcare) N2 (polic* or reform or reforms or transform*)) or (polic* N1 development)) | 27,621 |
| S36 | (MM "Decision Making, Organizational") | 1,204 |
| S37 | TI (decision-making N1 organization*) | 22 |
| S38 | AB (decision-making N1 organization*) | 139 |
| S39 | (MM "Government") | 3,102 |
| S40 | (MM "Government Agencies") | 5,160 |
| S41 | (MM "Health Systems Agencies") | 237 |
| S42 | TI (((government* or ministr* or federal* or provinc* or region* or state or states) N2 (health or healthcare)) or LHIN or LHINs or health authorit*) | 4,871 |
| S43 | AB (((government* or ministr* or federal* or provinc* or region* or state or states) N2 (health or healthcare)) or LHIN or LHINs or health authorit*) | 26,056 |
| S44 | (MM "Quality Improvement") | 26,266 |
| S45 | TI ((quality or process*) N1 improvement*) | 7,932 |
| S46 | AB ((quality or process*) N1 improvement*) | 19,030 |
| S47 | (MM "Patient Safety") | 26,616 |
| S48 | TI (patient* N1 safet*) | 9,708 |
| S49 | AB (patient* N1 safet*) | 15,863 |
| S50 | (MM "Health Facility Planning") | 286 |
| S51 | TI ((health or healthcare) N1 planning) | 562 |
| S52 | AB ((health or healthcare) N1 planning) | 2,457 |
| S53 | (MM "Program Development+") | 28,143 |
| S54 | TI ((program* or tool or tools or service or services) N1 (design or designs or development)) | 2,071 |
| S55 | AB ((program* or tool or tools or service or services) N1 (design or designs or development)) | 10,835 |
| S56 | (MM "Drug Approval") | 5,384 |
| S57 | TI ((drug or drugs or pharmaceutical*) N2 (approval* or funding or design* or development*)) | 2,976 |
| S58 | AB ((drug or drugs or pharmaceutical*) N2 (approval* or funding or design* or development*)) | 8,009 |
| S59 | (MM "Support Groups") | 5,037 |
| S60 | TI ((peer N1 (support or mentor* or group*)) or "peer-to-peer") | 1,888 |
| S61 | AB ((peer N1 (support or mentor* or group*)) or "peer-to-peer") | 5,948 |
| S62 | (MM "Policy Making") | 5,186 |
| S63 | TI (polic* N1 (making or analysis or analyses or development or developments)) | 1,309 |
| S64 | AB (polic* N1 (making or analysis or analyses or development or developments)) | 5,952 |
| S65 | TI (((advisory or steering) N1 committee*) or task force* or review committee* or (government* N2 commission*)) | 4,464 |
| S66 | AB (((advisory or steering) N1 committee*) or task force* or review committee* or (government* N2 commission*)) | 9,030 |
| S67 | (MM "Governing Board") | 3,599 |
| S68 | TI ((governing N2 (board* or bod*)) or (board* N2 (director* or governor*)) or hospital* board* or trustee*) | 1,995 |
| S69 | AB ((governing N2 (board* or bod*)) or (board* N2 (director* or governor*)) or hospital* board* or trustee*) | 2,030 |
| S70 | (MM "Organizational Objectives") | 6,481 |
| S71 | TI (organization* N2 (goal* or objective* or mission* or strategic plan* or business plan*)) | 84 |
| S72 | AB (organization* N2 (goal* or objective* or mission* or strategic plan* or business plan*)) | 1,410 |
| S73 | S17 OR S18 OR S19 OR S20 OR S21 OR S22 OR S23 OR S24 OR S25 OR S26 OR S27 OR S28 OR S29 OR S30 OR S31 OR S32 OR S33 OR S34 OR S35 OR S36 OR S37 OR S38 OR S39 OR S40 OR S41 OR S42 OR S43 OR S44 OR S45 OR S46 OR S47 OR S48 OR S49 OR S50 OR S51 OR S52 OR S53 OR S54 OR S55 OR S56 OR S57 OR S58 OR S59 OR S60 OR S61 OR S62 OR S63 OR S64 OR S65 OR S66 OR S67 OR S68 OR S69 OR S70 OR S71 OR S72 | 439,780 |
| S74 | S16 AND S73 | 6,049 |
| S75 | (MH "Congresses and Conferences") | 96,579 |
| S76 | S74 NOT S75 | 5,916 |
| S77 | Narrow by Language: - english | 5,916 |
| S78 | Narrow by Language: - french | 5,916 |
| S79 | S77 or S78 | 5,842 |

**Database:** Social Sciences Citation Index (SSCI) --1976-present via Web of Science Core Collection

| #1 | TS=((patient* NEAR/3 partnership*) or "patient partner" or "patient partners" or "patient*-as-partner*" or "patient*-as-a-partner") | 820 |
| --- | --- | --- |
| 2 | TS=(patient* NEAR/2 (advisor* or advocate* or council or councils or leader* or voice*)) | 2038 |
| 3 | TI=(patient* NEAR/2 (involvement or engagement or participation)) | 1691 |
| 4 | TS=(patient* NEAR/2 (educator* or teacher* or co-teacher* or instructor* or co-instructor* or mentor or mentors or facilitator* or co-facilitator* or co-design* or co-investigator*)) | 697 |
| 5 | TS= ((patient* NEAR/1 (expert or experts)) or (expert NEAR patient*)) | 3566 |
| 6 | TS=((family or caregiver* or care-giver* or carer or carers) NEAR/2 (advisor* or advocate* or council or councils or leader* or voice* or partnership*)) | 2025 |
| 7 | TS=((family or caregiver* or care-giver* or carer or carers) NEAR/2 (educator* or teacher* or co-teacher* or instructor* or co-instructor* or mentor or mentors or facilitator* or co-facilitator*)) | 1839 |
| 8 | #7 OR #6 OR #5 OR #4 OR #3 OR #2 OR #1 | 12,115 |
| 9 | TS=((health or healthcare or medical or clinical or nursing or empirical or qualitative or patient-oriented or effectiveness or patient-centred or protocol*) NEAR/2 research) | 118,217 |
| 10 | TS=(education NEAR/2 ("health professional*" or "health care professional*" or "healthcare professional*" or doctor* or physician* or "medical student*" or nurse* or nursing or "health personnel" or "healthcare personnel" or "health care provider*" or "healthcare provider*")) | 15,750 |
| 11 | TS=((round or rounds) NEAR (clinical or morning or attending or teaching or grand)) | 829 |
| 12 | TS=("Health Care Delivery" or “Healthcare Delivery” | 5679 |
| 13 | TS=((technology NEAR/2 assessment*) or HTA or HTAs) | 5028 |
| 14 | TS=(((health or healthcare) NEAR/2 (polic* or reform or reforms or transform*)) or (polic* NEAR development)) | 78,119 |
| 15 | TS=(decision-making NEAR organization*) | 3790 |
| 16 | TS=(((government* or ministr* or federal* or provinc* or region* or state or states) NEAR/2 (health or healthcare)) or LHIN or LHINs or "health authorit*") | 26,565 |
| 17 | TS=((quality or process*) NEAR improvement*) | 32,075 |
| 18 | TS=(patient* NEAR safet*) | 16,917 |
| 19 | TS=((health or healthcare) NEAR planning) | 24,755 |
| 20 | TS=((program* or tool or tools or service or services) NEAR (design or designs or development)) | 104,570 |
| 21 | TS=((drug or drugs or pharmaceutical*) NEAR/2 (approval* or funding or design* or development*)) | 4589 |
| 22 | TS=((peer NEAR (support or mentor* or group*)) or "peer-to-peer") | 21,314 |
| 23 | TS=((policy or policies) NEAR (making or analysis or analyses or development or developments)) | 108,385 |
| 24 | TS=(((advisory or steering) NEAR committee*) or "task force*" or "review committee*" or (government* NEAR/2 commission*)) | 9066 |
| 25 | TS=((governing NEAR/2 (board* or bod*)) or (board* NEAR/2 (director* or governor*)) or "hospital* board*" or trustee*) | 5370 |
| 26 | TS=(organization* NEAR/2 (goal* or objective* or mission* or "strategic plan*" or "business plan*")) | 2971 |
| 27 | #26 OR #25 OR #24 OR #23 OR #22 OR #21 OR #20 OR #19 OR #18 OR #17 OR #16 OR #15 OR #14 OR #13 OR #12 OR #11 OR #10 OR #9 | 476,250 |
| 28 | (#27 AND #8) *AND* **LANGUAGE:** (French) | 18 |
| 29 | (#27 AND #8) *AND* **LANGUAGE:** (English) | 3655 |
| 30 | #29 OR #28 | 3673 |
