# Supplementary material for: Towards conceptualizing patients as partners in health systems: a systematic review and descriptive synthesis
Source: Health Res Policy Syst. 2023 Jan 25;21:12. doi: 10.1186/s12961-022-00954-8 (PMC9876419; doi:10.1186/s12961-022-00954-8)
Supplement: Supplementary file 2 — Additional file 2. Appendix 2: Data extraction form. [file 12961_2022_954_MOESM2_ESM.docx]

Additional file 2: Appendix S2 – Data extraction

| Type of Data | Description |
| --- | --- |
| Author | First author’s last name. |
| Year of Publication | Year published. |
| Year(s) of activity | Year(s) of data of patient partner activity and/or when data was collected, if specified. |
| Country | Country or countries data was collected in/organization was situated in. |
| Language | Language of publication. |
| Study Type | Describe whether or not paper used empirical data, was a theoretical analysis etc. |
| Approach (if Non-Empirical) | Definition of Empirical: Studies presenting primary or secondary data.  If it’s non-empirical paper, what is the category of paper (commentary; letter; report). |
| Approach (if Empirical) | If it’s empirical, what is the broad category of methodological approach. |
| Qualitative Methodology | The qualitative methodology used. |
| Quantitative methodology or study design | The quantitative methodology or study design used. |
| Authors Label for “Partner” | The title that the authors used to refer to the patient “partner” . |
| Disease/Condition | If people with lived experience were recruited for experience with a particular disease, condition, or health sector, what was that? |
| Age Category of Patients | If people with lived experience were recruited because they were of a particular age category (e.g. pediatric, older adult), what was that? |
| Role of patient partner in relation to person with disease/condition | Recognizing that not all people with lived experience have the disease or condition of relevance, what was the relationship of the patient partner to the person with lived experience (e.g. self, parent, spouse). |
| Ethnicity (if specified) | The ethnicity of the patient partner, if reported. |
| Domain of health system | Domain the organization is located in, or domain(s) the patient worked in. We used the following pre-determined categories: Health research, health professional education, patient education, health planning/service design/quality improvement, health policy/governance, health technology assessment, other, multiple. |
| Activity | Activities patient partners engaged in (extracted as close to verbatim as possible) |
| Type of Organization (s) | The type of organization(s) with which patient partner/study was involved |
| Purpose/Objectives | The purpose or objective of the paper (extracted as close to verbatim as possible). |
| Number of participating Patient Partners | The number of patient partners in the study. |
| Role Conceptualization | If the role of patient partners was defined, what definition was provided? (extracted as close to verbatim as possible) |
| Use of Theory/Theoretical Rationale | The theory/theoretical rationale used in the paper in relation to patient engagement/involvement. |
| Impact of Patient Partner | The impact of patient partnering on the program/policy/research study and/or the impact of patient partnering on members of the team (including the patient partners themselves) |
| Conceptual Richness | Reviewer’s assessment of whether the paper offers conceptually rich findings about the role of the patient advisor/partner. |
| Comments | Any comments or opinions or notes to remember about this article. |
