# Supplementary material for: Towards conceptualizing patients as partners in health systems: a systematic review and descriptive synthesis
Source: Health Res Policy Syst. 2023 Jan 25;21:12. doi: 10.1186/s12961-022-00954-8 (PMC9876419; doi:10.1186/s12961-022-00954-8)
Supplement: Supplementary file 3 — Additional file 3. Appendix 3: Summary of included studies. [file 12961_2022_954_MOESM3_ESM.docx]

Additional file 3: Appendix S3 – Summary of Included Studies

| **Table 1** Summary of Included Studies | | | | | | | |
| --- | --- | --- | --- | --- | --- | --- | --- |
| **Authors** | **Year** | **Country** | **Study Type** | **Domain of Health System** | **Disease/Condition** | **Age** | **Purpose/Objectives** |
| Abbass-Dick | 2018 | Canada | Empirical | Health Technology Assessment | Breastfeeding | Adult | The objective of this participatory design study was to work in partnership with Indigenous communities to create an eHealth breastfeeding resource for Indigenous families. |
| Abelson | 2016 | Canada | Non-Empirical | Health Technology Assessment | N/A | N/A | The aim of this study was to describe the development and outputs of a comprehensive framework for involving the public and patients in a government agency’s HTA process. |
| Abelson | 2018 | Canada | Non-Empirical | Health Technology Assessment | Oncology | Adult | Commentary on an article discussing patient engagement in HTA. |
| Abelson | 2007 | Canada | Non-Empirical | Health Technology Assessment | N/A | Adult | The purpose of this paper is to offer a framework of public involvement in technology assessment and health policy. |
| Abma | 2005 | Netherlands | Empirical | Health Research | Spinal Cord Injury | Adult | In this article, the author argues that a responsive-constructivist approach to evaluation fits with the aims and features of patient participation. A case study example is used. |
| Abma | 2006 | Netherlands | Empirical | Health Research | Spinal Cord Injury | Adult | The purpose of this article is to investigate the feasibility of a participatory methodology to deal with these communication problems to give patients “a say” in health research. More specifically, this article deals with the participation of patients in the agenda-setting process of health research. |
| Abma | 2009 | Netherlands | Empirical | Health Research | Kidney Disease; Intellectual disability | Adolescent | The purpose of this article is to present two case studies of patient partnership projects; providing a "detailed portrayal of the dynamics within mixed research teams and the presentation of a responsive methodology to handle these dynamics." |
| Abrams | 2021 | Multiple Countries | Review | Multiple | N/A | N/A | The purpose of this review was to 1) describe the ways in which contributors have been involved in realist reviews, with a particular focus on PPI and 2) document how involvement has been reported. |
| Absolom | 2015 | United Kingdom | Empirical | Health Research | Cancer | Adult | The purpose of this article was to describe the development, projects and impacts of a patient Research Advisory Group. |
| Acri | 2014 | United States of America | Empirical | Health Planning/Service Design/QI | Maternal Depression | Adult | The purpose of this paper is to present a feasibility study that describes the development of this intervention in active partnership with family peer advocates, with an emphasis on adapting clinical tools for use by a peer workforce. |
| Adams | 2013 | United States of America | Empirical | Education (Health Professions) | Child Loss/Bereavement | Adult | The purpose of this qualitative research was to study motivations, expectations, challenges, benefits, and meaning making for bereaved parents involved in pediatric palliative care education of health care professionals and to learn about the challenges and possible benefits for the health care professionals. |
| Addario | 2019 | United States of America | Review | Health Research | Cancer | N/A | To show how the integration of the patient and/or patient advocate at all stages of PRO development can help to realize the full potential of PROs. |
| Adler | 2017 | United States of America | Non-Empirical | Health Policy/Governance | N/A | Adult | In this article, I examine how African American soldiers and veterans experienced and shaped federally sponsored health care during and after World War I. |
| Aggarwal | 2011 | United States of America | Empirical | Health Planning/Service Design/QI | N/A | Pediatric | Report of the efforts of the staff of a pediatric hospital in establishing patient-centered care. |
| Aguiar | 2020 | Canada | Empirical | Health Research | Scleroderma | Adult | Using a case study of preferences for stem cell transplant in scleroderma, we report on patient-oriented research approach to developing a discrete choice experiment. |
| AHC Media | 2018 | United States of America | Non-Empirical | Health Planning/Service Design/QI | N/A | Adult | The purpose of this brief report is to provide anecdotal advice on establishing a hospital-based patient and family advisory council. |
| AHC Media | 2019 | United States of America | Non-Empirical | Health Planning/Service Design/QI | N/A | Adult | The purpose of this brief report is provide suggestions on how to maintain a hospital-based patient and family advisory council. |
| Al Hamarneh | 2020 | Canada | Non-Empirical | Health Research | N/A | Adult | Aims to improve the reach of novel/new therapeutic and diagnostic techniques to patients and assists in delivering high quality cost-effective care. |
| Alderson | 2019 | United Kingdom | Empirical | Health Research | Marginalized children and youth | Adolescent | This paper reports on experiences and reflections of a group of children and young people and academic researchers who developed a Patient and Public Involvement (PPI) group that was set up in the context of an ongoing health service intervention trial with looked after children and care leavers (LAC). |
| Alidina | 2021 | United States of America | Empirical | Health Planning/Service Design/QI | N/A | Adult | This study focused on identifying lessons for optimizing patient partnerships, wherein engagement is characterized by shared decision-making and practice improvement codesign. |
| Allarakhia | 2015 | Canada | Non-Empirical | Other | N/A | Adult | This paper argues that patient engagement should occur further upstream during the drug discovery process. It posits that a continuum "lead" patient engagement should be employed that focuses on drug discovery processes ranging from participative, informative to collaborative engement. This article looks at a variety of open innovation models that are currently employed across this engagement spectrum. |
| Amann | 2018 | Switzerland | Empirical | Health Planning/Service Design/QI | Spinal Cord Injury | Adult | The overall objective of this study was thus to explore healthcare professionals’ accounts of and experiences with patient participation, focusing particularly on aspects related to patients’ contributions to the planning and design of healthcare services and products. More specifically, it aimed to determine (1) how healthcare professionals experience patient participation, (2) what factors, in their view, may inhibit or promote it; and (3) through what channels they think it can take place. |
| Ambrosini | 2019 | Multiple Countries | Empirical | Health Research | Neuromuscular Disease | Adult | This paper reports the outcome of a special workshop to investigate the position of the neuromuscular patient community with respect to healthcare and medical research to identify and address gaps and bottlenecks and the specific suggestions derived from the analysis of the first set of topics, related to quality of life. |
| Amirav | 2017 | Canada | Empirical | Health Research | Asthma | Pediatric | This paper reports on parent engagement in an EMR letters study - where the study processes and survey tool were informed by an advisory panel of parents with children with asthma. Although in this article we briefly outline the process of the EMR Letters Study, our main goal is to document 1 parent’s compelling, personal, and initial impression about engaging in this study as a member of the panel. |
| Anderson | 2019a | United Kingdom | Empirical | Education (Health Professions) | Disability (Various) | Multiple | We explored the views of students, patients, and teachers on progressing service users from telling their stories on to a leadership teaching role. We wanted to understand if patients and carers could lead the teaching by taking responsibility for pre-classroom preparation and for the teaching process of shaping student understandings thus moving beyond sharing experiences. |
| Anderson | 2021 | Canada | Empirical | Health Planning/Service Design/QI | N/A | Adult | The objective was to explore approaches and strategies used to engage patients in hospitals recognized for PE capacity including infrastructure and activities. |
| Anderson | 2019b | United States of America | Non-Empirical | Health Research | N/A | Adult | Based on experience conducting >50 patient advisory boards (PABs) during the past several years, the authors provide insights into how to best plan and execute PABs and their value in informing improvement in patient engagement. |
| Anderson | 2018 | United States of America | Non-Empirical | Health Research | N/A | Adult | Based on experience conducting >50 patient advisory boards (PABs) during the past several years, the authors provide insights into how to best plan and execute PABs and their value in informing improvement in patient engagement. |
| Anderson | 2015 | United States of America | Non-Empirical | Health Research | N/A | Adult | We explore the early foundations of patient engagement, where it occurs in the drug-development pipeline, the power of recent policy initiatives, and prospects for success in improving health outcomes. |
| Andersson | 2012 | Sweden | Empirical | Health Planning/Service Design/QI | N/A | Adult | The aim of this study was to investigate managers’ opinions of how to take advantage of patients as resources in quality improvement work in the Swedish healthcare sector. |
| Anderst | 2020 | Multiple Countries | Review | Multiple | N/A | Adult | The aim of this narrative review was to draw together the available research, review findings and relevant governance-related material and to discuss these in light of a case series among research-engaged consumers in order to chart a practical way forward. |
| Anonymous | 2015 | United States of America | Non-Empirical | Health Planning/Service Design/QI | N/A | Adult | Provides a report of the North Carolina Institute of Medicine (NCIOM) Task Force on Patient and Family Engagement |
| Arain | 2013 | United Kingdom | Empirical | Health Research | Cancer | Adult | The objective of this study was to explore different ways of involving consumers in cancer research in one regional network. |
| Arblaster | 2018 | Australia | Empirical | Education (Health Professions) | Mental Health | Adult | This eDelphi study sought to identify consumers’ priorities for curricula, and ways in which they would like to participate in entry-level student education. |
| Archambault | 2018 | Canada | Empirical | Health Research | Emergency Medicine | Adult | To make pragmatic recommendations on best practices for the engagement of patients in emergency medicine (EM) research. |
| Arkind | 2015 | United States of America | Empirical | Health Research | N/A | Adult | We describe developing a Patient Engagement Panel and challenges and lessons learned (e.g., recruitment, funding model, creating value for patient partners, compensation). |
| Armstrong | 2000 | United States of America | Empirical | Multiple | Mental Health | Pediatric | This article describes the natural history of New York’s efforts to create partnerships with families and, from this experience, articulates the stages of parent involvement in policymaking and summarizes lessons learned and recommendations for effective and enduring families as allies policy. |
| Armstrong | 2013 | United Kingdom | Empirical | Health Planning/Service Design/QI | Multiple (Kidney Disease, Aneurysm, Lung Cancer) | Adult | We aimed to characterize patient involvement in three improvement projects and to identify strengths and weaknesses of contrasting approaches. |
| Armstrong | 2017 | United States of America | Empirical | Multiple | N/A | Adult | To investigate the perspectives of potential patient/consumer guideline representatives on topics pertaining to engagement including guideline development group composition and barriers to and facilitators of engagement. |
| Arnstein | 2020 | Multiple Countries | Review | Health Research | N/A | Adult | Our objectives were to: (1) conduct a systematic review of the evidence on patient involvement in results sharing, (2) propose evidence-based recommendations to help maximize benefits and minimize risks of such involvement and (3) conduct this project with patient authors. |
| Attree | 2010 | United Kingdom | Empirical | Health Planning/Service Design/QI | Cancer | Adult | The aim of this study was to explore the influence of the cancer network partnership groups service user involvement activities on cancer care. |
| Baart | 2011 | Netherlands | Empirical | Health Research | Psychiatric genomics | Adult | To analyse the results of an action research process, the aim of which is to involve patients in fundamental psychiatric genomics research, against theoretical backgrounds that formulate a Dialogue Model for patient involvement. |
| Bailey | 2014 | Multiple Countries | Review | Health Research | Disability (Pediatric) | Multiple | This systematic review investigated how disabled children and young people have been involved as research partners; specifically how they have been recruited, the practicalities and challenges of involvement and how these have been overcome, and impacts of involvement for research, and disabled children and young people. |
| Baines | 2018 | United Kingdom | Empirical | Multiple | Multiple | Multiple | To identify and assess the underlying principles of patient and public involvement (PPI) in health and social care services, research, education and regulation across medicine, dentistry and nursing. |
| Baker | 2016 | Canada | Empirical | Health Planning/Service Design/QI | N/A | Adult | In this article, we describe three initiatives to engage patients in quality improvement and health system redesign efforts in three Canadian healthcare contexts and identify key success factors in these efforts. |
| Baldwin | 2018 | Multiple Countries | Review | Health Research | N/A | Senior/Older Adult | This review aimed to investigate the impacts of involving older people in health and social care research on older co-researchers themselves, academic researchers, and research processes and outcomes. A secondary aim was to identify critical success factors and future considerations for involving older people in research. |
| Banerjee | 2020 | International | Empirical | Health Research | Kidney Disease | Adult | The aim of this position paper is to review the need for and challenges of patient involvement; to assess the present situation of patient involvement; and to describe the role of International Society of Nephrology in promoting patient involvement. |
| Banfield | 2018 | Australia | Empirical | Health Research | Mental Health | Adult | In this study, we describe the development and research coproduction activities of an academic lived experience-led mental health research unit in Australia. |
| Banner | 2019 | Canada | Non-Empirical | Health Research | N/A | Adult | In this paper, we will begin to critically examine some of the linkages and tensions that exist between integrated knowledge translation (IKT) and patient-engagement for research and will examine potential opportunities for IKT researchers as they navigate and enact meaningful partnerships with patients and the public. |
| Bar | 2018 | Canada | Empirical | Health Planning/Service Design/QI | N/A | Adult | This paper describes the "Patients as Partners" initiative of the British Columbia Ministry of Health - including how engagement is taking place, types of activities, how teams are being built, how improvement is being monitored etc. |
| Barger | 2019 | United States of America | Empirical | Health research | Cancer | Adult | This manuscript describes the process and resultant impact of engaging a large group of stakeholders in the planning and implementation of the TrACER Study. Our approach and lessons learned contribute to the growing evidence of the value of stakeholder engagement in CER, specifically oncology. |
| Barker | 2020 | United Kingdom | Empirical | Multiple | N/A | Adult | To identify how public contributors established their legitimacy in the functioning of a patient and public involvement programme at a health network. |
| Barnieh | 2015 | Canada | Empirical | Health research | Kidney failure | Adult | Using a recent example of patients on or nearing dialysis, we highlight the key steps to assess research priorities in patients, caregivers, and clinicians: i) formation of a steering committee to guide the overall process; ii) form priority setting partnerships; iii) identify and gather research uncertainties; iv) process and collate submitted research uncertainties and v) final priority setting workshop to determine the top 10 research priorities |
| Bartlett | 2014 | Canada | Non-Empirical | Health Research | N/A | Adult | In this paper, the author reports on the integrating patients and their perspectives into real world research |
| Bate | 2016a | United Kingdom | Non-Empirical | Health Planning/Service Design/QI | N/A | Adult | In this paper, attention is drawn to the burgeoning discipline of the design sciences and experience -based design, in which the traditional view of the user as a passive recipient of a product or service has begun to give way to the new view of users as integral to improve and innovation process. |
| Bate | 2016b | United Kingdom | Non-Empirical | Health Research | Paediatric | Adolescent | In this paper, the author discusses how to involve paediatric patients and their families into research. |
| Batten | 2018 | United Kingdom | Non-Empirical | Health Research | Breast cancer | Adult | In this article, we discuss the process undertaken by ICR-CTSU to optimize patient advocate engagement across a number of our trials requiring the collection of multiple biopsies and how this has ensured successful implementation and delivery of our research. |
| Baumann | 2021 | Multiple Countries | Review | Health Policy/Governance | N/A | N/A | This scoping review focuses on giving an overview of the evidence of PPI methods used in health policy decision making at the macro level. |
| Bayliss | 2016 | United Kingdom | Empirical | Health Research | Chronic Inflammatory Disease | Adult | The aim of this paper is to inform the evidence base on effective ways of involving patients in a qualitative meta-synthesis. This process is evaluated and reflected by patient research partners (PRPs) who provide accounts of their experience |
| Bedlack | 2020 | United States of America | Empirical | Health Research | ALS | Adult | Description of the establishment of a ALS Clinical Research Learning Institute (ALS-CRLI). |
| Bedwell | 2020 | Multiple Countries | Non-Empirical | Health Research | Women's Health/Pregnancy | Adult | To embed PPI into the stillbirth programme of work in sub-Saharan Africa |
| Beier | 2019 | Germany | Non-Empirical | Health research | N/A | Adult | The aim of this debate paper is to critically examine the specific use and ethical role of participatory concepts in the content of HBDR and data-intensive research in medicine and healthcare. |
| Beighton | 2019 | United Kingdom | Empirical | Health Research | Intellectual disabilities | Adult | The main aim of this study was to explore the perspectives and experiences of adults with intellectual disabilities and parent carers of their public and participant involvement in health research study over a 3 year period. |
| Belisle-Pipon | 2018 | Canada | Empirical | Health Research | N/A | Adult | This paper presents the ethical aspects raised by participants in a broader Delphi survey whose primary aim was to examine how PER is being defined and circumscribed, identify its most pressing issues, and encourage the formulation of recommendations to support ECRs conducting PER. |
| Bell | 2019 | Canada | Empirical | Health Research | N/A | Adult | The Foundations in Patient-Oriented Research (‘Foundations’) curriculum is a Canadian example of a co-produced training program. This case study explores the experiences of those who developed and piloted Foundations and their reflections on the project. |
| Benbow | 2012 | United Kingdom | Empirical | Multiple | Old Age Psychiatry | Senior/Older Adult | There are a number of models of patient and carer participation. Their usefulness and applicability to old age psychiatry is considered. Methods: Models of participation are r |
| Benbow | 2011 | United Kingdom | Empirical | Education (Health Professions) | Older age mental health | Senior/Older Adult | We describe in this paper an initiative at the Centre for Ageing and Mental Health, Staffordshire University to design, deliver, and evaluate a module of teaching (In our Shoes) on service users and carer experiences. |
| Bench | 2020 | United Kingdom | Empirical | Multiple | Critical illness | Adult | The aim of the study was to explore former patients’ and family members’ views and experiences of involvement in critical care research and/or quality improvement. |
| Beneciuk | 2020 | United States of America | Empirical | Health Research | Musculoskeletal (MSK) Pain | Adult | The purpose of this manuscript is to describe our collaborative experiences with several MSK pain stakeholders and processes to identify a top priority research topic. |
| Beresford | 2007 | United Kingdom | Non-Empirical | Health Research | Health Inequalities | N/A | Until now, there has been limited consideration of service user involvement in the context of research into health inequalities in comparison to such discussions in health and social care more generally. To address this deficiency, the present author explores and brings together two complex and disputed issues: (1) the involvement of ‘service users’ in research; and (2) the matter of diversity and ‘representativeness’ in service user involvement. |
| Berg | 2015 | Norway | Empirical | Health Planning/Service Design/QI | HIV | Adult | We describe a unique example of a user-driven HIV clinic to build an evidence base about the pragmatics of user involvement in HIV services. |
| Bergerum | 2019 | Multiple Countries | Review | Health Planning/Service Design/QI | N/A | Adult | We aimed to reveal how patient involvement in QI interventions might work in different contexts, to articulate guidance for health‐care organizations on managing active patient involvement in their QI efforts. |
| Bergerum | 2020 | Sweden | Empirical | Health Planning/Service Design/QI | Multiple | Adult | The aim of this study is to examine what might influence interdepartmental hospital process QI teams when involving patients in QI efforts as experienced by QI team members |
| Bergsten | 2014 | Sweden | Empirical | Health Research | Rheumatic diseases | Adult | The project aimed to follow the working process of involving patients in a project group and to describe the research issues that were important from the patient’s point of view |
| Bernstein | 2019 | Canada | Non-Empirical | Health Research | Nephrology | Adult | This article describes patient-centered outcomes research, discusses how patients can be involved throughout the research process, and provides examples for effective partnerships in nephrology nursing research. |
| Bertrand | 2018 | France | Empirical | Health Planning/Service Design/QI | Cystic fibrosis | Multiple | The objective of this article is to report and reflect on patient and parent involvement at the 14 centres engaged in the pilot phase of the PHARE-M program from the perspective of the patients and parents and from the perspective of the professionals on the quality teams |
| Bhati | 2020 | Canada | Empirical | Health Research | N/A | Adult | The present case study examines patients’ experience of engagement in INSPIRE-PHC research studies. |
| Bignami | 2011 | Multiple Countries | Non-Empirical | Multiple | rare diseases | Adult | commentary on roles played by patient organizations relating to advanced therapy medicinal products |
| Birch | 2020 | Multiple Countries | Empirical | Health Research | Ageing; Rheumatoid Arthritis | Adult | The objective of this paper is to describe the development of PPI in EuroTEAM, and the results of two mixed-methods surveys, undertaken towards the end of the project to capture the perspectives of both researchers and patients on the impact of PPI on this research. |
| Bird | 2021 | Canada | Empirical | Health Research | Children with medical complexities | Adult | The aim of this DigiComp Kids Project is to co-design, develop and test a virtual care program that enables children with medical complexities to receive comprehensive, integrated healthcare at home. |
| Bird | 2020 | Multiple Countries | Review | Health Research | Multiple | Adult | Primary aim was to conduct a scoping review of the impact of patient partnership on research outcomes. The secondary aim was to describe barriers and facilitators to realizing effective partnerships. |
| Birkeland | 2020 | Denmark | Empirical | Multiple | Prostate Cancer | Adult | The purpose of this paper is to describe patient and public participation in the development of a national, web-based case vignette survey for studying men’s view on participation in decision-making about PSA screening. |
| Bissel | 2018 | United Kingdom | Empirical | Health Research | Cancer | Adult | In this article, we draw on the work of Jurgen Habermas to explore the ways in which PPI was accomplished in a cancer research setting in England. Drawing on ethnographic data with PPI participants and professional researchers, we describe the ways in which the life-world experiences of PPI participants were shaped by the health research system. |
| Boaz | 2016 | United Kingdom | empirical | Health Planning/Service Design/QI | N/A | adult | he purpose of this paper is to explore the different roles adopted by 63 patients that emerged during and after four participatory quality improvement interventions, and the nature of their impact upon implementation processes and outcomes. |
| Bombard | 2018 | Multiple Countries | Review | Health Planning/Service Design/QI | N/A | Multiple | We conducted a systematic review of international English language literature on strategies for actively engaging patients and families in improving or redesigning health care and the contextual factors influencing the outcomes of these efforts. |
| Bookout | 2016 | United States of America | Empirical | Health Planning/Service Design/QI | Pain management in acute care | Adult | This article describes the process of implementing a PFAC and presents outcomes related to patients’ perception of pain management in the acute care hospital setting. |
| Boote | 2002 | United Kingdom | Review | Health Research | N/A | Adult | The paper explores definitions of ‘the consumer’; why the consumer voice is important in health research; the epistemological challenge that consumer involvement places on health research methodologies; the different levels of consumer involvement in research; and the reservations that clinicians and health researchers have about the concept. |
| Borup | 2016 | Denmark | Empirical | Health Research | N/A | Adult | By shedding light on the views of the different stakeholders regarding the possibilities of patient involvement, the aim of the workshop was to establish a common understanding and provide inspiration for future initiatives within regulatory science, having patient involvement and patient perspectives as key elements. |
| Boudes | 2018 | International | Empirical | Multiple | N/A | Adult | Background: Meaningful patient engagement (PE) in medicines development and during the life cycle of a product requires all stakeholders have a clear understanding of respective expectations. Objective: A qualitative survey was undertaken to understand stakeholder expectations |
| Bourke | 2002 | Australia | Empirical | Health Research | Breast cancer | Adult | This paper provides discussion of some of these issues based on a participatory research project assessing the needs of women with breast cancer in northern Victoria. |
| Boyer | 2018 | United States of America | Empirical | Health Research | Sickle cell disease, cardiovascular disease, and obesity. | Adult | This paper presents our approach to implementing a comprehensive engagement plan for the MS-CDRN and provides a guiding framework for developing a stakeholder-engaged and patient-centered research network |
| Brady | 2018 | United Kingdom | Empirical | Multiple | Experience of alcohol and drug treatment services | Adolescent | Young people with a history of treatment for alcohol and/or drug problems were actively involved in the youth social behaviour and network therapy study. The purpose of this paper is to explore the impact of that involvement on the study and what was learnt about involving young people in drug and alcohol research. |
| Breault | 2018 | Canada | Empirical | Health Research | Depression | Adult | The purpose of this paper is to describe the patient engagement process used to identify depression research priorities and to reflect on the successes of this engagement approach, positive impacts and opportunities for improvement. |
| Brett | 2014 | International | Review | Health Research | N/A | Adult | This paper therefore critically explores the impact of PPI on service users, researchers and communities involved in health and social care research. |
| Bridges | 2007 | United States of America | Non-Empirical | Health Technology Assessment | N/A | Adult | The aim of this study was to develop a working definition of patient-based HTA, to identify the current barriers to adopting a patient-based model, and to formulate a vision of how a patient-based HTA could be used to promote patient empowerment and patient-centered care. |
| Broer | 2014 | Netherlands | Empirical | Health Planning/Service Design/QI | Mental health | Adult | The aim of this article is twofold. First, we study the consequences of a Foucauldian conceptualization of power in analysing practices of client participation. Second, we assign the concept empirical specificity by studying how power is produced. |
| Bronstein | 2016 | United States of America | Non-Empirical | Other | Rare Disease | Adult | Here, we propose a framework of specific steps to rigorously and systematically engage patients at various stages in the development of drugs for rare diseases in order to help provide key information on disease burden, designing trials and evaluating treatment impact |
| Brooke | 2008 | United Kingdom | Empirical | Multiple | N/A | Multiple | The primary focus of this paper is an exploration of the relationship between nursing and public participation as enacted through a nurse-initiated patient council, designed to achieve service user participation in strategic level health care decision-making and planning at the local level within one acute hospital. |
| Brouwers | 2017 | Canada | Empirical | Other | Cancer | Multiple | The objective of this study was to obtain information from cancer patients, cancer survivors, and their families/ caregivers about their awareness of PGs, attitudes towards PGs, interest in participating in PG development, potential barriers and facilitators to participation, and information needs. |
| Brouwers | 2018 | Canada | Empirical | Other | Cancer | Adult | he objective of the pilot study was to collect preliminary data for a determination of the feasibility and efficacy (for example, participant satisfaction) of the two patient engagement models in the pebc setting, using the pebc’s standard engagement practice as a control group for comparison. The results of the 1-year pilot study, conducted from 2016 to 2017, are presented here. |
| Brown | 2016 | United States of America | Non-Empirical | Multiple | Chronic illness | Adolescent | Commentary describing youth engagement initiatives including a hospital-based youth advisory council for adolescent with chronic illness |
| Brys | 2019 | United States of America | Empirical | Health Research | Hospital-acquired infection | Adult | To highlight how stakeholder engagement can be applied to health care epidemiology research, we describe our stakeholder engagement experience and lessons learned. Our aim is to bring attention to the feasibility and benefits of stakeholder engagement and provide guidance to researchers who want to explore stakeholder engagement to increase the relevance and impact of their epidemiologic research. |
| Buck | 2014 | United Kingdom | Empirical | Health Research | N/A | Adult | To describe how plans for PPI were implemented in a cohort of randomized controlled trials funded by the NIHR HTA programme between 2006 and 2010, and to identify the challenges and lessons learnt by research teams |
| Buck | 2004 | United States of America | Empirical | Multiple | Homelessness | Adult | In this descriptive study, we summarize the preliminary findings of a program in which the homeless were involved in the administration of HHH. |
| Burns | 2017 | Multiple Countries | Empirical | Health Research | Weaning from ventilation in ICU | Adult | The present article describes our experience with patient and family engagement in designing and implementing a multicenter, North American weaning trial, the Frequency of Screening and SBT Technique Trial (FAST Trial). |
| Burns | 2020 | Canada | Non-Empirical | Multiple | N/A | Adult | This articles illustrates how patients were meaningfully engaged in a large-scale change initiative. |
| Caldwell | 2009 | United States of America | Empirical | Health Research | Developmental disabilities | Multiple | This study adopted a participatory action research design to explore supports that enhance meaningful participation of individuals with disabilities and families on commit- tees. |
| Callander | 2011 | Australia | Empirical | Health Research | Mental health | Adult | Given the paucity of literature describing partnership between consumers and carers as researchers, particularly in Australia, the aim of this study was to enable the consumer and carer research teams to reflect on their experiences of working together. |
| Callard | 2012 | United Kingdom | Non-Empirical | Health Research | N/A | N/A | The paper aims to develop a model of translational research in which service user and other stakeholder involvement are central to each phase. |
| Campbell | 2009 | United Kingdom | empirical | Health Planning/Service Design/QI | Learning disabilities | Adult | This paper reports on how people with learning disabilities and carers were included in national health review teams in Scotland... Planning and support arrangements are reported. The focus of this account is on the role and the people with learning disabilities in these review teams. experience of |
| Campbell | 2007 | United Kingdom | Empirical | Health Planning/Service Design/QI | HIV | Adult | This paper describes the process in the involvement of service users in the work of the North East London Sexual Health and HIV Clinical Network (NELNET) and the establishment of a network-wide user group called Patient's Voice. The paper discusses factors and challenges that must be addressed before meaningful user involvement in HIV services can be established. |
| Campbell | 2015 | South Africa | Empirical | Health Research | Schizophrenia | Adult | The aim of this article is to describe and consider the contributions made by a researcher-driven, population-specific CAB [community advisory board] in a genomics of schizophrenia research project taking place in South Africa, from the perspective of the research team. |
| Canfield | 2018 | Canada | Non-Empirical | Multiple | N/A | Adult | This Special Issue’s inspiring account of rapid achievement in patient engagement also exposes critical gaps in attempting to truly “democratize healthcare” (Coney and New Zealand Guidelines Group 2004; Staniszewska et al. 2008). How does self-selection bias the patient voice? What hidden barriers block participation? What do patients need to sustain their development as partners? Could investing in patient capacity reap rewards as effectively as with healthcare providers? Confronting difficult issues, as in this commentary, will help realize the shared ambitions of patient partners and healthcare providers for system transformation. |
| Carman | 2013 | United States of America | Non-Empirical | Health Research | Various | N/A | To propose a framework of patient engagement that represents the forms that patient engagement can take at different levels of the health care system, and that accounts for factors influencing engagement. |
| Caron-Flinterman | 2005 | Multiple Countries | Empirical | Health Research | N/A | Adult | This article therefore focuses on the added value of patient participation for biomedical research: what knowledge can patients contribute to the biomedical research process? |
| Caron-Flinterman | 2007 | Netherlands | Empirical | Health Research | N/A | Adult | In this article, we analyze strategies for patient participation and conclude that these can hardly be regarded as effective because they do not ensure patients' structural influence on decision-making |
| Carr | 2019 | Canada | Empirical | Health Planning/Service Design/QI | Arthritis | Adult | To describe the process of patient engagement to co-design a patient experience survey for people with arthritis referred to central intake. |
| Carroll | 2017 | Canada | empirical | Health Research | cardiovascular | Adult | This study assessed researchers' perceptions of the meaning and value of patient engagement in research within a Canadian cardiovascular research network. In doing so, the secondary aim was to inform the development of a structured patient engagement initiative by identifying potential challenges and related mitigation strategies. |
| Carter | 2018 | United Kingdom | Empirical | Health Policy/Governance | N/A | Senior/Older Adult | There is thus something of a gap between rhetoric and reality in the contemporary governance of the NHS, as public-facing documents maintain the narrative of public ownership and control of the service, while changes on the ground result in increasing fragmentation and attendant deficits in accountability (Martin et al., 2017). Our study seeks to examine how this tension came to bear on the activities of a group that found itself at a key nodal point within such debates: the PPI group that formed a key part of the ‘public engagement’ process following a regional healthcare transforma- tion initiative. |
| Cassels | 2016 | United States of America | Review | Health Technology Assessment | Female Hypoactive Sexual Desire Disorder | Adult | The purpose of this paper is to recognize the vital reasons for including public and patient voices in health policy decision-making, but illustrates the challenge it creates for decision-makers who must consider whether those voices represent patient interests or corporate interests. |
| Cawston | 2003 | United Kingdom | Non-Empirical | Health Planning/Service Design/QI | N/A | Adult | This paper offers an overview of theories of participation in their historical context and discusses some of the issues raised by applying approaches in primary care organisations. |
| Celenza | 2017 | United States of America | Non-Empirical | Health planning/Service Design/QI | neonatal intensive care | Pediatric | In this article, the authors review their experience as a field with family advisors in neonatal intensive care and particularly with regard to family involvement in quality improvement. |
| Chalmers | 2017 | Multiple Countries | Empirical | Health research | Bronchiectasis | Adult | The purpose of this article is to highlight the multiple aspects of clinical guidelines and clinical research where patients can make a positive contribution, to highlight the lessons learnt from the European Multicentre Bronchiectasis Audit and Research Collaboration (EMBARC) project in terms of how to support patient engagement, and finally to reflect upon the patients’ experience of these projects, in order to help future patients and professionals to do better. |
| Chambers | 2017 | United Kingdom | Empirical | Health Planning/Service Design/QI | Mental health | Adult | This article describes the involvement and views of service users in the development of a nursing metric—the Therapeutic Engagement Questionnaire. It presents their role in the three stages of development: generation, statement reduction and authentication. |
| Chambers | 2019 | Multiple Countries | Review | Health Research | Palliative care | Multiple | The primary aim was to systematically review the evidence regarding patient/carer involvement in palliative care research. The secondary aim was to identify facilitators, barriers and gaps in the evidence base. |
| Chamney | 2014 | United Kingdom | Non-Empirical | Multiple | Kidney | Adult | Describes development and activities of a service user advisory group, the Kidney Research and Education Initiative. |
| Chiu | 2013 | Canada | Empirical | Health Research | Breast cancer | Adult | The purpose of this article is to contribute to the literature on patient participation by sharing the details of a breast cancer study in which cancer patients/survivors were engaged in all stages of the study from question formation to conference presentations and study publication. |
| Chu | 2016 | United States of America | Non-Empirical | Other | N/A | Adult | We describe the benefits of patient partnership in academic medical conferences and how it can be enabled. |
| Chung | 2017 | United States of America | Empirical | Health Research | Type 1 diabetes | Multiple | To describe the resolution and impact of quandaries arising when patient stakeholders’ values and preferences conflicted with institutional policies. |
| Chung | 2006 | United States of America | Empirical | Health Research | HIV/AIDS | Adult | This paper presents a case study of a participatory process that was used to understand the needs of persons living with HIV/AIDS in a US state |
| Ciccarella | 2018 | United States of America | Non-Empirical | Health Research | N/A | N/A | We review and present current programs and projects that have successfully incorporated patients and advocates into translational research, and propose mechanism and strategies to actively engage patients throughout the research continuum. |
| Clarke | 2018 | United Kingdom | Empirical | Health Research | Dementia | Adult | We explore ways of involving people with experience as co-analysts and explore the role of reflexivity, multiple voicing, literary styling, and performance in participatory data analysis. |
| Clauser | 2015 | United States of America | Non-Empirical | Health Research | Oncology | Adult | This perspective paper provides an example of the potential for patients and stakeholders engagement |
| Clavel | 2019 | Canada | Empirical | Health Planning/Service Design/QI | Multiple conditions | Adult | The main goal of this research is to study key managerial practices to implement PP in QI and has two main objectives: 1-describe the implementation of a PP program in two different clinical areas; 2-identify managerial practices at different management levels used to implement PP in QI. |
| Clay | 2016 | Multiple Countries | Non-Empirical | Multiple | Critical illness | N/A | In this article, the authors draw on their personal experiences as patients in intensive care units (ICUs) and as patient advocates to share some of the complexities they have experienced as patient advocates in order to strengthen existing conversations about patient engagement. The aim is to survey the practical and ethical landscape so that solutions to various problems may be identified and solved as we move forward in our efforts to involve patients and their families in research, policy, and quality improvement in critical care medicine. |
| Clearfield | 2020 | United States of America | Non-Empirical | Multiple | N/A | Adult | To describe an approach to patient-centered COS development and discuss challenges and adaptations to improve engagement across COS projects. |
| Coates | 2016 | Australia | Empirical | Health Planning/Service Design/QI | Mental health problems | Adolescent | This article presents the findings of an evaluation of this YA (Youth Alliance). The purpose of this evaluation was to identify possible barriers to implementation early on so these could be addressed and a youth participation model that meets the needs of all key stakeholders could be developed. |
| Cohen | 2007 | United States of America | Empirical | Health Research | Parkinson's disease | Adult | This article provided examples of the human element in clinical trials and illustrated the need for authentic voices of patients in the development of new therapies |
| Coleman | 2015 | United Kingdom | Empirical | Health Research | Pressure Ulcer | Adult | This article describes the value of patient and carer views, and these were integrated into research to develop a new pressure ulcer risk assessment instrument - Pressure Ulcer Risk Primary or Secondary Evaluation Tool (PURPOSE T) - to support clinical decision making and promote patient safety. |
| Collier | 2016 | Australia | Empirical | Health Research | End-of-life patients | Adult | The aim of this article is to share our experiences of using VRE (video-reflexive ethnography) to research alongside patients and explain how iterative researcher reflexivity in the field was critical to the progress and success of our studies. We discuss how we juggled competing values through a continual process of reflexivity. |
| Collins | 2017 | United States of America | Non-Empirical | Health Research | Cardiovascular disease | Adult | The purpose of this article is to describe successful methods for researchers to identify and engage patients, caregivers, and stakeholders in the research process to better ensure that the research is relevant and understood by the individuals and groups for whom it is designed to benefit. |
| Collyar | 2005 | United States of America | Non-Empirical | Multiple | Cancer | Adult | This paper shows cancer patient advocates are involved in many diverse cancer research in the United States |
| Colombo | 2012 | Multiple Countries | Non-Empirical | Health Research | N/A | Adult | This paper describes: (1) different interventions for involving patients; (2) experiences to promote consumer evidence-based advocacy; and (3) barriers to consumer involvement in health system reforms, including vested interests in patients’ associations. Citizens’ involvement in health systems can vary substantially, but is gaining increasing weight |
| Compagni | 2006 | United States of America | Non-Empirical | Multiple | Mental health problems | Multiple | First, the authors will discuss the creation of a partnership between consumers and neuroscientists, and its implications for future mental healthcare services. Second, an analysis is provided for the current status of the knowledge about mental illness and genetics. |
| Concannon | 2012 | United States of America | Non-Empirical | Health Research | N/A | Adult | This paper presents a new taxonomy to guide researchers and stakeholders into a new era of stakeholder engaged research. |
| Cook | 2005 | Multiple Countries | Review | Multiple | N/A | Senior/Older Adult | This literature scan considers issues arising from these debates to examine the developments that have taken place in gerontological nursing practice to involve older people in their care, service planning and policy development. |
| Coon | 2016 | United Kingdom | Empirical | Health Research | ADHD | Adolescent | The aims of this study were to (i) highlight the methods of end-user involvement used in our reviews, (ii) to facilitate and stimulate discussion of the most appropriate and efficient methods of engagement and (iii) to develop a list of suggestions to improve future involvement in systematic reviews. |
| Cosper | 2018 | United States of America | Empirical | Multiple | Hospital ICU | Adult | The objective of this study was to determine if patient and family advisors (PFAs) collaboration in an educational program could increase the empathy levels of intensive care unit (ICU) nurses. |
| Costello | 2019 | Ireland | Empirical | Health Research | Rheumatic disease | Adolescent | To design a methodology for improving researcher‐adolescent communications specifically aimed at mutual relationship building for PPI. Deliberate and effective preparation in advance of research involvement to improve the downstream success of that involvement |
| Coulman | 2020 | United Kingdom | Empirical | Health Research | N/A | Adult | This paper aims to 1) describe how PPI functions in trial oversight, using multiple qualitative methods, and 2) contribute to recommendations for maximising PPI in clinical trials, in collaboration with public contributors with experience of involvement in trial oversight. |
| Coultas | 2019 | United Kingdom | Empirical | Health Policy/Governance | N/A | Adult | To understand how different members in clinical commissioning groups (CCGs) understand PPI as currently functioning in their decision‐making practices, and the implications of the STPs for it |
| Coupe | 2020 | United Kingdom | Empirical | Health Research | Multiple | Adult | The aim of this paper was to explore the impact of PPI on two health‐related doctoral research studies and identify how PPI could be used meaningfully at this level. |
| Cowan | 2011 | United Kingdom | Non-Empirical | Health Policy/Governance | Mental health problems | Adult | The paper’s purpose is to reopen a debate around the potential impact of narrow conceptualisations of inclusion, or participation, of service users in current mental health policy development and implementation |
| Cox | 2015 | United Kingdom | Empirical | Health Research | Mental health problems | Adult | The study offers a critical appraisal of the participation of mental health service users in research collaboration and the potential of cultural safety to concentrate researchers’ attention on power imbalance, cultural dominance and structural inequality informing research practices and mental health service users’ experience. |
| Cox | 2020 | Multiple Countries | Review | Health Research | N/A | Adult | The aim of this scoping review is to provide a snapshot of the current status of the important and emerging area of consumer engagement in occupational therapy health-related research, as published in the Australian Occupational Therapy Journal. |
| Crocker | 2016 | United Kingdom | Empirical | Health Research | N/A | Adult | To explore the views of PPI contributors involved in health research regarding the impact of PPI on research, whether and how it should be assessed |
| Crocker | 2018 | Multiple Countries | Review | Health Research | N/A | Adult | To investigate the impact of patient and public involvement (PPI) on rates of enrolment and retention in clinical trials and explore how this varies with the context and nature of PPI. |
| Cukor | 2016 | United States of America | Review | Health Research | Kidney disease | Adult | The goal of this paper is to synthesize the experiences of these studies to gain an understanding of how meaningful patient and stakeholder engagement can occur in clinical research of kidney diseases, and what the key barriers are to its implementation. |
| Cunningham | 2016 | United States of America | Non-Empirical | Health Planning/Service Design/QI | N/A | Adult | The authors examine ways in which the Magnet culture helped drive change and offer tips to establish a successful patient and family advisory council. |
| Curry | 2007 | United States of America | Empirical | Health Planning/Service Design/QI | N/A | Senior/Older Adult | To determine the presence, characteristics, and impact of Family Councils |
| Curtis | 2021 | Canada | Empirical | Education (Patient) | Kidney disease | Adult | The intent of this article is to outline the development and deployment of the Kidney Check program, with specific focus on the project team and patient engagement strategies. |
| Cusack | 2000 | United Kingdom | Non-Empirical | Health Policy/Governance | Occupational therapy | Adult | This paper presents current government policy on clinical governance, a working definition and an exploration of the value of user involvement. |
| Czulada | 2015 | United States of America | Empirical | Health Planning/Service Design/QI | Pediatric intensive care | Pediatric | The objective was to understand why communication between the clinical team and families was not occurring consistently in the pediatric intensive care unit and improve the system using a multidisciplinary improvement team including a family advisor. |
| Dadich | 2018 | International | Review | Health Research | Healthcare - associated infection | Adult | This review examines patient involvement in healthcare-associated infection (HAI) research. Healthcare-associated infections represent an intractable issue with considerable implications for patients and staff. Participatory methodologies that involve patients in health care research are associated with myriad benefits. |
| Darling | 2013 | United Kingdom | Empirical | Health Research | Dementia | Adult | To present the actions taken by the Scottish Dementia Clinical Research Network (SDCRN) to comply with Scotland’s National Dementia Strategy (SNDS) on patient and public involvement (PPI) |
| Dayton | 2016 | United States of America | Non-Empirical | Health Planning/Service Design/QI | Mental health (trauma) | Pediatric | This article explores barriers and strategies to achieving family driven integrated child health care. |
| de Souza | 2016 | United Kingdom | Empirical | Health Planning/Service Design/QI | Rheumatology | Adult | The aim focused on a patient-centred development of innovative strategies to improve the patient experience of rheumatology outpatient services |
| de Wit | 2020 | United States of America | Non-Empirical | Multiple | Rheumatic conditions | Adult | To assess how patient engagement can be useful in rheumatology research. |
| DeCamp | 2015 | United States of America | Empirical | Health Planning/Service Design/QI | N/A | Pediatric | The purpose of this study was to inform increased engagement of LEP Latino families in health care improvement by (a) describing the initial experiences and expectations for advisory board participation of low-income LEP Latina women who participated in the first year of a family advisory board for a pediatric primary care clinic and (b) describing the assessment these mothers made of board activities and impact after their first year of board participation. |
| Degeling | 2015 | Multiple Countries | Review | Multiple | N/A | Adult | In this study our aim is to survey the extent, range and nature of research activity using deliberative methods in public health and health policy, and characterize how deliberative publics are being constituted and situated within broader policy processes. |
| den Oudendammer | 2019 | Netherlands | Empirical | Health Research | N/A | Adult | We provide an overview of when, why, and how PP activities take place in HFs'(health-related research funding organisations) funding processes, share main challenges and identify possible solutions. |
| Deng | 2010 | Taiwan | Empirical | Health Policy/Governance | N/A | Adult | Our goals were to design a participatory method that suited newly democratic countries and to engage people in discussing national health insurance premium reform in Taiwan |
| Dent | 2011 | International | Non-Empirical | Health Planning/Service Design/QI | N/A | Adult | This paper addressed the question: in what ways would doctors’ greater involvement in hospital management contribute to users gaining a greater influence in the organisation and delivery of healthcare? |
| Desai | 2019 | United States of America | Empirical | Health Research | Mental health | Adult | This report describes a two-year initiative to improve the quality and quantity of participatory research being conducted in a department of psychiatry at a large medical school, which culminated in an innovative training program for both researchers and patients, developed in direct collaboration with stakeholders |
| Devonport | 2018 | United Kingdom | Empirical | Health Research | Obesity | Adult | This article presents a reflective account of Patient and Public Involvement (PPI) in the development of obesity and binge eating research. |
| Di Lorito | 2020 | United Kingdom | Empirical | Health Research | Dementia | Adult | To propose a model for good practice in co-researching with carers of people with dementia, by reporting and synthesizing the personal reflections of the academic and lay researchers around the methodological issues, benefits, and challenges of co-research. |
| Diamond | 2003 | United Kingdom | Empirical | Health Planning/Service Design/QI | Mental health | Adult | To audit the user involvement service standards. To identify further improvements for user involvement and to consider whether user involvement influenced the culture of care |
| Dillon | 2020 | United States of America | Empirical | Other | Rare diseases | Adult | To share an approach to consider when selecting patient advisors, in order to get the most actionable input into a product development team. |
| Domecq | 2014 | United States of America | Review | Health Research | N/A | Adult | In this systematic review we aimed to answer 4 key questions: what are the best ways to identify patient representatives? How to engage them in designing and conducting research? What are the observed benefits of patient engagement? What are the harms and barriers of patient engagement? |
| Dovey-Pearce | 2019 | United Kingdom | Empirical | Health Research | Transitions in care | Adolescent | This study describes the experiences of adult researchers and young people involved in a large-scale, UK health research programme, exploring the process of working together and the outcomes of that work. |
| d'Udekem | 2018 | Multiple Countries | Empirical | Health Research | Fontan circulation | Adult | This paper describes the involvement of patients and parents in Fontan research and the benefits of the involvement |
| Dudley | 2015 | United Kingdom | Empirical | Health Research | N/A | Adult | To explore researchers' and PPI contributors' accounts of the impact of PPI within trials and factors likely to influence its impact. |
| Duffett | 2017 | Canada | Review | Health Research | N/A | Adult | This review article presents justification, methods, and risks of patient engagement in clinical trials drawn from systematic review papers |
| Edwards | 2019 | Canada | Empirical | Health Research | Multiple conditions | Adult | To identify patient-generated priority topics for future primary care research in British Columbia (BC), Canada within a diverse patient population. |
| Elberse | 2011 | Netherlands | Empirical | Health Research | Congenital heart disease | Adolescent | To gain more insight into exclusion mechanisms and inclusion strategies in patient–expert partnerships. |
| Elberse | 2012 | Netherlands | Empirical | Health Research | Multiple conditions | Adult | This article describes a case study wherein the Health Council of the Netherlands involved patient groups in an advisory process with a predefined focus: setting a research agenda for medical products development |
| Eley | 2016 | United Kingdom | Non-Empirical | Health Policy/Governance | Dementia | Adult | The purpose of this paper is to highlight the need for proper involvement of people with dementia and carers in policy making and to suggest practical ways to achieve this. |
| Elliot | 2021 | Canada | Empirical | Education (Patient) | Chronic Kidney Disease | Adult | In this report, we describe these efforts to plan, implement and sustain KT initiatives across our large-scale health research network, which could serve as a useful roadmap for other organizations interested in achieving meaningful engagement of knowledge users, including patients in health research. |
| Enzinger | 2017 | United States of America | Empirical | Education (Patient) | Cancer | Adult | We describe the stakeholder-driven development and acceptability testing of a prototype video and companion booklet supporting informed consent (IC) for a common palliative chemotherapy regimen. |
| Evans | 2013 | United Kingdom | Non-Empirical | Health Research | N/A | Adult | We aimed to develop a standard operating procedure (SOP) to support researchers to involve service users in trials and rigorous studies. |
| Evette | 2020 | United States of America | Empirical | Health Research | Chronic musculoskeletal pain | Adult | The aim of this Lessons Learned report was to describe how we engaged diverse patients with potentially conflicting perspectives in a PAC for an evaluation of risk reduction initiatives for chronic opioid therapy for chronic musculoskeletal pain. |
| Ewalds | 2018 | Canada | Empirical | Health Planning/Service Design/QI | Mental health | Adult | The aim of this paper is to shed light on the implementation of a strategic organizational structure for patient engagement in mental health by examining why and how to implement a structure, the organizational and environmental factors that facilitate or limit the process, and the perceived consequences of the implementation. |
| Fairbrother | 2016 | United Kingdom | Empirical | Health Research | Stroke | Adult | To improve research design through patient involvement. |
| Fancott | 2018 | Canada | Non-Empirical | Health Planning/Service Design/QI | N/A | Adult | This article describes CFHI initiatives to enhance patient engagement efforts across Canada and the lessons learned in the context of “engagement-capable environments” and offers reflections for the future of patient engagement in Canada. |
| Faulkner | 2018 | United States of America | Empirical | Health Research | Atherosclerotic cardiovascular disease | Adult | This paper provides a description of how we implemented patient engagement in ADAPTABLE thus far, including a description of committee structures and composition, firsthand patient testimonials, specific contributions, and lessons learned during the planning and early implementation of ADAPTABLE. |
| Fernandes | 2019 | Multiple Countries | Review | Health Planning/Service Design/QI | N/A | Adult | To explore patient involvement in the implementation of infection prevention and control (IPC) guidelines and associated interventions |
| Fleurence | 2014 | United States of America | Non-Empirical | Health Research | Multiple conditions | Adult | This article describes the PCORI clinical and patient network, their potential uses, and the challenges they face. |
| Flowers | 2020 | Canada | Empirical | Health Planning/Service Design/QI | N/A | Adult | To assess the experiences and effectiveness of PFAs as research team members. |
| Forster | 2008 | United Kingdom | Review | Health Policy/Governance | N/A | N/A | In this article we focus on developments in England1 under New Labour, place them in the context of broader health policy, and assess them in the light of the available limited empirical evidence. We also consider a range of possible explanations for these developments. |
| Forsythe | 2014 | International | Review | Health Research | Rare diseases | Adult | To synthesize evidence about engagement of patients and other stakeholders in research on rare diseases, including the role of rare disease organizations in facilitating patient-centered re- search. |
| Forsythe | 2019 | United States of America | Empirical | Health Research | N/A | Adult | This article analyzes 126 peer-reviewed articles that include descriptions of PCORI research teams’ experiences with engagement. The questions guiding this review were: What are the contributions of engagement to PCORI-funded comparative effectiveness research? What engagement approaches did research teams use to achieve these contributions? How did research teams assess the contributions of engagement? |
| Forsythe | 2016 | United States of America | Empirical | Health Research | N/A | Adult | We aimed to characterize patient and stakeholder engagement in the 50 Pilots Project funded by Patient-Centred Outcomes Research Institute (PCORI) and identify early contributions and lessons learned. |
| Forsythe | 2018 | United States of America | Empirical | Health Research | N/A | Adult | This paper presents PCORI’s evaluation framework for assessing the short- and long term impacts of engagement; describes engagement in PCORI projects (types of stakeholders engaged, when in the research process they are engaged and how they are engaged, contributions of their engagement); and identiﬁes the effects of engagement on study design, processes, and outcomes selection, as reported by both PCORI-funded investigators and patient and other stakeholder research partners. |
| Foster | 2020 | Canada | Empirical | Health Research | Blood cancer | Adult | We aimed to partner with patients on each component and stage of clinical trial protocol development, and to better align clinical trial processes and resources with patient needs. |
| Fraenkel | 2016 | United States of America | Empirical | Health Research | Rheumatoid arthritis | Adult | We sought to determine the feasibility and value of developing CPG recommendations based on a voting panel composed entirely of patients, with the ultimate goal of comparing the patients’ recommendations to ones developed by a physician-dominated voting |
| Frank | 2015 | United States of America | Non-Empirical | Health Research | N/A | N/A | To provide an overview of PCORI’s approach to engagement in research. |
| Freedman | 2006 | United Kingdom | Review | Health Policy/Governance | N/A | N/A | This review looks at the national drivers for patient involvement, and the degree to which patients are involved in setting quality standards and monitoring of these quality standards. |
| Frost | 2018 | United Kingdom | Empirical | Health Research | Diabetes | Adult | To describe a novel approach to including patient partners in QDA (qualitative data analysis) ; to illustrate the kinds of contribution that patient partners made to QDA in this context; and to propose a characterization of a process by which patient involvement can contribute to knowledge production. |
| Fudge | 2007 | Multiple Countries | Review | Health Research | N/A | Senior/Older Adult | The purpose of this review is to establish the scope and extent of the involvement of older people in health research over the past 10 years, to identify reported barriers to the involvement of older people in research and to determine the impact of the involvement of older people on research and on participants. |
| Gagnon | 2014 | Canada | Empirical | Health Technology Assessment | Cancer | Adult | The project objectives are: 1) setting up interventions to promote patient participation in three stages of the HTA process: identification of HTA topics, prioritization, and development of the assessment plan of the topic prioritized; and 2) assessing the impact of patient participation on the relevance of the topics suggested, the prioritization process, and the assessment plan from the point of view of patients and other groups involved in HTA. |
| Gagnon | 2012 | Canada | Empirical | Health Technology Assessment | Isolation and restraint of hospitalized adults and elderly people in nursing homes | Adult | The aim of this research project is to use our frame- work to implement and then evaluate interventions involving patients in the assessment of alternatives to isolation and restraint for hospitalized or institutionalized adults. |
| Gagnon | 2021 | Multiple Countries | Review | Health Technology Assessment | N/A | Adult | This paper aims to synthesize knowledge on how patients and the public have been involved in HTA activities over the last decade and to propose a framework to assess the impact of PPI in HTA. |
| Gagnon | 2020 | Canada | Empirical | Health Research | Diabetes mellitus | Adult | Therefore, the present work provides a commentary, from our perspective (as patient partners), on our participation in this clinician-led, practice-based research project. |
| Garnett | 2017 | Canada | Empirical | Health Planning/Service Design/QI | Primary Care | Adult | The Academic Family Medicine Clinic at the South Health Campus in Calgary, Alta, identified a need for patient and community advisory expertise regarding clinic initiatives and quality improvement. A council was proposed to engage patients and citizens in exploring meaningful ways to drive innovation and improve the care experience. |
| Ghersi | 2002 | Australia | Non-Empirical | Health Research | N/A | Adult | This article discusses consumer involvement in the Cochrane review process in relation to the 10 key principles that guide the work of the Cochrane Collaboration: collaboration building on enthusiasm of individuals, avoiding duplication, minimizing bias, keeping up to date, striving for relevance, promoting access, ensuring quality, continuity and enabling wide participation |
| Giebel | 2019 | United Kingdom | Empirical | Health Research | Dementia | Senior/Older Adult | The aim of this paper is to disseminate the lessons we have learnt so far from setting up two patient and public involvement groups with people with dementia and their carers, one small face-to-face reference group and one virtual lay reference group, and to disseminate the methods used and benefits in shaping the research design of the programme, methods of data collection, intervention, and economic modelling analysis. |
| Gillard | 2012 | United Kingdom | Empirical | Health Research | Mental Health | Adult | In this article we aim to (a) describe the team process of qualitative data analysis undertaken in a study of support for self-care in mental health in the United Kingdom, (b) reflect on the extent to which we coproduced knowledge through the qualitative analysis process as a research team with a high level of patient and public involvement, and (c) consider the implications of research co production for study findings. |
| Gilmore | 2019 | United States of America | Non-Empirical | Health Research | Geriatric Oncology; Geriatric Assessment | Senior/Older Adult | Herein, we described our patient partner engagement in study optimization, shape, conduct, and the dissemination of research findings using PCORI's 6 principles as a guide: 1) reciprocal relationships; 2) co-learning; 3) partnerships; 4) transparency; 5) honesty; and 6) trust. |
| Goel | 2020 | United States of America | Empirical | Health Research | Psoriatic Arthritis | Adult | This paper reviews the general considerations related to PRP involvement in research endeavors. |
| Gold | 2005 | Canada | Empirical | Health Planning/Service Design/QI | Cancer | Adult | To explore the extent and manner of patient participation in the planning of regional supportive care networks throughout the province of Ontario. We consider the disconnect between the rhetoric and reality of patient involvement in network planning and coordination. |
| Gombeski | 2010 | United Kingdom | Empirical | Health Planning/Service Design/QI | N/A | Adult | The article provides an overview of how CAGs work, their advantages and disadvantages, tips on how to make them work better, and insights from interviews from 39 healthcare chief marketing officers on their use of CAGs |
| Gordon | 2017 | United Kingdom | empirical | Health Research | prostate cancer | adult | To explore the inclusion of patient and public involvement (PPI) in a qualitative study on the experiences of men with prostate cancer regarding information in radiotherapy. |
| Graham | 2017 | United Kingdom | Empirical | Health Research | Cerebral Palsy | Pediatric | This practice analysis aims to discuss the involvement of advocates, the contribution of this involvement, and the benefits and challenges of involving advocates within research. |
| Greene | 2018 | United States of America | Empirical | Health Planning/Service Design/QI | N/A | Adult | In our mixed-method study, we sought to identify the different ways that patient partners influence QITs and to document the extent of the impact of patient partners— from the perspective of both QIT leaders and patient partners. |
| Gregory | 2018 | United Kingdom | Empirical | Health Research | Dementia | Adult | Our aim in this paper is to explore the impact research participant involvement has within the PREVENT and the European Prevention of Alzheimer’s Dementia (EPAD) projects. |
| Grogan | 2012 | Ireland | Empirical | Health Planning/Service Design/QI | Haemophilia | Multiple | The purpose of this article/project is to describe the development of a patient partnership programme and its impact on quality improvements in a comprehensive haemophilia care service. |
| Groot | 2021 | Netherlands | Empirical | Multiple | Chronic Respiratory Disease | Adolescent | We, therefore, aim to explore the experiences of adolescents with a chronic respiratory disease who were involved in a newly established advisory youth council in a tertiary hospital. |
| Group | 2021 | United States of America | Empirical | Health Research | Hepatitis C (HCV) | Adult | The purpose of this article is to share our collective experiences as patients in the United States who have engaged in clinical research alongside investigators conducting two US studies funded by the Patient Centered Outcomes Research Institute. |
| Grundy | 2019 | United Kingdom | Empirical | Health Research | Mental Health | Adult | The aims of the paper are to provide an example of PI being deeply embedded in the development of a mental health PROM and to critically assess the contribution of expert service user involvement |
| Gurung | 2020 | Multiple Countries | Review | Health Planning/Service Design/QI | Pediatric services | Multiple | To examine the extent, range and nature of published research investigating the engagement of children/youth, families and the public in pediatric service improvement, to summarize key aspects of the research identified and to identify gaps to help inform future research needs |
| Gustavsson | 2019 | Sweden | Empirical | Multiple | Neonatal care, pediatric diabetes | Adult | In the present article, we address the challenges of patient involvement in health- care quality improvements by describing experiences from patients and healthcare professionals who collaborated in AR [action research] projects, by using EBCD [experience-based co-design] in pediatric care settings at a Swedish hospital. |
| Haarsma | 2015 | Netherlands | Empirical | Multiple | Palliative care | Senior/Older Adult | First, how do patient representatives and professionals in Dutch PCNs perceive the impact of public involvement in the PCNs? Second, what are the perceived facilitators and barriers associated with public involvement in PCNs, according to patient representatives and professionals? The aim of our study was to examine in depth the cur- rent practice of public involvement in palliative care. |
| Hacker | 2018 | United States of America | Review | Health Research | N/A | Adult | This article addresses the differences and similarities between stages, and how researchers can effectively engage patients and stakeholders throughout all stages. It focuses on the mechanisms, challenges, benefits, and future directions of patient and stakeholder engagement while providing examples of lessons learned from the body of evidence surrounding engagement |
| Haesebaert | 2020 | Canada | Empirical | Multiple | Primary care services | Adult | To assess the acceptability and feasibility of embedding advisory councils of clinicians, managers, patients and caregivers to conduct patient-oriented quality improvement projects in primary care practices |
| Hamakawa | 2021 | Japan | Empirical | Health Planning/Service Design/QI | N/A | Adult | The objective of this study was to investigate the practice of active involvement of patients in medical research in Japan through modification of a rare disease research platform that utilizes information and communication technology. |
| Hamilton | 2018 | Canada | Empirical | Health Research | N/A | Adult | To develop and examine the content and face validity of the patient engagement in research scale (PEIRS) for assessing the quality of patient engagement in research projects from a patient partner perspective. |
| Happell | 2007 | Multiple Countries | Review | Health Research | Mental Health | Adult | The purpose of this paper is to examine the literature relating to consumer involvement in mental health research with a view to articulating a model to guide this process. |
| Happell | 2019 | Multiple Countries | Empirical | Health Research | Mental health | Adult | the aim of this study was to explore the opinions and experiences of other mental health researchers from Australia and New Zealand regarding working collaboratively with consumers in the conduct of research, with the view to enhance understanding of how consumer involvement in research could be increased. |
| Happell | 2016 | International | Review | Health Research | Mental Health | Adult | Firstly, this review aims to identify the occurrence of PMHCR in studies concerned with improving physical health care and physical health. Secondly, and building on the first aim, where PMHCR has occurred, to examine the nature of physical health problems, the scope of PMHCR across the research process and the outcomes of these studies. |
| Happell | 2009 | Australia | Empirical | Multiple | Mental Health | Adult | The aim of this paper is to explore the development and introduction of a role for a consumer of mental health services within an academic institution, including achievements of the role and the principles contributing to successful implementation. |
| Happell | 2020 | Multiple Countries | Empirical | Health Research | Mental Health | Adult | The aim of this research was to better understand barriers to consumer research positions from the perspectives of non-consumer researchers who have worked collegially with consumer researchers. |
| Happell | 2018c | Multiple Countries | Empirical | Health Research | Mental Health | N/A | This qualitative study explored other mental health researchers’ perspectives on the role of power in collaborative research with consumers |
| Happell | 2018a | Multiple Countries | Empirical | Health Research | Mental Health | N/A | The aim of this study was to enhance knowledge and understanding of the benefits that other mental health researchers have identified from collaborating with consumers in research activities in Australia and New Zealand. |
| Happell | 2018b | Multiple Countries | Empirical | Health Research | Mental Health | Adult | To enhance understanding of the perspectives and experiences of non-consumer researchers in working collaboratively with consumers as researchers. |
| Harris | 2019 | Multiple Countries | Review | Health Research | Diabetes | Adult | We conducted a review to identify how different approaches to involvement are being used to design and adapt diabetes interventions, and whether involvement contributes to reduction of diabetes risk and improved self‐management. |
| Harris | 2015 | International | Review | Health Research | Diabetes | Adult | The objectives of this review are to map the existing literature on patient involvement in diabetes research and to develop a preliminary theory of the contexts that enable involvement in diabetes research by people with diabetes, carers and the wider |
| Harrison | 2019a | International | Review | Health Research | Multiple | Adult | The aims of this narrative review are to identify, quantify and summarize (a) the conceptual foundational principles of patient stakeholder engagement in re‐ search and (b) best practice activities to support these efforts. |
| Harrison | 2015 | United Kingdom | Empirical | Health Research | Stroke | Adult | To explore the experiences of patients and carers involved in patient and public involvement (PPI) activities for stroke research. |
| Harrison | 2019b | United States of America | Empirical | Health Research | Multiple | Adult | To describe strategies to recruit and support members from hard-to-reach groups on research-focused Patient and Family Advisory Councils (PFACs) |
| Harrison | 2018 | United States of America | Empirical | Health Research | Multiple | Adult | The aim was to describe barriers to patient and family advisory council (PFAC) member engagement in research and strategies to support engagement in this context. |
| Harrison | 2020 | United States of America | Empirical | Multiple | N/A | Adult | To systematically engage stakeholders to identify important questions of adult hospitalized patients and to create a prioritized research agenda for improving the care of adult hospitalized patients. |
| Haycock | 2013 | United States of America | Empirical | Health Planning/Service Design/QI | N/A | Adult | This article examines how this large health care system (80 hospitals) in 19 states mobilized the patient perspective into health care operations and patient-safety initiatives through a formalized process of PFACs. |
| Hayes | 2018 | United Kingdom | Empirical | Health Research | Dementia | Senior/Older Adult | This paper describes the mechanisms for embedding public and participant involvement in establishing and managing the project, outlines PPI related outcomes and reflects on the success of PPI activity in terms of future directions for BDR and for best pra |
| Healey | 2020 | Canada | Empirical | Health Planning/Service Design/QI | Deceased Organ Donations | Adult | This paper describes the process used to create fulsome patient partner engagement resulting in mutually beneficial policy development in this complex area. |
| Henderson | 2018 | Canada | Non-Empirical | Multiple | Mental Health | Adolescent | Our team is addressing these challenges by implementing and rigorously evaluating an integrated community- based collaborative care team (ICCT) model in Toronto, providing rapid access to youth-friendly, evidence-based services in a stepped-care manner |
| Hillier | 2020 | Canada | Empirical | Health Research | Chronic Kidney Disease | Adult | To establish a more integrated and patient-centered approach, one of Canada’s largest kidney research networks (Can-SOLVE CKD) has created a Research Operations Committee (ROC) that includes patients as key members |
| Hirst | 2016 | United Kingdom | Non-Empirical | Health Research | Emergency Care | Adult | In this article, we describe what public involvement is and how it can help emergency care research. We use the development of a pioneering public involvement group in emergency care, the Sheffield Emergency Care Forum, to provide insight into the potential and challenges of public involvement in emergency care research. |
| Hoeg | 2019 | Denmark | Empirical | Health Research | Breast Cancer | Adult | We examined how involving patients with lower education levels affected PPI in the development of the MyHealth randomized clinical trial of breast cancer follow-up from the perspectives of the patients and professionals. |
| Hoekstra | 2018 | Canada | Empirical | Health Research | Physical Disabilities | Adult | The aim of this study is to evaluate and explain the out- comes and impacts of the CDPP at the broader network level, as well as at the level of individual projects, using the RE-AIM framework. |
| Horsfall | 2007 | International | Non-Empirical | Health Research | Mental Health | Adult | In this paper, known and likely barriers to more active research involvement and reasons for consumers and carers to participate in research are discussed. Structural barriers and perceived and actual disadvantages of consumer and carer involvement in research are also outlined. Finally, practical considerations for research collaboration and factors that may contribute to successful collaborative mental health research are highlighted. |
| Hovey | 2010 | Canada | Empirical | Other | Patient Safety | Adult | Through the use of an interpretative phenomenological research approach, an inquiry into what patients believe is their role in safety was addressed. |
| Hruslinski | 2021 | Multiple Countries | Empirical | Health Research | Hip fracture | Adult | In this paper, we report our experiences of developing and implementing a patient engagement approach for an ongoing multicenter randomised trial comparing two standard-care approaches to anesthesia for major orthopedic surgery in older adults |
| Huang | 2017 | United States of America | Empirical | Multiple | Multiple (multiple chronic conditions, diabetes, and impairment | Adult | To describe the context and design of PATIENTS (Patient-centered Involvement in Evaluating the Effectiveness of Treatments) including: methods and activities to build a skilled PCOR community; reflect on experiences implementing this innovative capacity building project, and present preliminary evidence of success. |
| Hubbard | 2007 | Multiple Countries | Review | Multiple | Cancer | Multiple | This paper presents a summary of the main findings of a review of literature about involving people affected by cancer in research, policy and planning and practice, and highlights the gaps in this area, for example, the absence of involvement by key groups including, older people, people living in deprived and rural communities, and men. Specifically, the review aimed to answer: 1. Why are people affected by cancer involved in research, policy and planning, and practice? 2. How are people involved? 3. What influence does their involvement have? |
| Hughes | 2017 | Canada | Non-Empirical | Health Planning/Service Design/QI | Multiple | Adult | This paper provides comments on behalf of Patients for Patient Safety Canada(PFPSC) related to the paper "Five years of experience using front-line ownership to improve health care safety and quality (Gardam et al., 2017) |
| Hull | 2012 | United Kingdom | Non-Empirical | Multiple | Liver disease | Multiple | This paper reports the authors’ experience of setting up a patient and public involvement (PPI) panel to support research in liver disease at a large NHS foundation trust. The authors discuss how such a panel can influence clinical trials led by academic investigators and why PPI is essential for the implementation of such trials. How the PPI panel supports the vision of the National Institute for Health Research Birmingham Liver Biomedical Research Unit (NIHR BRU) and promotes public engagement and education is also discussed. |
| Humphries | 2020 | Sweden | Empirical | Health Planning/Service Design/QI | Myocardial Infarction with Nonobstructive Coronary Arteries (MINOCA) | Adult | This paper aims to describe the development of a therapist-guided, internet-delivered psychological intervention designed specifically for patients with MINOCA. |
| Hutchinson | 2007 | United Kingdom | Empirical | Multiple | Lung, breast or bowel cancer | N/A | The aim of this paper is (i) to describe and discuss the process of developing the audiovisual patient information (video, DVD and CD-ROM, which is specific to the randomized cancer trial setting) and (ii) to highlight the challenges and opportunities, thereby identifying implications for practice. |
| Hutchinson | 2013 | United Kingdom | Empirical | Multiple | Mental health issues | Multiple | This paper describes the process by which a group of service users with experience of mental health issues and the statutory care system transformed their lives through involvement within each stage of a research project |
| Hwang | 2019 | Multiple Countries | Empirical | Multiple | N/A | Senior/Older Adult | How and when consumer engagement is occurring in the clinical settings where AGS members provide care, and to identify opportunities to improve engagement |
| Iliffe | 2011 | United Kingdom | Empirical | Multiple | Dementias, Parkinson’s disease and other neurodegenerative diseases | Adult | What benefits (if any) does PPI in research bring to the research process? In attempting to answer this question, we report here three examples of how the DeNDRoN Coordinating Centre has helped with PPI in 3 individual studies, when asked to do so. |
| Iwata | 2019 | United States of America | Empirical | Multiple | Head/neck cancer | Multiple | The purpose of this article is to describe the major benefits of patient-driven research in the field of head & neck oncology, to review lessons learned from establishing and sustaining partnerships with patients and caregivers, and to serve as a model for further patient-driven research endeavors. |
| Iyer | 2015 | United States of America | Empirical | Health Planning/Service Design/QI | Mental health issues | N/A | this project used a community-partnered participatory research framework to create a stakeholder-based general medical and wellness intervention in a large CMH organization, with consumers involved in all decision-making processes. |
| Jackson | 2020 | United Kingdom | Empirical | Multiple | Asthma | Adult | As an exemplar, we reflect on how, in the Asthma UK Centre for Applied Research (AUKCAR), we setout to create a supportive, organised environment with the overarching value of ‘keeping patients at the heart of everything we do |
| James | 2000 | United Kingdom | Non-Empirical | Health Policy/Governance | N/A | Multiple | Author comments on a survey of lay members on primary care groups and local health groups in England and Wales. |
| Janamian | 2016 | Australia | Non-Empirical | Multiple | N/A | N/A | [Author comments on] the role of consumers extending beyond being passive health care recipients and even active participants in their own care to involvement in innovation and value co-creation in health care. She advocates: for active dialogue to occur in co-creation, consumers must become equal partners with health care organisations and providers, with the focus on areas of interest to all parties. |
| Janney | 2017 | United States of America | Empirical | Multiple | N/A | Adult | This report summarizes mental health service users' current physical activity practices, beliefs and barriers; their experiences with physical activity counseling by mental health clinicians; and their preferences for physical activity-related services with the goal of informing the development of physical activity services for mental health service users at a mental health clinic. |
| Janvier | 2019 | Canada | Non-Empirical | Health Research | N/A | Pediatric | This article provides an overview of activities that can be performed by resource parents in neonatal research and illustrates with examples how the involvement of parents and families is essential for the future of neonatal research. |
| Jennings | 2018 | United Kingdom | Review | Health Research | Mental health issues | Adult | The aims of this study were (1) to develop a methodology for involving PPI co-researchers in analysis of qualitative data; (2) to pilot and refine this methodology; and (3) to create a best practice framework for future PPI in data analysis. |
| Jewell | 2019 | United Kingdom | Empirical | Multiple | Mental illness | Adult | The aim of this study is to describe the creation and formative evaluation of a service user and carer advisory group, set-up for the purpose of providing PPI for mental health data linkage projects being conducted within the Maudsley biomedical research centre |
| Jha | 2009 | Multiple Countries | Review | Multiple | N/A | N/A | The aim of this review is to provide updated integrated evidence on the role of the involvement of patients in medical education |
| Johnson | 2016c | Multiple Countries | Review | Health Planning/Service Design/QI | N/A | Multiple | This paper describes emergent mechanisms and processes that ambulatory care practices use to partner with patients and families in QI including outcomes, facilitators, and challenges. |
| Johnson | 2016b | United States of America | Non-Empirical | Health Research | breast cancer | Adult | Our work aims to produce relevant results to all end-users within the breast imaging community. In this commentary, we discuss the evolution of our roles and the patient partners’ impact on a breast cancer study's research activities to fill the knowledge gap in patients’ perspective to equitable engagement in research. |
| Johnson | 2016a | United States of America | Non-Empirical | Health Planning/Service Design/QI | N/A | Adult | This article is intended to review the purpose and role of PFACs in the context of improving health care delivery and highlight specific examples of the successful utilization of patient advisors to improve patient experience in the department of radiology. |
| Johnstone | 2009 | Multiple Countries | Review | Health Planning/Service Design/QI | N/A | N/A | The importance of recognising and responding to the critical relationship between culture, language and patient safety outcomes, and the possible benefits and risks of engaging patients of minority ethnic backgrounds in safety partnership programs are explored. |
| Jones | 2019 | United States of America | Empirical | Health Planning/Service Design/QI | N/A | Adult | The purpose of this article is to describe the creation of a toolkit aimed at strengthening health-care organizations’ abilities to advance health equity through PFACs. This resource, cocreated with representatives from diverse PFACs, identifies and promotes strategies to recruit and retain diverse representation in advisory councils. |
| Jones | 2017 | Finland | Non-Empirical | Multiple | N/A | Multiple | The questions we pose in this study are (1) what categories do policies construct for patients and the public in relation to involvement and (2) how do these different categorisations orientate involvement activities? |
| Jones | 2007 | United Kingdom | Non-Empirical | Multiple | Mental health | Adult | Em Jones (service user) and Sue Hahn (researcher) describe how they have been working together, delivering training on sexuality and mental health, and self-harm. |
| Joss | 2016 | Multiple Countries | Review | Multiple | People with disabilities | Adult | To conduct a scoping review on end user involvement in disability research, service and policy development. This review was conducted to improve discussion between members of a stakeholder advisory group about involvement of end users in the implementation a neuro- trauma research strategy (NRS). |
| Kaiser | 2017 | United States of America | empirical | Health Research | N/A | Adult | This article describes the Community Advisors on Research Design and Strategies (CARDS) model, a partnership developed between the University of Wisconsin-Madison School of Nursing and two community centers to deliberately engage hard-to-reach people in two lay advisory groups |
| Kang | 2020 | Multiple Countries | Review | Education (Health Professions) | Mental health | Adult | This integrative review analyzed the research on consumer involvement in mental health nursing education in the last decade. Research questions: What were the attributes of consumer involvement in mental health nursing education in the last 10 years? What were the outcomes of consumer involvement in mental health nursing education for nursing students in the last 10 years? |
| Karazivan | 2015 | Canada | Non-Empirical | Multiple | N/A | Adult | Authors describe this innovative approach to patient care, including the conceptual framework used in its development and the main achievements of patient partners in education, health care, and research. |
| Katz | 2012 | United States of America | Empirical | Multiple | Cancer/Leukemia | Multiple | We sought to evaluate the role and value of patient advocates in the cancer clinical trials process from the perspective of CALGB patient advocates and cooperative group investigators (committee chairs, co-chairs, study chairs, and protocol coordinators |
| Kelly | 2017 | United States of America | Empirical | Multiple | breast cancer | Adult | Authors conducted a process evaluation of ongoing activities of a Patient Advisory Committee (PAC) formed around the development of an individualized decision aid for older women with early stage breast cancer. |
| Kemper | 2013 | United States of America | Non-Empirical | Multiple | N/A | Adult | Roles to support the development of a family engagement program, methods to evaluate the level of family engagement, and strategies to enhance and sustain family engagement in patient safety initiatives are described. |
| Khodyakov | 2020 | United States of America | Empirical | Health Policy/Governance | Duchenne muscular dystrophy (DMD) | Adult | This paper illustrates how an online modified-Delphi approach could be used to engage patients, caregivers, and other stakeholder in CPG development |
| Kidd | 2007 | Australia | Empirical | Multiple | Mental health issues | Adult | The aims of this study, in rural Victoria, were to explore the perceptions of consumer advocates and clinicians about the concept of consumer participation in two mental health services and to examine how broad consumer participation policy initiatives are being enacted at a service delivery level. |
| Kimminau | 2018 | United States of America | Empirical | Health Research | N/A | Adult | To examine how various perspectives and diverse training lead investigators and patients to conflicting positions on how best to advance patient engagement. |
| Kirwan | 2005 | Multiple Countries | Empirical | Health Research | rheumatoid arthritis | Adult | Through a series of meetings and discussion sessions a research agenda emerged and this report outlines progress made on this agenda. |
| Kirwan | 2017 | Multiple Countries | Empirical | Health Research | rheumatoid arthritis | Adult | we describe and discuss different experiences of integrating patients as full patient research partners (PRPs) in out-comes research from multiple perspectives (e.g., researcher, patient, and funder), drawing from three real-world examples |
| Klein | 2016 | Canada | Empirical | Health Research | N/A | Adult | In this report, the author comments on how to incorporate patient input into benefit-risk assessment and regulatory decision making throughout the life cycle of therapeutic products in Canada |
| Knaapen | 2016 | Canada | Empirical | Health Planning/Service Design/QI | N/A | Adult | introduce key literature that clarifies the analytical lens through which we view the relation between abstract conceptual models and complex empirical practices; we describe the literature and fieldwork from which we identified and circumscribed three key conceptual models to support a structured, critical analysis of PPI in clinical guidelines; models are fleshed out and their rationale comparatively analysed: they draw, respectively, on lay participation in clinical care (Consumer Choice), health policy development (Democratic Voice) and medical knowledge production (Lay Expertise); by introducing a distinction between being and acting like a representative, we make these contradictions more explicit; discuss whether PPI models can—and should—work as gold standards to be followed in practice. |
| Komporozos-Athanasiou | 2018 | United Kingdom | Empirical | Health Research | Multiple (stroke, cancer and pre-term birth) | Multiple | Drawing on ethnographic data of PPI in three clinical areas we investigate citizen participation in health research as political ritual. We identify tensions between policy driven and ground-level performance of citizenship, and use ritual theory to show how such tensions are accommodated in participatory structures. We draw wider sociological implications for citizen participation beyond the health arena. |
| Koniotou | 2015 | United Kingdom | Empirical | Health Research | N/A | Senior/Older Adult | We implemented a model to involve service users in a multi-centre randomised controlled trial in pre-hospital emergency care. |
| Kovacs Burns | 2008 | Canada | Non-Empirical | Health Policy/Governance | multiple | Multiple | This articles provides an overview of patient safety champions' experiences with patient safety practices in different healthcare settings and their activities in helping to improve patient safety |
| Kreindler | 2009 | Canada | Non-Empirical | Multiple | N/A | N/A | This article outlines the implications of the ‘‘politics of methodology’’ for decisions about how best to involve patients in the design and evaluation of health services. |
| Kuehn | 2017 | United States of America | Empirical | Health Policy/Governance | N/A | N/A | This study examined the policy process in which patient experience data are collected by patient advocates and provided to FDA for regulatory decision making |
| Kushner | 2014 | Canada | Review | Multiple | N/A | N/A | Brief review of: history/context for patient and family member involvementin health care safety improvements; tools/mechanisms for patient engagement; specific examples of engagement from Canadian Patient Safety Institute; barriers/facilitators to patient engagement. |
| Kwon | 2017 | United States of America | Non-Empirical | Health Research | N/A | N/A | This article seeks to provide understanding and examples of how to apply core principles of community-based participatory research (CBPR) in developing patient-centered outcomes research (PCOR) that can impact clinical and public health practice. Authors review CBPR principles and demonstrate how to translate them into effective PCOR strategies |
| LaBoube | 2012 | United States of America | Empirical | Multiple | Mental health illness | N/A | This article focuses on how involvement in program evaluation promotes recovery Based on a review of the literature and the experiences at a psychosocial resource center for veterans, the process of involving people in recovery in program evaluation is illustrated with respect to several key recovery components. Challenges/lessons learned involving people in recovery in the program evaluation process and promoting this process to other staff members are described. |
| Lammers | 2004a | Australia | Empirical | Health Research | Psychiatric disability | Adult | This paper describes the establishment and function of a reference group, established to guide and assist with the conduct of a research project examining the experiences of consumers and carers with psychiatric disability support services. The formation and operation of, and the valuable contribution made by, the reference group is discussed. |
| Lammers | 2004b | Australia | Empirical | Health Research | Psychiatric disability | Adult | This article presents findings from a qualitative research study investigating the perceptions of consumers and carers regarding the degree to which the contemporary service system allows for their increased participation |
| Larsen | 2016 | Norway | Empirical | Health Planning/Service Design/QI | Mental health substance abuse | Adult | The aim was to explore what may hinder patients’ voices being heard when collaborating with staff and leaders to improve services. |
| Lauckner | 2012 | Canada | Empirical | Multiple | Health education focus (participants have multiple conditions (diabetes, chronic pain, multiple sclerosis, cancer, stroke and rare conditions)) | Multiple | The purpose of this study was to explore the experiences of health mentors in the Dalhousie Health Mentors Programme and to better understand the impact of participation in this programme on their lives. |
| Laurance | 2014 | Multiple Countries | Non-Empirical | Health Planning/Service Design/QI | Multiple (mental health; genetic disease; maternal/child health; patient safety) | Multiple | This article presents four case studies from around the world that highlight the proven and potential abilities of increased patient engagement to improve health outcomes and reduce costs, while extending the reach of treatment and diagnostic programs into the community |
| Lavallee | 2019 | United States of America | Empirical | Health Planning/Service Design/QI | Advanced heart failure | Adult | This short report describes the development of a Community and Patient Advisory Team (CPAT) formed to support patient involvement in interprofessional collaborative practice. |
| Lavallee | 2020 | United States of America | Empirical | Multiple | Advanced heart failure | Adult | This short report describes the development of a Community and Patient Advisory Team (CPAT) formed to support patient involvement in interprofessional collaborative practice. |
| Lavoie-Tremblay | 2016 | Canada | Empirical | Multiple | N/A | Adult | This article presents the experiences of patients engaged in co-designing care under a program entitled, ‘‘Transforming Care at the Bedside,’’ |
| Leese | 2018 | Canada | Empirical | Health Research | Arthritis | Multiple | To better understand, based on patient partners’ experiences, benefits and risks in patient partner–researcher relationships in a health research setting. |
| Lefebvre | 2018 | Canada | Empirical | Health Planning/Service Design/QI | Cancer | Adult | This article presents the main results produced in the evaluation phase of the a research project that brought together patient partners, nurse leaders from six clinical settings in Quebec and researchers to develop and test a web technology, the Forum for Knowledge Exchange (FKE), in order to improve discharge planning practices and oncological care transitions |
| Leff | 2021 | United States of America | Empirical | Health Research | Homebound older adults | Senior/Older Adult | Our aim was to engage homebound older adults and their caregivers to develop a patient- and caregiver-centered research agenda relevant to their needs and perspectives. |
| Légaré | 2009 | Canada | Review | Multiple | Multiple | Adult | This study aims at identifying what it is about patients and public involvement programs that works, in which contexts are patients and public involvement programs most likely to be effective, and how are patients and public involvement programs assumed to lead to better clinical practice guidelines development and implementation. |
| LeMaster | 2020 | Multiple Countries | Non-Empirical | Multiple | Multiple conditions | Multiple | In this issue, a number of studies illustrate the benefits of PPIE at various stages throughout intervention studies. |
| L'Esperance | 2021 | Canada | Empirical | Health Research | Multiple (primary care, diabetes, chronic pain, mental health, developmental disability, chronic disease and kidney diseases) | Adult | This project aims to build a national adaptable framework for the evaluation of PPE in research by: 1) building consensus on common evaluation criteria and indicators for PPE in research; 2) defining recommendations to implement and adapt the framework to specific populations |
| Lessard | 2020 | Canada | Empirical | Health Research | HIV | Adult | This article explores how stakeholders applied the guiding principles of a PE project ("co-build", "support and mutual respect", and "inclusiveness") for an HIV clinical research program initiated in January 2016. |
| Lindblom | 2021 | Sweden | Empirical | Health Planning/Service Design/QI | N/A | Adult | The aim of this study was to describe how user participation manifests itself within a co-design process involving patients, significant others and health-care professionals, including potential enablers or barriers. |
| Linhorst | 2001 | United States of America | Empirical | Health Planning/Service Design/QI | Mental health | Adult | To add to the body of literature pertaining to consumer involvement in public psychiatric hospitals by describing the consumer council established among clients residing at St. Louis Psychiatric Rehabilitation Centre |
| Livingston | 2013 | Canada | Empirical | Multiple | Mental health | Adult | To support patients' recovery and improve their experiences of care in a Canadian forensic mental health hospital, an intervention was launched to increase patient engagement by establishing a peer support program, strengthening a patient advisory committee, and creating a patient-led research team. The goals of the intervention were to (a) implement a range of patient engagement strategies in a forensic hospital, (b) assess whether the strategies improved patients’ experi- ences of care, and (c) evaluate whether such strategies produced positive outcomes for patients and the broader service system. |
| Lock | 2011 | Australia | Empirical | Health Policy/Governance | Indigenous health policy | Adult | To determine and describe the features of Indigenous participation in an informal national Indigenous health policy network in Australia |
| Lopatina | 2019 | Canada | Empirical | Health Planning/Service Design/QI | Rheumatoid arthritis | Adult | To describe a case study of patient involvement in redesigning a centralized system for intake of referrals from primary care to rheumatologists for patients with suspected rheumatoid arthritis |
| Lopes | 2015 | Australia | Empirical | Health Technology Assessment | N/A | Adult | To report on findings of a research project that explored the views of patient organization representatives and members of Advisory Committees providing advice to the Australian Deparrment of Health (DoH) on decisions related to public funding for new health technologies |
| Loud | 2013 | United Kingdom | Empirical | Health Planning/Service Design/QI | Chronic kidney disease | Adult | To describe and evaluate the role of the patient advisory group in a quality improvement project that aimed to improve the variation in primary care for people with chronic kidney disease stages 3-4 |
| Ludwig | 2020 | Multiple Countries | Review | Health Research | Frail and/or seriously ill | Adult | The aim was to synthesize the evidence on the engagement of frail and/or seriously ill patients as research partners across the research cycle. |
| Macarthur | 2021 | Canada | Empirical | Health Research | Child health | Multiple | The purpose of this paper is to describe the development of a curriculum to support capacity development in patient-oriented research in child health. |
| MacSweeney | 2019 | United States of America | Non-Empirical | Health Research | Youth mental health | Adolescent | To describe emerging practices of youth involvement in pediatric research and outline how such practices can be extended to the domain of youth mental health |
| Mader | 2018 | United Kingdom | Non-Empirical | Health Research | Various, including polycystic kidney disease, rare diseases, mental health | Adult | To report the establishment, experiences, and progress of the Patient Led Research Hub |
| Majid | 2019 | Multiple Countries | Non-Empirical | Health Planning/Service Design/QI | N/A | Adult | To investigate how studies have conceptualized and differentiated between degrees of engagement in planning and designing of health services |
| Malfait | 2018 | Belgium | Empirical | Health Planning/Service Design/QI | N/A | Adult | To identify conditions that contribute to the actual involvement of patients and the public in the decision-making processes of hospital policy through a stakeholder committee |
| Malterud | 2020 | Multiple Countries | Review | Health Research | Multiple conditions | Adult | We aimed to map out the scope and type of health research studies with patients involved as co-researchers throughout the research process and to explore the outcomes and experiences of such research. |
| Mamzer | 2017 | France | Empirical | Health Research | Cancer | Adult | To develop an empirical ethical research action that aims to improve patient representatives' involvement in the development of a translational research program together with health professionals. The aim is to promote common understanding and knowledge sharing between all parties, and to establish a long-term partnership integrating patients' expectations. |
| Manafo | 2018 | Multiple Countries | Non-Empirical | Health Research | N/A | Adult | To conduct a scoping review to identify methods for and outcomes of patient engagement in health research |
| Mandel | 2005 | United States of America | Empirical | Health Planning/Service Design/QI | N/A | Adolescent | To describe a 34-week pilot project aimed at improving health care service delivery for adolescents by offering youth a distinct role as advisory board members who help shape policy, provide feedback, guidance, and direction to a school-based health centre program in Boston |
| Markle-Reid | 2021 | Canada | Empirical | Health Research | Aging | Senior/Older Adult | This paper describes the lessons learned from a patient-oriented research program, the Aging, Community and Health Research Unit (ACHRU) on how to engage older adults with multimorbidity as research partners. Over the past 7-years, over 40 older adults from across Canada have been involved in 17 ACHRU projects as patient research partners. |
| Martini | 2019 | Mali | Empirical | Health Policy/Governance | Diabetes and HIV/AIDS | Adult | To explore the factors that influence the engagement of patient associations at the policy level, with a focus on the openness of the institutionalized political system and the role that public authorities, caregivers, and donors give to diabetes and HIV/AIDS patients |
| Mathie | 2018 | United Kingdom | Empirical | Health Research | Multiple | Adult | To explore the variation, types, importance of, and satisfaction with feedback given by researchers to PPI contributors in six PPI groups |
| McCarron | 2021 | Canada | Non-Empirical | Health Research | N/A | Adult | The objective of this commentary is to describe the involvement of four patient partners who worked with researchers during a scoping review. |
| McClean | 2017 | Australia | Non-Empirical | Health Planning/Service Design/QI | Primary care | Adult | To outline the process used by the Gold Coast Primary Health Network to design, implement, and operate a Community Advisory Committee |
| McDaid | 2009 | Ireland | Empirical | Health Planning/Service Design/QI | Mental health | Adult | To present evidence from participatory action research with Irish mental health service users that explored how they could more equally participate in advisory committees |
| McGrady | 2018 | United States of America | Empirical | Health Planning/Service Design/QI | Cancer | Multiple | To describe the creation and implementation of a novel, developmentally appropriate, and efficient Young Adult Advisory Program for cancer |
| McMillan | 2009 | Ireland | Empirical | Health Planning/Service Design/QI | Mental health | Adult | To highlight the work of a group of mental health service users, bereaved families, and carers in north and west Belfast, with a focus on their "card before you leave" appointment system for mental health patients, which has been adopted by the Northern Ireland Department of Health, Social Services, and Public Safety |
| Melchior | 2020 | Netherlands | Empirical | Health Research | Palliative care | Adult | To understand different PPI cultures among research teams and the impacts of PPI associated with each culture type. |
| Mercer | 2020 | Canada | Empirical | Health Technology Assessment | Multiple conditions | Adult | This article explores experiences and perceptions among patient groups participating in the Canadian Agency for Drugs and Technologies in Health (CADTH)’s pan-Canadian Oncology Drug Review (pCODR) process. |
| Merkel | 2016 | United States of America | Empirical | Health Research | Rare diseases | Adult | To outline the roles that patients and patient advocacy groups play in the rare diseases clinical research network, and to report on the patient advisory group's impact on the network's success |
| Mescouto | 2020 | Australia | Non-Empirical | Health Planning/Service Design/QI | Musculoskeletal pain conditions | Adult | In this editorial, we provide one example of how we are partnering with patients (and clinicians) in our current research. We aim to encourage research-ers to foster patient partnership in musculoskeletal research and share with potential patient partners how patients were engaged in our research. |
| Miller | 2020 | Multiple Countries | Review | Education (Health Professions) | Mental health/psychiatry | Adult | To review published literature and inform a critical discussion evaluating the role of patients and carers in psychiatric education. |
| Mjøsund | 2017 | Multiple Countries | Empirical | Health Research | Mental health | Adult | To examine how service user involvement can contribute to the development of interpretive phenomenological analysis methodology and enhance research quality |
| Morris | 2021 | United Kingdom | Empirical | Health Planning/Service Design/QI | Patient Safety | Adult | The aim of this study was to develop the Patient Safety Guide for Primary Care (PSG-PC) to support patients and carers to identify key patient safety issues and identify key points where they can make their care safer in primary care, co-designed by patients and carers and health-care professionals to support their involvement in primary care patient safety and to ensure the content of the PSG-PC is acceptable and accessible. |
| Moser | 2021 | Netherlands | Empirical | Health Planning/Service Design/QI | Cancer | Senior/Older Adult | The aim of this study was twofold: first, to improve the cancer care pathway experience of older cancer patients and, second, to explore lessons learned regarding how to involve this vulnerable group. |
| Moulon | 2010 | Multiple Countries | Non-Empirical | Health Policy/Governance | Various | Adult | To describe how the Patients' and Consumers' Working Party of the European Medicines Agency operates, and to give a detailed overview of the interaction between the agency and patients' and consumers' organizations focusing on the main achievements to date |
| Mwinga | 2015 | South Africa | Empirical | Health research | N/A | Adult | This paper draws on the perspectives of both research team members and community advisory board (CAB) members from research groups who used CABs in Lusaka. |
| Nambisan | 2009 | International | Non-Empirical | Health Planning/Service Design/QI | Various | Adult | To identify four alternate models of consumer value cocreation in health care and discuss their implications for health care organizations |
| Nancarrow | 2004 | United Kingdom | Empirical | Health Planning/Service Design/QI | Podiatry conditions | Adult | To systematically capture the learning from its Podiatry Patient Panel experiences, reflect on the benefits and pitfalls of the approach, and be able to provide this information to support other services interested in implementing a similar model |
| Naqshbandi | 2016 | Canada | Empirical | Health Planning/Service Design/QI | Chronic disease (type 2 diabetes) | Adult | To detail the methods, tools, and activities of the FORGE AHEAD Program, which is a 5-year research program that incorporates activities designed to foster community-driven initiatives with type 2 diabetes mellitus |
| Nathan | 2014 | Australia | Empirical | Health Planning/Service Design/QI | N/A | Adult | To examine the underlying assumption that legitimacy is the major pathway to influence for community representatives |
| Needham | 2021 | Canada | Empirical | Health Research | Cancer | Adult | This document describes the method taken by the Canadian Cancer Trials Group to implement meaningful patient centricity and engagement and the benefits realized. |
| Nelson | 2016 | Canada | Empirical | Health Planning/Service Design/QI | Mental health | Adult | To identify opportunities, challenges, and strategies that promoted or hindered collaboration among different stakeholders at 5 project sites across Canada in the planning of At Home/Chez Soi, a Housing First initiative for homeless people with mental health problems |
| Nguyen | 2018 | United States of America | Empirical | Health Research | N/A | Adult | In this article, we describe the pedagogical approach to design, implementation and evaluation on the use of the I-COREE curriculum as an education and training method (i.e. perspectives and experiences of community partners and academic researchers). |
| Nierse | 2012 | Netherlands | Empirical | Health Research | Chronic Kidney Disease | Adult | To examine the dynamics and dialogues in a collaboration between patient research partners and professional researchers |
| Nikiphorou | 2017 | Multiple Countries | Non-Empirical | Health Planning/Service Design/QI | Rheumatic diseases | Multiple | To discuss the recent evolution of the physician-patient relationship over time in Europe, reflecting on the 'journey' from behind the clinic walls through to clinical and research collaborations at national and international levels and the birth of healthcare professional and 'rheumatic' patient organizations |
| Norburn | 2020 | United Kingdom | Non-Empirical | Health Technology Assessment | Multiple conditions | Adult | In this commentary, we chart the evolution of patient involvement in HTAs at NICE over the past 20 years. We discuss how the role of patient evidence and lay membership of the committees who develop NICE guidance has evolved, and the value and impact that has had. |
| Nyirenda | 2018 | Malawi | Empirical | Health Research | Various | Adult | To report findings from an ethnographic study in Malawi that seeks to understand the purpose, relevance, and benefits of community engagement as seen by different stakeholders in research |
| Ochocka | 2002 | Canada | Empirical | Health Research | Mental health | Adult | To present value-driven strategies that were successful in reducing power differences between professional and consumer/survivor researchers in a research study |
| O'Donnell | 2019 | Ireland | Empirical | Health Planning/Service Design/QI | Acute frailty in older patients | Multiple | To present a systematic approach to involving public and patient representatives in the co-design of care pathways for acute frail older patients |
| Okun | 2017 | United States of America | Empirical | Multiple | Various | Multiple | To describe the development of the Patient and Caregiver Journey Framework and related patient-informed principles for design and measurement created by PatientsLikeMe in partnership with patients and caregivers using qualitative research methods, immersive observation, and directed one-on-one conversations |
| Oldfield | 2019 | Multiple Countries | Review | Multiple | Various | Multiple | To conduct a systematic review to characterize the impact of patient and family advisory councils (PFACs) on health systems |
| Oliver | 2009 | United Kingdom | Empirical | Health Technology Assessment | Various | Adult | To describe the input and influence of public involvement in setting the agenda for a national research program |
| Opava | 2012 | Sweden | Empirical | Multiple | Rheumatic conditions | Adult | To describe the role of patient organizations in musculoskeletal care |
| Oxland | 2020 | Canada | Empirical | Health Research | Multiple conditions | Adult | To understand the experiences of critically ill patients and their families. To identify opportunities to improve the quality of ICU care for patients and families |
| Pagura | 2018 | Canada | Empirical | Health Planning/Service Design/QI | Child and youth rehabilitation | Multiple | To describe an innovative partnership that builds capacity among clients and families and provides a foundation for other organizations to model in their approach to improve, transform and provide quality and safe care |
| Parry | 2020 | Canada | Empirical | Health Research | N/A | Adult | The overall goal of this project was to disseminate sex/gender knowledge and build capacity for patient engagement in clinical trials. Specific objectives were to 1) create capacity and opportunities for patient engagement and sex/gender knowledge/uptake in clinical trials and sponsor or investigator led activities and 2) enhance new/early investigator sex/gender knowledge and skills related to POR. |
| Patterson | 2014 | United Kingdom | Empirical | Health Research | Mental health | Multiple | To describe activities, roles and experiences of service users involved in mental health research |
| Paxton | 2007 | Multiple Countries | Empirical | Health Planning/Service Design/QI | HIV/AIDS | Adult | To examine challenges to GIPA, i.e. HIV-positive people's involvement in AIDS policy making and project design and implementation, in Cambodia, India, and Indonesia |
| Peikes | 2016 | United States of America | Empirical | Health Planning/Service Design/QI | Primary care | Adult | To identify important considerations for primary care practices and other providers considering establishing PFACs, as well as ways to improve established PFACs and areas for future research |
| Pelletier | 2015 | Canada | Empirical | Health Research | Mental health | Adult | To explore the feasibility and acceptability of patient partnership for developing an interactive guide to improve access to primary care providers for chronic diseases management and health promotion among patients with severe mental illness |
| Perfetto | 2015 | United States of America | Review | Other | Drug development | Adult | To provide a summary on what is known to date about the Food and Drug Administration's (FDA's) Patient-Focused Drug Development (PFDD) initiative and describe implications for patients, researchers, payers, and the biopharmaceutical industry |
| Perlmutter | 2015 | United States of America | Review | Health Research | Cancer | Adult | The goal of this article is to define the term advocate, review the role of advocates in cancer research and to describe strategies to effectively engage advocates in cancer research |
| Petit-Zeman | 2010 | United Kingdom | Empirical | Health Research | Multiple conditions | Adult | This article identifies 5 areas where medical research charities involve patients and discusses some of the methods used. |
| Pflugeisen | 2019 | United States of America | Empirical | Health Planning/Service Design/QI | Adolescent and Young Adult Cancer | Multiple | This article describes the formation and first meeting of a community adolescent and young adult oncology council (AYAOC), which was created to promote patient and stakeholder involvement in research and programmatic initiatives within community-based cancer centres. |
| Phelps | 2017 | United Kingdom | Non-Empirical | Health Policy/Governance | N/A | Adolescent | This article presents examples from England of the participation of children with caring responsibilities (young carers) in policy and practice at both local and national levels. |
| Phoenix | 2018 | Canada | Non-Empirical | Health Research | N/A | N/A | We draw on qualitative research perspectives to reflect on these three areas of concern and propose insights into the theory and methods that we believe are useful for engaging patients in research |
| Pickles | 2007 | United Kingdom | Empirical | Health Planning/Service Design/QI | Head & Neck Cancer | Adult | The purpose of this paper is to describe a study which aims to provide an alternative approach to clinical governance. This involves patients in redesigning services based on their actual experiences of health services. This will be of interest to front line health staff and public and patient involvement leads |
| Pii | 2018 | Multiple Countries | Review | Health Research | Cancer | Adult | The aim of this review is to describe the current state of PPI in cancer research. Three central research questions will be explored; a) At which stages of research does PPI take place and which methods are applied? B) What are the stated purposes and outcomes of PPI? and c) What are the stated challenges and recommendations of the PPI process? The findings from this review are discussed in terms of the democratic and research-oriented values of PPi in cancer research |
| Poleshuck | 2015 | United States of America | Empirical | Health Planning/Service Design/QI | Depression (in Women) | Adult | We aimed to use stakeholder input to develop innovative methods for a comparative effectiveness trial to address the needs of socioeconomically disadvantaged women with depression in women's health practices. |
| Pollard | 2014 | United Kingdom | Empirical | Health Research | N/A | Adult | The purpose of this paper is to report learning experiences from involving service users as research partners in two projects that developed and evaluate guidelines for good practice in this regard. The main objective was to evaluate these guidelines |
| Pomey | 2016 | Canada | Non-Empirical | Health Policy/Governance | Chronic illness | Adult | The authors share some of the avenues that have been taken in Quebec to structure patient engagement in a comprehensive way within and across stakeholder and patient populations. |
| Pomey | 2018 | Canada | Non-Empirical | Health Policy/Governance | N/A | N/A | In this article, using examples from patient partnership movements emerging in Quebec, we present best practices to prepare teams to better engage with patient partners and families and show how teams appreciate patients and families engagement. |
| Pomey | 2020 | Canada | Empirical | Health Technology Assessment | Lyme disease | Adult | The objectives of this study were to describe i) the process of trialing different modalities of patient engagement to integrate a diversity of patient perspectives and ii) the learning process of the Lyme project team regarding the integration of patient perspectives |
| Portalupi | 2017 | United States of America | Empirical | Health Research | Multiple conditions | N/A | We describe the process of developing a standing patient and family advisory panel to incorporate this population's voice into research in the USA. |
| Potter | 2010 | United States of America | Empirical | Health Policy/Governance | Children's Mental Health | Adult | This paper examines the experiences of three U.S communities in implementing a service delivery framework for children's mental health, known as community collaboratives. |
| Preston | 2019 | United Kingdom | Non-Empirical | Health Research | Diabetes | Adult | This paper describes just some of the ways our Diabetes Research Group has been developing a PPIE strategy over the last decade. |
| Price | 2017 | Multiple Countries | Review | Health Research | N/A | Adult | This systematic overview of systematic reviews was undertaken to gather research into a single document to identify available evidence and best practice for PPI in the design of clinical trials |
| Quennell | 2001 | United Kingdom | Empirical | Health Policy/Governance | N/A | Adult | The paper focuses on formal and informal involvement of patient groups in NICE structures and appraisals process |
| Quennell | 2003 | United Kingdom | Empirical | Health Policy/Governance | N/A | Adult | This paper examines patient organisations participation in the technology appraisals process of the National Institute for Clinical Excellence (NICE). |
| Rabehariosa | 2003 | France | Empirical | Multiple | Muscular Dystrophy/Neuromuscular Diseases | Pediatric | The aim of this article is to report on analyses and reflection stemming from this research. It starts with a brief presentation of the two classic models of patient organization's engagement in research, proceeds to a case study of the AFM and concludes with a discussion of the partnership model. |
| Rae | 2017 | United Kingdom | Non-Empirical | Multiple | Mental health | Adult | In this commentary, the author comments on PROMISE (Proactive Management of Integrated Services and Environments) and Patient Advisory Groups (PAG) and how individual experiences can influence a patient to lead change. |
| Ramazani | 2020 | United States of America | Empirical | Health Planning/Service Design/QI | pediatric impatient care | Adult | The aim of this study was to assess the perceived impact of including a family advisor as a co-lead on a QI initiative to improve the family-centered timing of routine morning lab work performed on pediatric inpatients through a qualitative theme analysis |
| Rashid | 2017 | United Kingdom | Review | Health Policy/Governance | N/A | Adult | This review seeks to summarise the current practice of PPI in healthcare guidance development and highlight future challenges. |
| Reeve | 2002 | Canada | Empirical | Health research | Mental Health | Adult | The overall purpose of this research was to understand changes in three local community mental health organizations in Waterloo Region, Ontario as they strive to implement an emerging paradigm emphasizing stakeholder participation and empowerment, community support and integration and access to valued resources. |
| Renedo | 2011 | United Kingdom | Empirical | Health Planning/Service Design/QI | N/A | Adult | In this paper, we examine discourses about PPI among healthcare professionals in London, and explore how involvees negotiate these discourses when making sense of themselves as public participants. |
| Renedo | 2015b | United Kingdom | Empirical | Health Planning/Service Design/QI | N/A | Adult | In this paper, we examine what "quality" improvement" means to patients who participate in shaping health care services, and how they use these understandings when attempting to improve quality of care. |
| Renedo | 2015a | United Kingdom | Empirical | Health Planning/Service Design/QI | N/A | Adult | In this article, we examine the ways patients use specific elements of the organizational culture of PPI as resources for their involvement in quality improvement work (i.e., initiatives that use systematic approaches to make changes in service provision to improve patient outcomes and experience) |
| Repper | 2006 | United Kingdom | Non-Empirical | Multiple | Mental health | Adult | Description of a user-focused monitoring approach to involve self-identified survivors or service users in research for mental health services. |
| Rhodes | 2001 | United Kingdom | Empirical | Health Research | Diabetes | Adult | This paper will explore some of the issues raised by patient involvement, focusing specifically on a service users' advisory group established as part of a diabetes service evaluation |
| Robbins | 2016 | United States of America | Non-Empirical | Health Research | N/A | Adult | This commentary, based on our experiences aims to help other research teams to 1) understand how effectively collaborate with stakeholder teams such as patients; 2) anticipate possible challenges; and 3) offer tools for the orientation, training, and integration of patients into a scientific team |
| Rose | 2016 | United Kingdom | Empirical | Health Policy/Governance | Mental health | Adult | To explore mental health service user-led organizations (ULO) in England as they interact with decision makers to bring about change desired by them with a focus on institutional norms behaviours and specialized knowledge impacting service users' relationships with services. |
| Roth | 2011 | United States of America | Non-Empirical | Multiple | N/A | Adult | In this reflection, the author comments on her perspectives working as a patient representative on a few organizations including the California Institute for Regenerative Medicine |
| Roy | 2001 | Canada | Empirical | Multiple | AIDS | Adult | The research had four basic objectives: to describe the current level of participation of PWA within the community-based AIDS movement in Canada; to determine the nature of their involvement; to identify successful strategies used to promote greater and more meaningful involvement of PWA; and to identify barriers PWA were experiencing and to develop strategies on how to bring down these barriers. |
| Sage | 2020 | United States of America | Empirical | Health Research | vascular Ehlers-Danlos Syndrome | Adult | The aim of this paper was to describe the steps and strategies employed by the vEDS Research Collaborative to develop infrastructure and research priorities with engagement from vEDS stakeholders. |
| Sandvin Olsson | 2020 | Multiple Countries | Review | Health Planning/Service Design/QI | Multiple | Adult | The objective of the current scoping review is therefore to investigate how impact of adult patient participation in health service development is described in the literature. |
| Sangill | 2019 | Multiple Countries | Review | Health Research | Mental health | Adult | The aim of this study was to identify the range and scope of empirical research of how mental health service users are involved in collaborative research processes and to summarize this research in dialogue with mental health user-researchers. |
| Saunders | 2011 | Australia | Empirical | Health Research | Multiple conditions | Adult | The goal of this exploratory study was to identify and describe notable cases of consumer involvement in Australia health research to generate insights and concepts, and assist others to develop and build capacity in this area. |
| Sbaih | 2002 | United Kingdom | Empirical | Health Planning/Service Design/QI | Emergency Department | Adult | In this report, the author comments on a method for developing a group of service users to create a partnership approach which would promote collaborative, informed action aimed at delivering, evaluating and developing the emergency service. |
| Schlaudecker | 2021 | United States of America | Non-Empirical | Health Planning/Service Design/QI | N/A | Adult | To describe the transition and success of moving a PFAC to a virtual setting in the light of COVID-19 |
| Schloz | 2017b | Australia | Empirical | Health Policy/Governance | Mental health | Adult | Contemporary mental health policies call for consumers to be involved in decision-making processes within mental health organizations. Some organizations have embraced leadership roles for consumers but research suggests consumers remain disempowered within mental health services. Drawing on service-dominant logic, which emphasizes the co-creation of value of services, the present study provides an overview of consumer leadership within mental health organizations in the Australian Capital Territory. |
| Schloz | 2017a | Australia | Review | Health Policy/Governance | Mental health | Adult | The aim of this systematic review is to synthesize current academic knowledge about mental health service consumers in mental health service leadership. Specifically, this review aims to include all empirical and review articles published in English in peer reviewed journals that explicitly analyse or discuss mental health service consumer leadership of and within mental health organizations. |
| Schloz | 2019 | Australia | Review | Multiple | Mental health | Adult | This review seeks to suggest further research directions In regards to consumer and ally relationships within the mental health sector. |
| Schloz | 2018a | Australia | Empirical | Health Planning/Service Design/QI | N/A | Adult | The aim of this study was to explore power relations between consumers and health professionals engaged in systemic partnerships. |
| Schloz | 2018b | Australia | Empirical | Multiple | Mental health | Adult | The aim of the current study is to better understand how consumers can bring value to partnerships with mental health organizations. |
| Scholz | 2020 | Australia | Empirical | Health Planning/Service Design/QI | Mental Health | Adult | The aim of the current study is to develop a clearer understanding about perceptions of consumer representatives and their ability to influence the systems in which they engage |
| Selig | 2019 | United States of America | Empirical | Health Research | Cancer | Adult | This paper summarizes the PAAC initiative and key learnings as the PAAC model is integrated across the company's oncology pipeline and its leaders share best practices. |
| Sharma | 2018 | United States of America | Empirical | Health Policy/Governance | Multiple conditions | Adult | The aim of this study is to define the roles of patients who participate in governing boards and PACs, comparing and contrasting them as vehicles for patients to engage in practice improvement and clinic leadership. |
| Sharma | 2015 | United States of America | Non-Empirical | Health Planning/Service Design/QI | N/A | Adult | This article draws on our experiences working with patient advisory councils at several different sites. The purpose is to provide guidance on the formation and execution of patient advisory councils in clinical settings. |
| Sharma | 2017 | Multiple Countries | Review | Health Planning/Service Design/QI | N/A | Adult | Our primary aim is to investigate the impact of interventions involving patient advisory councils on clinical care outcomes, patient safety, and patient satisfaction compared to care that doesn't involve patient advisors, for participants at all healthcare settings. |
| Sharma Mahendra | 2020 | United Kingdom | Non-Empirical | Health Research | HIV; Oral Health | Adult | This panel of the 8th World Workshop on Oral Health and Diseases in AIDS considered the role of people living with HIV (PLHIV) to contribute to oral health and HIV research and policy through a process of involvement and empowerment. The panel introduced the concepts of PPI, described the purpose of PPI, reflected upon the logistic and ethical considerations thereof and considered how PPI had been utilised effectively in HIV research and policy change |
| Shea | 2005 | Canada | Non-Empirical | Health Research | Musculoskeletal and Arthritic Diseases | Adult | In this paper, the authors discuss how they have built a partnership with patients to involve them into research activities |
| Sheikhan | 2021 | Canada | Empirical | Multiple | Mental Health | Multiple | This study explores the team's experience of youth and family engagement in the design and development of the YouthCan IMPACT randomized controlled trial and clinical service pathway. |
| Shen | 2016 | Multiple Countries | Review | Health Research | Multiple conditions | Adult | The objectives of this scoping review were to i) synthesize current evidence on engaging parents as co-researchers in health research; ii) identify the potential benefits and challenges of engaging parent co-researchers; and iii) identify gaps in the literature |
| Shklarov | 2017 | Canada | Empirical | Health Research | Chronic Conditions | Adult | Implement and test a new research method and training curriculum to build patient capacity for engagement in health through peer-to-peer research. |
| Skovlund | 2020 | Denmark | Empirical | Health Research | Cancer | Adult | This is an explorative single case study of a Danish, clinical, controlled intervention trial that included patient representatives and a nested intervention fidelity study included herein. |
| Smith | 2009 | United Kingdom | Empirical | Health Research | Multiple conditions | Adult | This paper aims to support the critical development of user involvement in systematic reviews by explaining some of the theoretical, ethical, and practical issues entailed in 'getting ready' for user involvement. |
| Smith | 2000 | United States of America | Non-Empirical | Health Policy/Governance | Mental health | Adult | In this commentary, the author defines the arenas and the types of possible consumer involvement and then comments on the situation in Oregon. |
| Smith | 2014 | United Kingdom | Empirical | Health Research | N/A | Adult | The aim of this study was to explore the views of stakeholders involved in piloting the PIM: the service user and Carer, key personnel in the hosting voluntary sector organisation, relevant members of the KTP team. The emphasis was placed on the experiences of the SUs and Cs of being involved in a context of partnership and working closely with NHS managers and academics. |
| Smith | 2017 | United States of America | Non-Empirical | Health Research | Multiple sclerosis | Adult | In this commentary, the author comments on patients involved as active collaborators in clinical trials and throughout the research and development process. |
| Smith | 2020 | United Kingdom | Empirical | Multiple | Mental Health | Senior/Older Adult | The authors conducted a reflective review of their experiences of running ‘‘ResearchNet’’, a group aimed at putting service users’ perspectives at the heart of service improvement projects, which benefits from and develops its members’ related skills. The authors explore overcoming barriers to service user involvement in research. |
| Solomon | 2017 | United States of America | Empirical | Health research | Cancer | Adult | We describe the process and outcomes of involving patient and physician stakeholders in the design and development of a trial, funded by the Patient-Centered Outcomes Research Institute (PCORI) to enhance oncologists communication skills and their propensity to facilitate productive, meaningful GoC discussions with patients with advanced cancer |
| Souliotis | 2018b | United Kingdom | Empirical | Health Policy/Governance | Cancer | Adult | To examine the degree and impact of cancer patient organization (CPO) participation in healthy policy decision making in EU-28 and to identify their correlates. |
| Souliotis | 2018a | United Kingdom | Empirical | Health Research | Cancer | Adult | In response to this research call, the present study aimed to provide a snapshot of cancer patients' organization (CPO) participation in health policy processes in European Union (EU) -28 countries. |
| South | 2016 | United Kingdom | Empirical | Health Research | N/A | Adult | This paper developed a series of cases studies of PPI to document and share good practice. |
| Speers | 2015 | United Kingdom | Empirical | Health Planning/Service Design/QI | N/A | Adult | This study involved stakeholders (mentors, service users and a lecturer) working together to design, evaluate, and refine a system enabling students to seek feedback from service users. The feedback concerned mental health student interpersonal skills and occurred whilst on practice placement. This research aimed to explore the experiences of those concerned when nine students attempted to learn from rather than about service users. |
| Squire | 2006b | United Kingdom | Empirical | Health Planning/Service Design/QI | Rheumatology | Adult | To provide an overview of the learning from four practical programmes that explore different aspects of patient participation in healthcare provision. |
| Squire | 2006a | United Kingdom | Non-Empirical | Health Planning/Service Design/QI | N/A | Adult | To describe the purpose, establishment, work and achievements of Expert Patients Programme (EPP) of the NHS Modernisation Agency Clinical Governance Development Programme. |
| Staniszewska | 2011 | United Kingdom | Non-Empirical | Health Research | N/A | Adult | In this paper, we argue that a paradigm change toward robust measurement of impact of involvement in research is needed to complete qualitative explorations. We argue that the service users should be collaboratively involved in the conceptualization, theorization and development of instruments to measure PPI impact. |
| Stergiopoulos | 2019 | United States of America | Empirical | Health Research | N/A | Adult | To quantify and define patient-centric initiatives (PCIs) used in clinical research and development; to define evidence-based metrics and performance indicators that determine and measure the impact of return on engagement (ROE) of specific PCIs via biopharmaceutical case studies and real examples of PCI use |
| Stevens | 2003 | United Kingdom | Non-Empirical | Health Research | Cancer | N/A | To identify opportunities for consumers to influence the research process at each stage of the research process; to examine the different types of consumer involvement; to discuss novel ways of identifying and recruiting consumers by one cancer network |
| Stevenson | 2019 | Ireland | Empirical | Health Research | Dementia | Multiple | To describe how a group of individuals with dementia were involved as co-researchers in analysis of extracts from interview data forming part of a multistage study on risk communication in dementia care |
| Stewart | 2015 | United States of America | Empirical | Health Research | N/A | Adult | To describe an enhanced model of community engagement in which a community-linked research infrastructure was developed to involve minorities in research both as participants and as partners engaged in issue selection, study design, and implementation |
| Stewart | 2012 | United Kingdom | Non-Empirical | Health Research | Various | N/A | To argue that research quality and relevance are optimized when patient expertise is integrated with researchers' and policymakers' expertise, and each role acknowledged and valued. This alternative model to the hierarchy places research at the centre and acknowledges different areas of expertise that contribute to and improve research relevance and quality. |
| Stickley | 2009 | United Kingdom | Empirical | Education (Health Professions) | Mental Health | Adult | To describe a model of service user participation in the development of mental health nurse curricula in a UK university |
| Straiton | 2020 | Australia | Non-Empirical | Health Research | COVID-19 | N/A | The purpose of this article is not to reiterate the case for involving consumers in research in general but rather to explore some of the ethical issues COVID-19 has presented to research and how involving consumers may help address these concerns. It also explores opportunities to further support and foster consumer involvement both in future research and in the research process across Australia. |
| Strassle | 2020 | United States of America | Non-Empirical | Multiple | Multiple conditions | Adult | This article presents a new framework for patient engagement by first describing the goals of patient engagement at each stage of the research enterprise and then establishing how to prioritize the types of patient expertise that are needed to achieve these goals. |
| Stuhlfauth | 2019 | Norway | Empirical | Health Research | Rehabilitation | Adult | By bringing together researchers and patient representatives, this study explores and describes both parties' experiences with user involvement in research as they appear through interactions in a focus group |
| Susanti | 2020 | Indonesia | Empirical | Multiple | Psychosis | Adult | To understand service users' and carers' views on the current use and potential applicability of PPI within Indonesian mental health services |
| Switzer | 2019 | Canada | Empirical | Health Research | HIV | Adult | To reflect on ethical, methodological, and pedagogical considerations for designing and facilitating community advisory committees for people living with HIV who use drugs, using Research Rec as a case study |
| Synnot | 2018 | Australia | Empirical | Health Research | Multiple Sclerosis | Adult | Aimed to describe the people, activities and methods of consumer engagement in a complex Australian health research project, and to reflect on the influence this had on the research and people involved, and the enablers and challenges of engagement. |
| Tanner | 2012 | United Kingdom | Empirical | Health Research | Dementia | Senior/Older Adult | The paper discusses the implications for people with dementia of involvement in research as co-researchers |
| Taylor | 2018 | United Kingdom | Empirical | Health Research | Mental health | Adult | To share our jointly developed techniques to ensure the meaningful engagement and contribution of people with lived experience of the criminal justice system (PWLECJS) in research, trial science, intervention theory development and dissemination |
| Taylor | 2006 | United Kingdom | Empirical | Health Research | N/A | Senior/Older Adult | The primary aim of the study reported was to identify any research training, support and information needs of ten UK Older People’s Forums and, following this, to explore how these might be met. |
| Taylor | 2021 | Norway | Empirical | Health Research | Osteo Arthritis | Adult | The article describes this journey looking at formal processes of patient involvement - organizational structure, budget, meetings – and more informal processes such as building relationships and changing researcher perceptions. |
| Telford | 2004a | United Kingdom | Empirical | Health Research | N/A | Adult | To obtain consensus on the principles and indicators of successful consumer involvement in NHS research |
| Telford | 2004b | United Kingdom | Review | Health Research | Mental health | Adult | The aims of this paper were to investigate: how far service user involvement in mental health research appears to have been understood, how far it is happening, reasons why service users get involved in research, and barriers to closer involvement from both service user and researcher perspectives |
| Tempfer | 2011 | Multiple Countries | Review | Multiple | Multiple conditions | Adult | To provide an overview of published data on user participation in Health Care |
| Thompson | 2014 | United Kingdom | Empirical | Health Research | Cancer | Adult | it seems particularly important to understand what motivates some individuals to get (and remain) involved in PPI work whereas others do not, and to understand the potential impacts of PPI on those involved. Furthermore, understanding why people get involved in research and what they stand to gain (or lose) might assist us in maintaining sustainability of approaches and broadening the reach of PPI initiatives to include individuals from seldom-heard groups. In this article we seek to address this gap in the knowledge by providing findings from interviews with patients and carers involved in cancer research settings as PPI participants. |
| Thompson | 2020 | Canada | Empirical | Health Research | Pediatric functional constipation | Pediatric | This study aims to use patient-engagement methods to establish a research collaboration with parents to cocreate a digital knowledge translation tool for parents caring for a child with functional constipation and formally evaluate th e patient engagement processes within this project to build the science of patient engagement in research. |
| Thornton | 2006 | United Kingdom | Non-Empirical | Health research | Breast cancer | Adult | commentary on author's experience with patient advocacy and involvement in breast cancer clinical trials |
| Thornton | 2003 | United Kingdom | Non-Empirical | Multiple | N/A | Adult | This paper offers ‘consumer-led’ reflections by steering group members of a patient-centred research study involving consumer advocates, patients’ associations and patients, throughout the whole study, from pre- to post-study phases. |
| Thornton | 2002 | United Kingdom | Non-Empirical | Health Research | cancer | Adult | This paper offers a brief outline of recent consumer involvement in research and practical suggestions. |
| Tierney | 2016 | International | Review | Multiple | N/A | Adult | The aim of this review was to critically integrate the conditions for the implementation of SUI in both primary care research and health service development projects to make recommendations that will enhance chances of its normalization |
| Tremblay | 2020 | Canada | Empirical | Health Research | Diabetes | Adult | This article reports on the findings of an evaluation conducted at the end of the project to garner lessons and identify strategies for engaging Indigenous patient partners in patient oriented research. |
| Trivedi | 2002 | United Kingdom | Empirical | Health Research | Mental Health | Adult | This review describes our experience of working together for the first time with user-researchers on a study investigating the effects of group medication education sessions on in-patients in our local psychiatric intensive care unit (PICU) |
| Troya | 2019 | United Kingdom | Empirical | Health Research | Self-harm in older adults | Senior/Older Adult | The aim of this paper was to critically reflect on the process, potential impact and identify challenges/opportunities in involving robust PPIE in a doctoral research, including a SR and qualitative study. |
| Truman | 2002 | United Kingdom | Empirical | Health Planning/Service Design/QI | Mental health | Adult | The authors explore ways in which the reality of user involvement is subject to a range of configurations within health services. The paper describes a piece of qualitative research that was undertaken within a participatory framework to explore the nature of user involvement within the facility. |
| Tullo | 2015 | United Kingdom | Empirical | Health Research | Ageing | Senior/Older Adult | To survey current levels of PPI in biomedical and clinical research relating to ageing at one institution. To compare and contrast the views of academics and the public about PPI relating to research about ageing. |
| Tuttle | 2021 | United States of America | Empirical | Health Research | Chronic Kidney Disease | Adult | The purpose of this report is to describe patient and community engagement and the value they bring to the Kidney Precision Medicine Project (KPMP). |
| Vale | 2018 | United Kingdom | Empirical | Health Research | N/A | Adult | We aimed to highlight inconsistencies between existing guidance on participant involvement in PPI strategies for clinical trials, to provide guidance and influence researchers to give due consideration to this potentially novel aspect of PPI for future trials. |
| Vale | 2012 | United Kingdom | Empirical | Health Research | cervical cancer | Adult | This paper describes the involvement of women who had experienced treatment for cervical cancer in the systematic review, to inform the discussion about the treatments involved and, in particular, how side effects might impact on women’s day to-day lives post treatment. In addition to involving patients in the systematic review process, we also aimed to evaluate involvement with the aim of informing the practice of patient involvement in future systematic reviews conducted by our group and others. |
| van der Ham | 2016 | Netherlands | Empirical | Other | Mental health | Adult | The aim of this study was to gain better insight into the quality of patient participation in the development of clinical practice guidelines and to contribute to approaches for the monitoring and evaluation of such initiatives. In addition, we explore the potential of a dialogue-based approach for reconciliation of preferences of patients and professionals in the guideline development processes. |
| van Draanen | 2013 | Canada | Empirical | Health Research | Mental health | Multiple | The specific objectives of this study included: (a) to describe the implementation of the People with Lived Experience Caucus and barriers and facilitators to implementation; and (b) to investigate the factors that facilitated meaningful inclusion of Caucus representatives in research and service planning––from the perspectives of both Caucus members and project stakeholders. |
| van Staa | 2010 | Netherlands | Empirical | Health Research | Chronic conditions (dermatologic disorders, blood disorders, neuromuscular diseases, renal failure and diabetes mellitus) | Adolescent | To evaluate the feasibility, benefits and limitations of a participatory research (PR) project involving chronically ill adolescents as co-researchers |
| van Wersch | 2001 | United Kingdom | Empirical | Other | Multiple (asthma, angina, myocardial infarction) | Adult | This paper describes and discusses our experiences with different methods of involving consumers to inform guideline development as a series of case studies and, from this basis, suggests how these methods could be developed. |
| Vat | 2017 | Multiple Countries | Empirical | Health Research | N/A | Adult | The goal of this study was to describe ways that patient partners have been recruited by researchers and patient engagement leads |
| Vat | 2021 | Multiple Countries | Empirical | Health Research | N/A | Adult | The objective of this project was to co-design a monitoring and evaluation framework for patient engagement in medicine development, with metrics, to demonstrate impact and enhance learning. |
| Vat | 2020 | Canada | Empirical | Health Research | N/A | Adult | In this pilot evaluation study, we aimed to 1) evaluate patient engagement in health research projects in Newfoundland and Labrador, Canada, and 2) learn more about how to best monitor and evaluate patient engagement. |
| Vogsen | 2020 | Denmark | Empirical | Health Research | Breast Cancer | Adult | Our aim was to evaluate the impact on patient recruitment and retention of having patients as research partners in a clinical study of breast cancer. We also report our experience regarding researchers’ attitudes towards involving patients as partners in the research process. |
| Wale | 2020 | Multiple Countries | Empirical | Health Technology Assessment | Crohn's Disease; Cancer | Adult | We explored how written and oral patient involvement in two HTAs was reported on in publicly available final recommendations and discussion summaries of appraisal committees from three HTA bodies. We aimed to gain insights into how patient input was utilized by appraisal committees to better understand the goals of patient involvement and how these are being achieved. |
| Walker | 2018 | United Kingdom | Empirical | Health Research | Mental Health | Adult | This report outlines the process of exploring a proposed research project with a group of service users. |
| Wall | 2004 | United Kingdom | Empirical | Health Planning/Service Design/QI | N/A | Adult | This paper examines how patients and carers are involved in the work of the CGST, specifically as patient consultants in the CGST's Performance Development Team (PDT). |
| Warren | 2018 | United States of America | Non-Empirical | Health Research | N/A | N/A | We describe and reflect on our patient stakeholder groups, engagement framework, experiences, and lessons learned in engaging patients in research, from generating proposal ideas to disseminating findings. |
| Warren | 2020 | Canada | Empirical | Health Research | N/A | Adult | In this paper, we describe 2 PACs from Western and Eastern Canada, explaining their governance structure, primary functions, creation and composition, and specific activities undertaken by members (e.g., review research funding applications and serve as project co-investigators). |
| Weeks | 2017 | Multiple Countries | Empirical | Health Technology Assessment | N/A | N/A | We conducted a survey of international HTA agencies to address the need to better understand whether and how HTA programs are evaluating their PPI strategies and with what results, as well as perceived facilitators and barriers to evaluation. |
| Wennerstrom | 2018 | United States of America | Empirical | Other | N/A | Adult | In this article, we highlight three current examples of P2P-funded projects – their origins, development, and prospects. We identify how these three projects use CPPR and have collaborated with and contributed to a two-way learning and knowledge exchange among community and academic partners of the CPPRN. |
| Westfall | 2006 | United States of America | Empirical | Health Research | N/A | Adult | The purpose of this article is to describe the use of community-based participatory research methods among practice-based research networks (PBRNs) in the United States. |
| Wheeler | 2020 | Canada | Empirical | Health Planning/Service Design/QI | Cancer | Adult | To understand the barriers and facilitators PFAs experience when they are engaged in health-care system planning and provide recommendations for future engagement. |
| Wilson | 2018 | United States of America | Non-Empirical | Health Planning/Service Design/QI | N/A | N/A | The purpose of this paper is to propose a framework for engaging patients and patient partners in the critical step of selecting and/or designing clinical outcome assessments (COAs) for use as endpoints to evaluate the benefit of medical products. |
| Wilson | 2010 | United Kingdom | Empirical | Health Research | Mental Health | Adult | This paper reports on the rationale, purpose and benefits of having a service user and carer-led research group and draws on relevant evidence to support this initiative in Wales. |
| Young | 2020 | United States of America | Empirical | Health Research | Diabetes | Adult | The purpose of this paper is to discuss engagement activities, roles and responsibilities, and value and contributions of a Patient Advisory Board (PAB), as well as lessons learned. |
| Zibrowski | 2020 | Canada | Review | Health Research | N/A | Adult | This protocol will guide the development of a rapid realist review, to locate existing patient-oriented research studies that have been published in scientific journals or have been communicated by government sponsored initiatives in Canada and internationally. |

Reference List

Abbass-Dick, J., Brolly, M., Huizinga, J., Newport, A., Xie, F. L., George, S., & Sterken, E. (2018). Designing an eHealth Breastfeeding Resource With Indigenous Families Using a Participatory Design. *Journal of Transcultural Nursing, 29*(5), 480-488. doi:10.1177/1043659617731818

Abelson, J. (2018). Patient engagement in health technology assessment: what constitutes 'meaningful' and how we might get there. *Journal of Health Services & Research Policy, 23*(2), 69-71. doi:https://dx.doi.org/10.1177/1355819618756936

Abelson, J., Giacomini, M., Lehoux, P., & Gauvin, F. P. (2007). Bringing 'the public' into health technology assessment and coverage policy decisions: from principles to practice. *Health Policy, 82*(1), 37-50.

Abelson, J., Wagner, F., DeJean, D., Boesveld, S., Gauvin, F. P., Bean, S., . . . Lavis, J. (2016). Public and Patient Involvement in Health Technology Assessment: A Framework for Action. *International Journal of Technology Assessment in Health Care, 32*(4), 256-264.

Abma, T. A. (2005). Patient participation in health research: research with and for people with spinal cord injuries. *Qualitative Health Research, 15*(10), 1310-1328.

Abma, T. A. (2006). Patients as partners in a health research agenda setting: the feasibility of a participatory methodology. *Evaluation & the Health Professions, 29*(4), 424-439.

Abma, T. A., Nierse, C. J., & Widdershoven, G. A. (2009). Patients as partners in responsive research: methodological notions for collaborations in mixed research teams. *Qualitative Health Research, 19*(3), 401-415. doi:https://dx.doi.org/10.1177/1049732309331869

Abrams, R., Park, S., Wong, G., Rastogi, J., Boylan, A. M., Tierney, S., . . . Roberts, N. (2021). Lost in reviews: Looking for the involvement of stakeholders, patients, public and other non-researcher contributors in realist reviews. *Research Synthesis Methods, 12*(2), 239-247. doi:10.1002/jrsm.1459

Absolom, K., Holch, P., Woroncow, B., Wright, E. P., & Velikova, G. (2015). Beyond lip service and box ticking: how effective patient engagement is integral to the development and delivery of patient-reported outcomes. *Quality of Life Research, 24*(5), 1077-1085. doi:https://dx.doi.org/10.1007/s11136-014-0909-z

Acri, M., Olin, S. S., Burton, G., Herman, R. J., & Hoagwood, K. E. (2014). Innovations in the Identification and Referral of Mothers at Risk for Depression: Development of a Peer-to-Peer Model. *Journal of Child & Family Studies, 23*(5), 837-843.

Adams, G., Green, A., Towe, S., & Huett, A. (2013). Bereaved caregivers as educators in pediatric palliative care: their experiences and impact. *Journal of Palliative Medicine, 16*(6), 609-615. doi:https://dx.doi.org/10.1089/jpm.2012.0475

Addario, B., Geissler, J., Horn, M. K., Krebs, L. U., Maskens, D., Oliver, K., . . . Willmarth, N. (2019). Including the patient voice in the development and implementation of patient-reported outcomes in cancer clinical trials. *Health Expectations, 13*, 13. doi:https://dx.doi.org/10.1111/hex.12997

Adler, J. L. (2017). "The Service I Rendered Was Just as True": African American Soldiers and Veterans as Activist Patients. *American Journal of Public Health, 107*(5), 675-683. doi:https://dx.doi.org/10.2105/AJPH.2017.303688

Aggarwal, R., & Brissett, J. (2011). Parents and young patients as partners in care. *Health Progress, 92*(3), 24-27.

Aguiar, M., Harrison, M., Munro, S., Burch, T., Kaal, K. J., Hudson, M., . . . Laba, T. L. (2020). Designing Discrete Choice Experiments Using a Patient-Oriented Approach. *The Patient: Patient Centered Outcomes Research, 17*, 17. doi:https://dx.doi.org/10.1007/s40271-020-00431-w

Ahc, M. (2018). Patient and Family Councils Make a Difference. *Hospital Case Management, 26*(11), 141-152. Retrieved from http://libaccess.mcmaster.ca/login?url=http://search.ebscohost.com/login.aspx?direct=true&db=cin20&AN=132208380&site=ehost-live&scope=site

Ahc, M. (2019). Revenue Cycle Needs Feedback From Patients and Family Advisors, Too. *Hospital Access Management, 38*(12), N.PAG-N.PAG. Retrieved from http://libaccess.mcmaster.ca/login?url=http://search.ebscohost.com/login.aspx?direct=true&db=cin20&AN=139723580&site=ehost-live&scope=site

Al Hamarneh, Y. N., Rosenberg-Yunger, Z., Saxena, A., Waite, N. M., Dolovich, L., & Tsuyuki, R. T. (2020). Patient-oriented pharmacy practice research: Why should we care? *Canadian Pharmacists Journal, 153*(3), 133-136. doi:10.1177/1715163520909122

Alderson, H., Brown, R., Smart, D., Lingam, R., & Dovey-Pearce, G. (2019). 'You've come to children that are in care and given us the opportunity to get our voices heard': The journey of looked after children and researchers in developing a Patient and Public Involvement group. *Health Expectations, 22*(4), 657-665. doi:10.1111/hex.12904

Alidina, S., Martelli, P. F., Singer, S. J., & Aveling, E.-L. (2021). Optimizing patient partnership in primary care improvement: A qualitative study. *Health Care Management Review, 46*(2), 123-134. doi:10.1097/HMR.0000000000000250

Allarakhia, M. (2015). Exploring open innovation with a patient focus in drug discovery: an evolving paradigm of patient engagement. *Expert Opinion on Drug Discovery, 10*(6), 571-578. doi:https://dx.doi.org/10.1517/17460441.2015.1037271

Amann, J., Brach, M., & Rubinelli, S. (2018). How healthcare professionals experience patient participation in designing healthcare services and products. A qualitative study in the field of spinal cord injury in Switzerland. *Patient Education and Counseling, 101*(8), 1452-1459. doi:10.1016/j.pec.2018.03.011

Ambrosini, A., Quinlivan, R., Sansone, V. A., Meijer, I., Schrijvers, G., Tibben, A., . . . th, E. W. S. G. (2019). "Be an ambassador for change that you would like to see": a call to action to all stakeholders for co-creation in healthcare and medical research to improve quality of life of people with a neuromuscular disease. *Orphanet Journal Of Rare Diseases, 14*, 12. doi:10.1186/s13023-019-1103-8

Amirav, I., & Vandall-Walker, V. (2017). Patient and Researcher Engagement in Health Research: A Parent's Perspective. *Pediatrics, 140*(3), 1-4. doi:10.1542/peds.2016-4127

Anderson, A., Benger, J., & Getz, K. (2019). Using Patient Advisory Boards to Solicit Input Into Clinical Trial Design and Execution. *Clinical Therapeutics, 41*(8), 1408-1413. doi:https://dx.doi.org/10.1016/j.clinthera.2019.06.006

Anderson, A., & Getz, K. A. (2018). Insights and Best Practices for Planning and Implementing Patient Advisory Boards. *Therapeutic Innovation & Regulatory Science, 52*(4), 469-473. doi:https://dx.doi.org/10.1177/2168479017720475

Anderson, E. S., Ford, J., & Thorpe, L. (2019). Perspectives on patients and carers in leading teaching roles in interprofessional education. *Journal of Interprofessional Care, 33*(2), 216-225. doi:https://dx.doi.org/10.1080/13561820.2018.1531834

Anderson, M., & McCleary, K. K. (2015). From passengers to co-pilots: Patient roles expand. *Science Translational Medicine, 7*(291), 291fs225. doi:https://dx.doi.org/10.1126/scitranslmed.aac6023

Anderson, N. N., Baker, G. R., Moody, L., Scane, K., Urquhart, R., Wodchis, W. P., & Gagliardi, A. R. (2021). Approaches to optimize patient and family engagement in hospital planning and improvement: Qualitative interviews. *Health Expectations, 24*, 24. doi:https://dx.doi.org/10.1111/hex.13239

Andersson, A.-C., & Olheden, A. (2012). Patient participation in quality improvement: managers' opinions of patients as resources. *Journal of Clinical Nursing (John Wiley & Sons, Inc.), 21*(23-24), 3590-3593. doi:10.1111/j.1365-2702.2012.04254.x

Anderst, A., Conroy, K., Fairbrother, G., Hallam, L., McPhail, A., & Taylor, V. (2020). Engaging consumers in health research: a narrative review. *Australian Health Review, 44*(5), 806-813. doi:10.1071/AH19202

Anonymous. (2015). Issue Brief: Patient and Family Engagement: A Partnership for Culture Change: A Report of the NCIOM Task Force on Patient and Family Engagement. *North Carolina Medical Journal, 76*(3), 197-200. doi:https://dx.doi.org/10.18043/ncm.76.3.197

Arain, M., Pyne, S., Thornton, N., Palmer, S., & Sharma, R. A. (2015). Consumer involvement in cancer research: example from a Cancer Network. *Health Expectations, 18*(5), 1530-1542. doi:https://dx.doi.org/10.1111/hex.12143

Arblaster, K., Mackenzie, L., Matthews, L., Willis, K., Gill, K., Hanlon, P., & Laidler, R. (2018). Learning from consumers: An eDelphi study of Australian mental health consumers' priorities for recovery-oriented curricula. *Australian Occupational Therapy Journal, 65*(6), 586-597. doi:https://dx.doi.org/10.1111/1440-1630.12518

Archambault, P. M., McGavin, C., Dainty, K. N., McLeod, S. L., Vaillancourt, C., Lee, J. S., . . . Boivin, A. (2018). Recommendations for patient engagement in patient-oriented emergency medicine research. *CJEM Canadian Journal of Emergency Medical Care, 20*(3), 435-442. doi:https://dx.doi.org/10.1017/cem.2018.370

Arkind, J., Likumahuwa-Ackman, S., Warren, N., Dickerson, K., Robbins, L., Norman, K., & DeVoe, J. E. (2015). Lessons Learned from Developing a Patient Engagement Panel: An OCHIN Report. *Journal of the American Board of Family Medicine: JABFM, 28*(5), 632-638. doi:https://dx.doi.org/10.3122/jabfm.2015.05.150009

Armstrong, M. I., Evans, M. E., & Wood, V. (2000). The development of a state policy on families as allies. *Journal of Emotional and Behavioral Disorders, 8*(4), 240-248. doi:10.1177/106342660000800404

Armstrong, M. J., Mullins, C. D., Gronseth, G. S., & Gagliardi, A. R. (2017). Recommendations for patient engagement in guideline development panels: A qualitative focus group study of guideline-naive patients. *Plos One, 12*(3), 16. doi:10.1371/journal.pone.0174329

Armstrong, N., Herbert, G., Aveling, E. L., Dixon-Woods, M., & Martin, G. (2013). Optimizing patient involvement in quality improvement. *Health Expectations, 16*(3), e36-47. doi:https://dx.doi.org/10.1111/hex.12039

Arnstein, L., Wadsworth, A. C., Yamamoto, B. A., Stephens, R., Sehmi, K., Jones, R., . . . Woolley, K. L. (2020). Patient involvement in preparing health research peer-reviewed publications or results summaries: a systematic review and evidence-based recommendations. *Research Involvement & Engagement, 6*, 34. doi:https://dx.doi.org/10.1186/s40900-020-00190-w

Attree, P., Morris, S., Payne, S., Vaughan, S., & Hinder, S. (2011). Exploring the influence of service user involvement on health and social care services for cancer. *Health Expectations, 14*(1), 48-58. doi:10.1111/j.1369-7625.2010.00620.x

Baart, I., & Abma, T. A. (2011). Patient participation in fundamental psychiatric genomics research: a Dutch case study. *Health Expectations, 14*(3), 240-249. doi:10.1111/j.1369-7625.2010.00634.x

Bailey, S., Boddy, K., Briscoe, S., & Morris, C. (2015). Involving disabled children and young people as partners in research: a systematic review. *Child: Care, Health & Development, 41*(4), 505-514. doi:https://dx.doi.org/10.1111/cch.12197

Baines, R. L., & Regan de Bere, S. (2018). Optimizing patient and public involvement (PPI): Identifying its "essential" and "desirable" principles using a systematic review and modified Delphi methodology. *Health Expectations, 21*(1), 327-335. doi:https://dx.doi.org/10.1111/hex.12618

Baker, G. R., Fancott, C., Judd, M., & O'Connor, P. (2016). Expanding patient engagement in quality improvement and health system redesign: Three Canadian case studies. *Healthcare Management Forum, 29*(5), 176-182. doi:https://dx.doi.org/10.1177/0840470416645601

Baldwin, J. N., Napier, S., Neville, S., & Wright-St Clair, V. A. (2018). Impacts of older people's patient and public involvement in health and social care research: a systematic review. *Age & Ageing, 47*(6), 801-809. doi:https://dx.doi.org/10.1093/ageing/afy092

Banerjee, D., Lowe-Jones, R., Damster, S., Thomas, N., Scholes-Robertson, N., Tong, A., . . . Group, I.-A. P.-E. i. C. T. (2020). International perspectives on patient involvement in clinical trials in nephrology. *Kidney International, 98*(3), 566-571. doi:https://dx.doi.org/10.1016/j.kint.2020.06.023

Banfield, M., Randall, R., O'Brien, M., Hope, S., Gulliver, A., Forbes, O., . . . Griffiths, K. (2018). Lived experience researchers partnering with consumers and carers to improve mental health research: Reflections from an Australian initiative. *International Journal of Mental Health Nursing, 27*(4), 1219-1229. doi:https://dx.doi.org/10.1111/inm.12482

Banner, D., Bains, M., Carroll, S., Kandola, D. K., Rolfe, D. E., Wong, C., & Graham, I. D. (2019). Patient and Public Engagement in Integrated Knowledge Translation Research: Are we there yet? *Research Involvement & Engagement, 5*, 8. doi:https://dx.doi.org/10.1186/s40900-019-0139-1

Bar, S., Grant, K., Asuri, S., & Holms, S. (2018). British Columbia Ministry of Health Patients as Partners: A transformational approach. *Healthcare Management Forum, 31*(2), 51-56. doi:https://dx.doi.org/10.1177/0840470417744569

Barger, S., Sullivan, S. D., Bell-Brown, A., Bott, B., Ciccarella, A. M., Golenski, J., . . . Ramsey, S. D. (2019). Effective stakeholder engagement: design and implementation of a clinical trial (SWOG S1415CD) to improve cancer care. *BMC Medical Research Methodology, 19*(1), 119. doi:https://dx.doi.org/10.1186/s12874-019-0764-2

Barker, J., Moule, P., Evans, D., Phillips, W., & Leggett, N. (2020). Developing a typology of the roles public contributors undertake to establish legitimacy: a longitudinal case study of patient and public involvement in a health network. *BMJ Open, 10*(5), e033370. doi:https://dx.doi.org/10.1136/bmjopen-2019-033370

Barnieh, L., Jun, M., Laupacis, A., Manns, B., & Hemmelgarn, B. (2015). Determining research priorities through partnership with patients: an overview. *Seminars in Dialysis, 28*(2), 141-146. doi:https://dx.doi.org/10.1111/sdi.12325

Bartlett, S. J., Barnes, T., & McIvor, R. A. (2014). Integrating patients into meaningful real-world research. *Annals of the American Thoracic Society, 11 Suppl 2*, S112-117. doi:https://dx.doi.org/10.1513/AnnalsATS.201309-327RM

Bate, J., Ranasinghe, N., Ling, R., Preston, J., Nightingale, R., & Denegri, S. (2016). Public and patient involvement in paediatric research. *Archives of Disease in Childhood Education & Practice, 101*(3), 158-161. doi:https://dx.doi.org/10.1136/archdischild-2015-309500

Bate, P., & Robert, G. (2006). Experience-based design: from redesigning the system around the patient to co-designing services with the patient. *Quality & Safety in Health Care, 15*(5), 307-310.

Batten, L. M., Bhattacharya, I. S., Moretti, L., Haviland, J. S., Emson, M. A., Miller, S. E., . . . Bliss, J. M. (2018). Patient advocate involvement in the design and conduct of breast cancer clinical trials requiring the collection of multiple biopsies. *Research Involvement & Engagement, 4*, 22. doi:https://dx.doi.org/10.1186/s40900-018-0108-0

Baumann, L. A., & Brutt, A. L. (2021). Public and patient involvement (PPI) in health policy decisionmaking on the health system-level: protocol for a systematic scoping review. *BMJ Open, 11*(5), e043650. doi:https://dx.doi.org/10.1136/bmjopen-2020-043650

Bayliss, K., Starling, B., Raza, K., Johansson, E. C., Zabalan, C., Moore, S., . . . Stack, R. (2016). Patient involvement in a qualitative meta-synthesis: lessons learnt. *Research Involvement & Engagement, 2*, 18. doi:https://dx.doi.org/10.1186/s40900-016-0032-0

Bedlack, R., Pogemiller, A., Shefner, J., Cudkowicz, M., & Heiman-Patterson, T. (2020). ALS clinical research learning institutes (ALS-CRLI): empowering people with ALS to be research ambassadors. *Amyotrophic Lateral sclerosis & Frontotemporal Degeneration, 21*(3-4), 216-221. doi:https://dx.doi.org/10.1080/21678421.2019.1690519

Bedwell, C., & Lavender, T. (2020). Giving patients a voice: implementing patient and public involvement to strengthen research in sub-Saharan Africa. *Journal of Epidemiology & Community Health, 74*(4), 307-310. doi:https://dx.doi.org/10.1136/jech-2019-212525

Beier, K., Schweda, M., & Schicktanz, S. (2019). Taking patient involvement seriously: a critical ethical analysis of participatory approaches in data-intensive medical research. *BMC Medical Informatics & Decision Making, 19*(1), 90. doi:https://dx.doi.org/10.1186/s12911-019-0799-7

Beighton, C., Victor, C., Carey, I. M., Hosking, F., DeWilde, S., Cook, D. G., . . . Harris, T. (2019). 'I'm sure we made it a better study...': Experiences of adults with intellectual disabilities and parent carers of patient and public involvement in a health research study. *Journal of Intellectual Disabilities, 23*(1), 78-96. doi:https://dx.doi.org/10.1177/1744629517723485

Belisle-Pipon, J. C., Rouleau, G., & Birko, S. (2018). Early-career researchers' views on ethical dimensions of patient engagement in research. *BMC Medical Ethics, 19*(1), 21. doi:https://dx.doi.org/10.1186/s12910-018-0260-y

Bell, T., Vat, L. E., McGavin, C., Keller, M., Getchell, L., Rychtera, A., & Fernandez, N. (2019). Co-building a patient-oriented research curriculum in Canada. *Research Involvement & Engagement, 5*, 7. doi:https://dx.doi.org/10.1186/s40900-019-0141-7

Benbow, S. M. (2012). Patient and carer participation in old age psychiatry in England. Part II: models of participation. *International Psychogeriatrics, 24*(2), 185-196. doi:10.1017/s1041610211001876

Benbow, S. M., Taylor, L., Mustafa, N., & Morgan, K. (2011). DESIGN, DELIVERY AND EVALUATION OF TEACHING BY SERVICE USERS AND CARERS. *Educational Gerontology, 37*(7), 621-633. doi:10.1080/03601277.2011.559849

Bench, S., O'Shea, A., & Boaz, A. (2020). Patient and Family Member Experiences in Critical Care Research and Quality Improvement Projects. *Nursing Research, 69*(5), 367-375. doi:10.1097/NNR.0000000000000443

Beneciuk, J. M., Verstandig, D., Taylor, C., Scott, D., Levin, J., Osborne, R., . . . McGarrie, L. (2020). Musculoskeletal pain stakeholder engagement and partnership development: determining patient-centered research priorities. *Research Involvement & Engagement, 6*, 28. doi:https://dx.doi.org/10.1186/s40900-020-00192-8

Beresford, P. (2007). User involvement, research and health inequalities: developing new directions. *Health & Social Care in the Community, 15*(4), 306-312.

Berg, R. C., Gamst, A., Said, M., Aas, K. B., Songe, S. H., Fangen, K., & Rysstad, O. (2015). True User Involvement by People Living With HIV is Possible: Description of a User-driven HIV Clinic in Norway. *Journal of the Association of Nurses in AIDS Care, 26*(6), 732-742. doi:https://dx.doi.org/10.1016/j.jana.2015.07.002

Bergerum, C., Engstrom, A. K., Thor, J., & Wolmesjo, M. (2020). Patient involvement in quality improvement - a 'tug of war' or a dialogue in a learning process to improve healthcare? *BMC Health Services Research, 20*(1), 1115. doi:https://dx.doi.org/10.1186/s12913-020-05970-4

Bergerum, C., Thor, J., Josefsson, K., & Wolmesjo, M. (2019). How might patient involvement in healthcare quality improvement efforts work-A realist literature review. *Health Expectations, 22*(5), 952-964. doi:https://dx.doi.org/10.1111/hex.12900

Bergsten, U., Andrey, A. M., Bottner, L., Nylander, M., Persson, G., Petersson, E., & Bergman, S. (2014). Patient-initiated research in rheumatic diseases in Sweden--dignity, identity and quality of life in focus when patients set the research agenda. *Musculoskeletal Care, 12*(3), 194-197. doi:https://dx.doi.org/10.1002/msc.1073

Bernstein, E., Getchell, L., & Harwood, L. (2019). Partnering with Patients, Families, and Caregivers in Nephrology Nursing Research. *Nephrology Nursing Journal: Journal of the American Nephrology Nurses' Association, 46*(3), 340-343.

Bertrand, D. P., Minguet, G., Gagnayre, R., & Lombrail, P. (2018). Lessons from patient and parent involvement (P&PI) in a quality improvement program in cystic fibrosis care in France. *Orphanet Journal Of Rare Diseases, 13*, 18. doi:10.1186/s13023-017-0751-9

Bhati, D. K., Fitzgerald, M., Kendall, C., & Dahrouge, S. (2020). Patients' engagement in primary care research: a case study in a Canadian context. *Research Involvement & Engagement, 6*(1), 65. doi:https://dx.doi.org/10.1186/s40900-020-00238-x

Bignami, F., Kent, A. J., Lipucci di Paola, M., & Meade, N. (2011). Participation of patients in the development of advanced therapy medicinal products. *Bundesgesundheitsblatt, Gesundheitsforschung, Gesundheitsschutz, 54*(7), 839-842. doi:https://dx.doi.org/10.1007/s00103-011-1306-1

Birch, R., Simons, G., Wahamaa, H., McGrath, C. M., Johansson, E. C., Skingle, D., . . . Falahee, M. (2020). Development and formative evaluation of patient research partner involvement in a multi-disciplinary European translational research project. *Research Involvement & Engagement, 6*, 6. doi:https://dx.doi.org/10.1186/s40900-020-0178-7

Bird, M., McGillion, M., Chambers, E. M., Dix, J., Fajardo, C. J., Gilmour, M., . . . Carter, N. (2021). A generative co-design framework for healthcare innovation: development and application of an end-user engagement framework. *Research Involvement & Engagement, 7*(1), 12. doi:https://dx.doi.org/10.1186/s40900-021-00252-7

Bird, M., Ouellette, C., Whitmore, C., Li, L., Nair, K., McGillion, M. H., . . . Carroll, S. L. (2020). Preparing for patient partnership: A scoping review of patient partner engagement and evaluation in research. *Health Expectations, 23*(3), 523-539. doi:https://dx.doi.org/10.1111/hex.13040

Birkeland, S., Pedersen, S. S., Haakonsson, A. K., Barry, M. J., & Rottmann, N. (2020). Men's view on participation in decisions about prostate-specific antigen (PSA) screening: patient and public involvement in development of a survey. *Bmc Medical Informatics and Decision Making, 20*(1). doi:10.1186/s12911-020-1077-4

Bissell, P., Thompson, J., & Gibson, B. (2018). Exploring Difference or Just Watching the Experts at Work? Interrogating Patient and Public Involvement (PPI) in a Cancer Research Setting Using the Work of Jurgen Habermas. *Sociology-the Journal of the British Sociological Association, 52*(6), 1200-1216. doi:10.1177/0038038517749781

Boaz, A., Robert, G., Locock, L., Sturmey, G., Gager, M., Vougioukalou, S., . . . Fielden, J. (2016). What patients do and their impact on implementation. *Journal of Health Organization & Management, 30*(2), 258-278. doi:https://dx.doi.org/10.1108/JHOM-02-2015-0027

Bombard, Y., Baker, G. R., Orlando, E., Fancott, C., Bhatia, P., Casalino, S., . . . Pomey, M. P. (2018). Engaging patients to improve quality of care: a systematic review. *Implementation Science, 13*(1), 98. doi:https://dx.doi.org/10.1186/s13012-018-0784-z

Bookout, M. L., Staffileno, B. A., & Budzinsky, C. M. (2016). Partnering With a Patient and Family Advisory Council to Improve Patient Care Experiences With Pain Management. *Journal of Nursing Administration, 46*(4), 181-186. doi:https://dx.doi.org/10.1097/NNA.0000000000000328

Boote, J., Telford, R., & Cooper, C. (2002). Consumer involvement in health research: a review and research agenda. *Health Policy, 61*(2), 213-236.

Borup, G., Bach, K. F., Schmiegelow, M., Wallach-Kildemoes, H., Bjerrum, O. J., & Westergaard, N. (2016). A Paradigm Shift Towards Patient Involvement in Medicines Development and Regulatory Science. *Therapeutic Innovation & Regulatory Science, 50*(3), 304-311. doi:10.1177/2168479015622668

Boudes, M., Robinson, P., Bertelsen, N., Brooke, N., Hoos, A., Boutin, M., . . . Sargeant, I. (2018). What do stakeholders expect from patient engagement: Are these expectations being met? *Health Expectations, 21*(6), 1035-1045. doi:https://dx.doi.org/10.1111/hex.12797

Bourke, L. (2002). Participatory research in breast cancer: a case study in regional Victoria. *Contemporary Nurse, 12*(3), 246-252.

Boyer, A. P., Fair, A. M., Joosten, Y. A., Dolor, R. J., Williams, N. A., Sherden, L., . . . Wilkins, C. H. (2018). A Multilevel Approach to Stakeholder Engagement in the Formulation of a Clinical Data Research Network. *Medical Care, 56 Suppl 10 Suppl 1*, S22-S26. doi:https://dx.doi.org/10.1097/MLR.0000000000000778

Brady, L.-M., Templeton, L., Toner, P., Watson, J., Evans, D., Percy-Smith, B., & Copello, A. (2018). Involving young people in drug and alcohol research. *Drugs & Alcohol Today, 18*(1), 28-38. doi:10.1108/DAT-08-2017-0039

Breault, L. J., Rittenbach, K., Hartle, K., Babins-Wagner, R., de Beaudrap, C., Jasaui, Y., . . . Mason-Lai, P. (2018). People with lived experience (PWLE) of depression: describing and reflecting on an explicit patient engagement process within depression research priority setting in Alberta, Canada. *Research Involvement & Engagement, 4*, 37. doi:https://dx.doi.org/10.1186/s40900-018-0115-1

Brett, J., Staniszewska, S., Mockford, C., Herron-Marx, S., Hughes, J., Tysall, C., & Suleman, R. (2014). A systematic review of the impact of patient and public involvement on service users, researchers and communities. *The Patient: Patient-Centered Outcomes Research, 7*(4), 387-395. doi:https://dx.doi.org/10.1007/s40271-014-0065-0

Bridges, J. F., & Jones, C. (2007). Patient-based health technology assessment: a vision of the future. *International Journal of Technology Assessment in Health Care, 23*(1), 30-35.

Broer, T., Nieboer, A. P., & Bal, R. (2014). Mutual powerlessness in client participation practices in mental health care. *Health Expectations, 17*(2), 208-219. doi:https://dx.doi.org/10.1111/j.1369-7625.2011.00748.x

Bronstein, M. G., & Kakkis, E. D. (2016). Patients as key partners in rare disease drug development. *Nature Reviews. Drug Discovery, 15*(11), 731-732. doi:https://dx.doi.org/10.1038/nrd.2016.133

Brooks, F. (2008). Nursing and public participation in health: an ethnographic study of a patient council. *International Journal of Nursing Studies, 45*(1), 3-13.

Brouwers, M. C., Vukmirovic, M., Spithoff, K., & Makarski, J. (2017). Understanding optimal approaches to patient and caregiver engagement in the development of cancer practice guidelines: a mixed methods study. *BMC Health Services Research, 17*(1), 186. doi:https://dx.doi.org/10.1186/s12913-017-2107-5

Brouwers, M. C., Vukmirovic, M., Spithoff, K., Zwaal, C., McNair, S., & Peek, N. (2018). Engaging cancer patients in clinical practice guideline development: a pilot study. *Current Oncology, 25*(4), 250-256. doi:https://dx.doi.org/10.3747/co.25.3943

Brown, J., Rafferty, J., Golding, D., Adewale, V., Chan, L., Pastorello, C., . . . Neukirch, J. (2016). Patient Engagement for Youth in Multiple Facets of Healthcare in Rhode Island. *Rhode Island Medicine, 99*(8), 19-21.

Brys, N., Keating, J. A., Knobloch, M. J., & Safdar, N. (2019). Engaging patients in health care epidemiology research: A case example. *American Journal of Infection Control, 47*(2), 139-143. doi:10.1016/j.ajic.2018.08.013

Buck, D., Gamble, C., Dudley, L., Preston, J., Hanley, B., Williamson, P. R., . . . Group, E. P. A. (2014). From plans to actions in patient and public involvement: qualitative study of documented plans and the accounts of researchers and patients sampled from a cohort of clinical trials. *BMJ Open, 4*(12), e006400. doi:https://dx.doi.org/10.1136/bmjopen-2014-006400

Buck, D. S., Rochon, D., Davidson, H., McCurdy, S., & Committee of Helthcare for the, H.-H. (2004). Involving homeless persons in the leadership of a health care organization. *Qualitative Health Research, 14*(4), 513-525.

Burns, K. E. A., Devlin, J. W., & Hill, N. S. (2017). Patient and Family Engagement in Designing and Implementing a Weaning Trial: A Novel Research Paradigm in Critical Care. *Chest, 152*(4), 707-711. doi:https://dx.doi.org/10.1016/j.chest.2017.06.028

Burns, K. K., Davis, D., Popescu, I., Laeeque, H., Kossey, S., Misfeldt, R., & Thrall, C. (2020). Patient Engagement in a Large-Scale Change Initiative: "As Safe as Possible, as Soon as Possible". *Healthcare Quarterly, 22*(SP), 27-39. doi:https://dx.doi.org/10.12927/hcq.2020.26049

Caldwell, J., Hauss, S., & Stark, B. (2009). Participation of individuals with developmental disabilities and families on advisory boards and committees. *Journal of Disability Policy Studies, 20*(2), 101-109. Retrieved from http://libaccess.mcmaster.ca/login?url=http://search.ebscohost.com/login.aspx?direct=true&db=cin20&AN=105408509&site=ehost-live&scope=site

Callander, R., Ning, L., Crowley, A., Childs, B., Brisbane, P., & Salter, T. (2011). Consumers and carers as partners in mental health research: reflections on the experience of two project teams in Victoria, Australia. *International Journal of Mental Health Nursing, 20*(4), 263-273. doi:https://dx.doi.org/10.1111/j.1447-0349.2010.00731.x

Callard, F., Rose, D., & Wykes, T. (2012). Close to the bench as well as at the bedside: involving service users in all phases of translational research. *Health Expectations, 15*(4), 389-400. doi:https://dx.doi.org/10.1111/j.1369-7625.2011.00681.x

Campbell, M., & Martin, M. (2010). Reducing health inequalities in Scotland: the involvement of people with learning disabilities as national health service reviewers. *British Journal of Learning Disabilities, 38*(1), 49-58. doi:10.1111/j.1468-3156.2009.00562.x

Campbell, M. M., Susser, E., de Vries, J., Baldinger, A., Sibeko, G., Mndini, M. M., . . . Stein, D. J. (2015). Exploring researchers' experiences of working with a researcher-driven, population-specific community advisory board in a South African schizophrenia genomics study. *BMC Medical Ethics, 16*, 45. doi:https://dx.doi.org/10.1186/s12910-015-0037-5

Campbell, T., Murat, J., McMaster, D., & Hardwick, I. (2007). Developing user involvement in HIV services in London. *HIV Nursing, 7*(4), 14-17. Retrieved from http://libaccess.mcmaster.ca/login?url=http://search.ebscohost.com/login.aspx?direct=true&db=cin20&AN=105840204&site=ehost-live&scope=site

Canfield, C. (2018). The Capacity for Patient Engagement: What Patient Experiences Tell Us About What's Ahead. *Healthcare Quarterly, 21*, 68-72. Retrieved from http://libaccess.mcmaster.ca/login?url=http://search.ebscohost.com/login.aspx?direct=true&db=cin20&AN=133597665&site=ehost-live&scope=site

Carman, K. L., Dardess, P., Maurer, M., Sofaer, S., Adams, K., Bechtel, C., & Sweeney, J. (2013). Patient and family engagement: a framework for understanding the elements and developing interventions and policies. *Health Affairs, 32*(2), 223-231. doi:https://dx.doi.org/10.1377/hlthaff.2012.1133

Caron-Flinterman, J. F., Broerse, J. E., & Bunders, J. F. (2005). The experiential knowledge of patients: a new resource for biomedical research? *Social Science & Medicine, 60*(11), 2575-2584.

Caron-Flinterman, J. F., Broerse, J. E. W., & Bunders, J. F. G. (2007). Patient partnership in decision-making on biomedical research - Changing the network. *Science Technology & Human Values, 32*(3), 339-368. doi:10.1177/0162243906298354

Carr, E. C. J., Patel, J. N., Ortiz, M. M., Miller, J. L., Teare, S. R., Barber, C. E. H., & Marshall, D. A. (2019). Co-design of a patient experience survey for arthritis central intake: an example of meaningful patient engagement in healthcare design. *BMC Health Services Research, 19*(1), 355. doi:https://dx.doi.org/10.1186/s12913-019-4196-9

Carroll, S. L., Embuldeniya, G., Abelson, J., McGillion, M., Berkesse, A., & Healey, J. S. (2017). Questioning patient engagement: research scientists' perceptions of the challenges of patient engagement in a cardiovascular research network. *Patient preference & adherence, 11*, 1573-1583. doi:https://dx.doi.org/10.2147/PPA.S135457

Carter, P., & Martin, G. (2018). Engagement of patients and the public in NHS sustainability and transformation: An ethnographic study. *Critical Social Policy, 38*(4), 707-727. doi:10.1177/0261018317749387

Cassels, A. (2016). Patient speaking for patients. *International Journal of Health Governance, 21*(2), 89-95. doi:10.1108/IJHG-02-2016-0014

Cawston, P. G., & Barbour, R. S. (2003). Clients or citizens? Some considerations for primary care organisations. *British Journal of General Practice, 53*(494), 716-722.

Celenza, J. F., Zayack, D., Buus-Frank, M. E., & Horbar, J. D. (2017). Family Involvement in Quality Improvement: From Bedside Advocate to System Advisor. *Clinics in Perinatology, 44*(3), 553-566. doi:https://dx.doi.org/10.1016/j.clp.2017.05.008

Chalmers, J. D., Timothy, A., Polverino, E., Almagro, M., Ruddy, T., Powell, P., & Boyd, J. (2017). Patient participation in ERS guidelines and research projects: the EMBARC experience. *Breathe, 13*(3), 194-207. doi:https://dx.doi.org/10.1183/20734735.009517

Chambers, E., Gardiner, C., Thompson, J., & Seymour, J. (2019). Patient and carer involvement in palliative care research: An integrative qualitative evidence synthesis review. *Palliative Medicine, 33*(8), 969-984. doi:10.1177/0269216319858247

Chambers, M., McAndrew, S., Nolan, F., Thomas, B., Watts, P., & Kantaris, X. (2017). Service user involvement in the coproduction of a mental health nursing metric: The Therapeutic Engagement Questionnaire. *Health Expectations, 20*(5), 871-877. doi:https://dx.doi.org/10.1111/hex.12526

Chamney, M. (2014). Renal service users' and carers' collaboration to improve education and research in the UK: an update four years on. *Renal Society of Australasia Journal, 10*(3), 102-105. Retrieved from http://libaccess.mcmaster.ca/login?url=http://search.ebscohost.com/login.aspx?direct=true&db=cin20&AN=107838592&site=ehost-live&scope=site

Chiu, C. G., Mitchell, T. L., & Fitch, M. I. (2013). From patient to participant: enhancing the validity and ethics of cancer research through participatory research. *Journal of Cancer Education, 28*(2), 237-246. doi:https://dx.doi.org/10.1007/s13187-013-0464-2

Chu, L. F., Utengen, A., Kadry, B., Kucharski, S. E., Campos, H., Crockett, J., . . . Clauson, K. A. (2016). "Nothing about us without us"-patient partnership in medical conferences. *BMJ, 354*, i3883. doi:https://dx.doi.org/10.1136/bmj.i3883

Chung, J. S., Young, H. N., Moreno, M. A., Kliems, H., & Cox, E. D. (2017). Patient-centred outcomes research: brave new world meets old institutional policies. *Family Practice, 34*(3), 296-300. doi:https://dx.doi.org/10.1093/fampra/cmw129

Chung, K., & Lounsbury, D. W. (2006). The role of power, process, and relationships in participatory research for statewide HIV/AIDS programming. *Social Science & Medicine, 63*(8), 2129-2140.

Ciccarella, A., Staley, A. C., & Franco, A. T. (2018). Transforming research: engaging patient advocates at all stages of cancer research. *Annals of Translational Medicine, 6*(9), 167. doi:https://dx.doi.org/10.21037/atm.2018.04.46

Clarke, C. L., Wilkinson, H., Watson, J., Wilcockson, J., Kinnaird, L., & Williamson, T. (2018). A Seat Around the Table: Participatory Data Analysis With People Living With Dementia. *Qualitative Health Research, 28*(9), 1421-1433. doi:https://dx.doi.org/10.1177/1049732318774768

Clauser, S. B., Gayer, C., Murphy, E., Majhail, N. S., & Baker, K. S. (2015). Patient Centeredness and Engagement in Quality-of-Care Oncology Research. *Journal of oncology practice/American Society of Clinical Oncology, 11*(3), 176-179. doi:https://dx.doi.org/10.1200/JOP.2015.003749

Clavel, N., Pomey, M. P., & Ghadiri, D. P. S. (2019). Partnering with patients in quality improvement: towards renewed practices for healthcare organization managers? *BMC Health Services Research, 19*(1), 815. doi:https://dx.doi.org/10.1186/s12913-019-4618-8

Clay, A. S., & Misak, C. (2016). Engaging Survivors of Critical Illness in Health Care Assessment and Policy Development. Ethical and Practical Complexities. *Annals of the American Thoracic Society, 13*(11), 1871-1876.

Clearfield, E., Tambor, E., Janssen, E. M., & Messner, D. A. (2020). Increasing the Patient-Centeredness of Health Economics and Outcomes Research Through Patient Engagement in Core Outcome Set Development. *The Patient: Patient Centered Outcomes Research, 24*, 24. doi:https://dx.doi.org/10.1007/s40271-020-00424-9

Coates, D., & Howe, D. (2016). Integrating a youth participation model in a youth mental health service: Challenges and lessons learned. *Child & Youth Services, 37*(3), 287-300. doi:10.1080/0145935X.2015.1119652

Cohen, P. D., Herman, L., Jedlinski, S., Willocks, P., & Wittekind, P. (2007). Ethical issues in clinical neuroscience research: a patient's perspective. *Neurotherapeutics, 4*(3), 537-544.

Coleman, S., & Muir, D. (2015). Patient involvement in risk tool development. *Nursing Times, 111*(25), 17-19. Retrieved from http://libaccess.mcmaster.ca/login?url=http://search.ebscohost.com/login.aspx?direct=true&db=cin20&AN=109812837&site=ehost-live&scope=site

Collier, A., & Wyer, M. (2016). Researching Reflexively With Patients and Families: Two Studies Using Video-Reflexive Ethnography to Collaborate With Patients and Families in Patient Safety Research. *Qualitative Health Research, 26*(7), 979-993. doi:https://dx.doi.org/10.1177/1049732315618937

Collins, S. P., Levy, P. D., Holl, J. L., Butler, J., Khan, Y., Israel, T. L., . . . Yancy, C. W. (2017). Incorporating Patient and Caregiver Experiences Into Cardiovascular Clinical Trial Design. *JAMA Cardiology, 2*(11), 1263-1269. doi:https://dx.doi.org/10.1001/jamacardio.2017.3606

Collyar, D. (2005). How have patient advocates in the United States benefited cancer research? *Nature Reviews. Cancer, 5*(1), 73-78. doi:https://dx.doi.org/10.1038/nrc1530

Colombo, C., Moja, L., Gonzalez-Lorenzo, M., Liberati, A., & Mosconi, P. (2012). Patient empowerment as a component of health system reforms: rights, benefits and vested interests. *Internal & Emergency Medicine, 7*(2), 183-187. doi:https://dx.doi.org/10.1007/s11739-012-0757-1

Compagni, A., & Manderscheid, R. W. (2006). A neuroscientist-consumer alliance to transform mental health care. *Journal of Behavioral Health Services & Research, 33*(2), 265-274.

Concannon, T. W., Meissner, P., Grunbaum, J. A., McElwee, N., Guise, J. M., Santa, J., . . . Leslie, L. K. (2012). A New Taxonomy for Stakeholder Engagement in Patient-Centered Outcomes Research. *Journal of General Internal Medicine, 27*(8), 985-991. doi:10.1007/s11606-012-2037-1

Cook, G., & Klein, B. (2005). Involvement of older people in care, service and policy planning. *Journal of Clinical Nursing, 14 Suppl 1*, 43-47.

Coon, J. T., Gwernan-Jones, R., Moore, D., Richardson, M., Shotton, C., Pritchard, W., . . . Ford, T. (2016). End-user involvement in a systematic review of quantitative and qualitative research of non-pharmacological interventions for attention deficit hyperactivity disorder delivered in school settings: reflections on the impacts and challenges. *Health Expectations, 19*(5), 1084-1097. doi:https://dx.doi.org/10.1111/hex.12400

Cosper, P., Kaplow, R., & Moss, J. (2018). The Impact of Patient and Family Advisors on Critical Care Nurses' Empathy. *Journal of Nursing Administration, 48*(12), 622-628. doi:10.1097/nna.0000000000000692

Costello, W., & Dorris, E. (2019). Laying the groundwork: Building relationships for public and patient involvement in pre-clinical paediatric research. *Health Expectations, 18*, 18. doi:https://dx.doi.org/10.1111/hex.12972

Coulman, K. D., Nicholson, A., Shaw, A., Daykin, A., Selman, L. E., Macefield, R., . . . Lane, J. A. (2020). Understanding and optimising patient and public involvement in trial oversight: an ethnographic study of eight clinical trials. *Trials [Electronic Resource], 21*(1), 543. doi:https://dx.doi.org/10.1186/s13063-020-04495-9

Coultas, C., Kieslich, K., & Littlejohns, P. (2019). Patient and public involvement in priority‐setting decisions in England's Transforming NHS: An interview study with Clinical Commissioning Groups in South London sustainability transformation partnerships. *Health Expectations, 22*(6), 1223-1230. doi:10.1111/hex.12948

Coupe, N., & Mathieson, A. (2020). Patient and public involvement in doctoral research: Impact, resources and recommendations. *Health Expectations, 23*(1), 125-136. doi:https://dx.doi.org/10.1111/hex.12976

Cowan, S., Banks, D., Crawshaw, P., & Clifton, A. (2011). Mental health service user involvement in policy development: social inclusion or disempowerment? *Mental Health Review Journal, 16*(4), 177-184. Retrieved from http://libaccess.mcmaster.ca/login?url=http://search.ebscohost.com/login.aspx?direct=true&db=cin20&AN=104508940&site=ehost-live&scope=site

Cox, L. G., & Simpson, A. (2015). Cultural safety, diversity and the servicer user and carer movement in mental health research. *Nursing Inquiry, 22*(4), 306-316. doi:https://dx.doi.org/10.1111/nin.12096

Cox, R., Kendall, M., Molineux, M., Miller, E., & Tanner, B. (2021). Consumer engagement in occupational therapy health‐related research: A scoping review of the Australian Occupational Therapy Journal and a call to action. *Australian Occupational Therapy Journal, 68*(2), 180-192. doi:10.1111/1440-1630.12704

Crocker, J. C., Boylan, A. M., Bostock, J., & Locock, L. (2017). Is it worth it? Patient and public views on the impact of their involvement in health research and its assessment: a UK-based qualitative interview study. *Health Expectations, 20*(3), 519-528. doi:https://dx.doi.org/10.1111/hex.12479

Crocker, J. C., Ricci-Cabello, I., Parker, A., Hirst, J. A., Chant, A., Petit-Zeman, S., . . . Rees, S. (2018). Impact of patient and public involvement on enrolment and retention in clinical trials: systematic review and meta-analysis. *Bmj-British Medical Journal, 363*, 17. doi:10.1136/bmj.k4738

Cukor, D., Cohen, L. M., Cope, E. L., Ghahramani, N., Hedayati, S. S., Hynes, D. M., . . . Mehrotra, R. (2016). Patient and Other Stakeholder Engagement in Patient-Centered Outcomes Research Institute Funded Studies of Patients with Kidney Diseases. *Clinical Journal of The American Society of Nephrology: CJASN, 11*(9), 1703-1712. doi:https://dx.doi.org/10.2215/CJN.09780915

Cunningham, R., & Walton, M. K. (2016). Partnering With Patients to Improve Care: The Value of Patient and Family Advisory Councils. *Journal of Nursing Administration, 46*(11), 549-551.

Curry, L. C., Walker, C., Hogstel, M. O., & Walker, M. B. (2007). A study of Family Councils in nursing homes. *Geriatric Nursing, 28*(4), 245-253.

Curtis, S., Martin, H., DiNella, M., Lavallee, B., Chartrand, C., McLeod, L., . . . Komenda, P. (2021). Kidney Check Point-of-Care Testing-Furthering Patient Engagement and Patient-Centered Care in Canada's Rural and Remote Indigenous Communities: Program Report. *Canadian Journal of Kidney Health & Disease, 8*, 20543581211003744. doi:https://dx.doi.org/10.1177/20543581211003744

Cusack, L., & Sealey-Lapeš, C. (2000). Clinical Governance and User Involvement. *British Journal of Occupational Therapy, 63*(11), 539-546. Retrieved from http://libaccess.mcmaster.ca/login?url=http://search.ebscohost.com/login.aspx?direct=true&db=cin20&AN=104158262&site=ehost-live&scope=site

Czulada, L., Leino, P., & Willis, T. S. (2016). Partnering With a Family Advisor to Improve Communication in a Pediatric Intensive Care Unit. *American Journal of Medical Quality, 31*(6), 520-525.

Dadich, A., & Wyer, M. (2018). Patient Involvement in Healthcare-Associated Infection Research: A Lexical Review. *Infection Control & Hospital Epidemiology, 39*(6), 710-717. doi:https://dx.doi.org/10.1017/ice.2018.62

Darling, E., & Parra, M. A. (2013). Involving patients and the public in research. *Nurse Researcher, 20*(6), 21-25.

Dayton, L., Buttress, A., Agosti, J., Aceves, J., Kieschnick, M., Popejoy, A., . . . Farinholt, K. (2016). Practical Steps to Integrate Family Voice in Organization, Policy, Planning, and Decision-Making for Socio-Emotional Trauma-Informed Integrated Pediatric Care. *Current Problems in Pediatric & Adolescent Health Care, 46*(12), 402-410. doi:https://dx.doi.org/10.1016/j.cppeds.2016.11.005

de Souza, S., Galloway, J., Simpson, C., Chura, R., Dobson, J., Gullick, N. J., . . . Lempp, H. (2017). Patient involvement in rheumatology outpatient service design and delivery: a case study. *Health Expectations, 20*(3), 508-518. doi:https://dx.doi.org/10.1111/hex.12478

de Wit, M., Guillemin, F., Grimm, S., Boonen, A., Fautrel, B., & Joore, M. (2020). Patient engagement in health technology assessment (HTA) and the regulatory process: what about rheumatology? *RMD Open, 6*(3). doi:https://dx.doi.org/10.1136/rmdopen-2020-001286

DeCamp, L. R., Gregory, E., Polk, S., Chrismer, M. C., Giusti, F., Thompson, D. A., & Sibinga, E. (2015). A Voice and a Vote: The Advisory Board Experiences of Spanish-Speaking Latina Mothers. *Hispanic Health Care International : The Official Journal of The National Association of Hispanic Nurses, 13*(4), 217-226. doi:https://dx.doi.org/10.1891/1540-4153.13.4.217

Degeling, C., Carter, S. M., & Rychetnik, L. (2015). Which public and why deliberate?--A scoping review of public deliberation in public health and health policy research. *Social Science & Medicine, 131*, 114-121. doi:https://dx.doi.org/10.1016/j.socscimed.2015.03.009

den Oudendammer, W. M., Noordhoek, J., Abma-Schouten, R. Y., van Houtum, L., Broerse, J. E. W., & Dedding, C. W. M. (2019). Patient participation in research funding: an overview of when, why and how amongst Dutch health funds. *Research Involvement & Engagement, 5*, 33. doi:https://dx.doi.org/10.1186/s40900-019-0163-1

Deng, C. Y., & Wu, C. L. (2010). An innovative participatory method for newly democratic societies: The "civic groups forum" on national health insurance reform in Taiwan. *Social Science & Medicine, 70*(6), 896-903. doi:https://dx.doi.org/10.1016/j.socscimed.2009.10.053

Dent, M., Fallon, C., Wendt, C., Vuori, J., Puhor, M., de Pietro, C., & Silva, S. (2011). Medicine and user involvement within European healthcare: a typology for European comparative research. *International Journal of Clinical Practice, 65*(12), 1218-1220. doi:10.1111/j.1742-1241.2011.02803.x

Desai, M. U., Bellamy, C., Guy, K., Costa, M., O'Connell, M. J., & Davidson, L. (2019). "If You Want to Know About the Book, Ask the Author": Enhancing Community Engagement Through Participatory Research in Clinical Mental Health Settings. *Behavioral Medicine, 45*(2), 177-187. doi:10.1080/08964289.2019.1587589

Devonport, T. J., Nicholls, W., Johnston, L. H., Gutteridge, R., & Watt, A. (2018). It's not just 'What' you do, it's also the 'Way' that you do it: Patient and Public Involvement in the Development of Health Research. *International Journal for Quality in Health Care, 30*(2), 152-156. doi:https://dx.doi.org/10.1093/intqhc/mzx177

Di Lorito, C., Godfrey, M., Dunlop, M., Bosco, A., Pollock, K., Wardt, V., & Harwood, R. H. (2020). Adding to the knowledge on Patient and Public Involvement: Reflections from an experience of co‐research with carers of people with dementia. *Health Expectations, 23*(3), 691-706. doi:10.1111/hex.13049

Diamond, B., Parkin, G., Morris, K., Bettinis, J., & Bettesworth, C. (2003). User involvement: substance or spin? *Journal of Mental Health, 12*(6), 613-626. Retrieved from http://libaccess.mcmaster.ca/login?url=http://search.ebscohost.com/login.aspx?direct=true&db=cin20&AN=106751380&site=ehost-live&scope=site

Dillon, C., Knapp, J., & Stinson, M. (2020). An Evolved Approach to Advisory Boards in Rare Disease Drug Development: 5-Step Model to Finding and Engaging Patient Advisors. *Journal of Patient Experience, 7*(6), 978-981. doi:https://dx.doi.org/10.1177/2374373520948441

Domecq, J. P., Prutsky, G., Elraiyah, T., Wang, Z., Nabhan, M., Shippee, N., . . . Murad, M. H. (2014). Patient engagement in research: a systematic review. *BMC Health Services Research, 14*, 89. doi:https://dx.doi.org/10.1186/1472-6963-14-89

Dovey‐Pearce, G., Rapley, T., Walker, S., Fairgrieve, S., & Parker, M. (2019). The burden of proof: The process of involving young people in research. *Health Expectations, 22*(3), 465-474. doi:10.1111/hex.12870

d'Udekem, Y., Forsdick, V., & du Plessis, K. (2018). Involvement of patients and parents in research undertaken by the Australian and New Zealand Fontan Registry. *Cardiology in the Young, 28*(4), 517-521. doi:https://dx.doi.org/10.1017/S1047951117001494

Dudley, L., Gamble, C., Preston, J., Buck, D., Group, E. P. A., Hanley, B., . . . Young, B. (2015). What Difference Does Patient and Public Involvement Make and What Are Its Pathways to Impact? Qualitative Study of Patients and Researchers from a Cohort of Randomised Clinical Trials. *PLoS ONE [Electronic Resource], 10*(6), e0128817. doi:https://dx.doi.org/10.1371/journal.pone.0128817

Duffett, L. (2017). Patient engagement: What partnering with patient in research is all about. *Thrombosis Research, 150*, 113-120. doi:https://dx.doi.org/10.1016/j.thromres.2016.10.029

Edwards, L., Monro, M., Butterfield, Y., Johl, R., Loftsgard, K. C., Pelletier, H., . . . Lavergne, M. R. (2019). What matters most to patients about primary healthcare: mixed-methods patient priority setting exercises within the PREFeR (PRioritiEs For Research) project. *BMJ Open, 9*(7), e025954. doi:https://dx.doi.org/10.1136/bmjopen-2018-025954

Elberse, J. E., Caron-Flinterman, J. F., & Broerse, J. E. (2011). Patient-expert partnerships in research: how to stimulate inclusion of patient perspectives. *Health Expectations, 14*(3), 225-239. doi:https://dx.doi.org/10.1111/j.1369-7625.2010.00647.x

Elberse, J. E., Pittens, C. A., de Cock Buning, T., & Broerse, J. E. (2012). Patient involvement in a scientific advisory process: setting the research agenda for medical products. *Health Policy, 107*(2-3), 231-242. doi:https://dx.doi.org/10.1016/j.healthpol.2012.05.014

Eley, R. M. (2016). Telling it as it is: involving people with dementia and family carers in policy making, service design and workforce development. *Working with Older People: Community Care Policy & Practice, 20*(4), 219-222. doi:10.1108/WWOP-09-2016-0026

Elliott, M. J., Allu, S., Beaucage, M., McKenzie, S., Kappel, J., Harvey, R., . . . Manns, B. (2021). Defining the Scope of Knowledge Translation Within a National, Patient-Oriented Kidney Research Network. *Canadian Journal of Kidney Health & Disease, 8*, 20543581211004803. doi:https://dx.doi.org/10.1177/20543581211004803

Enzinger, A. C., Wind, J. K., Frank, E., McCleary, N. J., Porter, L., Cushing, H., . . . Schrag, D. (2017). A stakeholder-driven approach to improve the informed consent process for palliative chemotherapy. *Patient Education & Counseling, 100*(8), 1527-1536. doi:https://dx.doi.org/10.1016/j.pec.2017.03.024

Evans, B. A., Bedson, E., Bell, P., Hutchings, H., Lowes, L., Rea, D., . . . West Wales Organisation for Rigorous Trials in, H. (2013). Involving service users in trials: developing a standard operating procedure. *Trials [Electronic Resource], 14*, 219. doi:https://dx.doi.org/10.1186/1745-6215-14-219

Evette, J. L., Addis, M., Cartwright, C., Cowan, P., Duhrkoop, D., Farrell, M., . . . Von Korff, M. (2020). Patient Advisory Committee for a Chronic Opioid Therapy Risk Reduction Evaluation: Engaging Diverse Patients. *Progress in Community Health Partnerships, 14*(4), 481-488. doi:https://dx.doi.org/10.1353/cpr.2020.0053

Ewalds Mulliez, A. P., Pomey, M. P., Bordeleau, J., Desbiens, F., & Pelletier, J. F. (2018). A voice for the patients: Evaluation of the implementation of a strategic organizational committee for patient engagement in mental health. *PLoS ONE [Electronic Resource], 13*(10), e0205173. doi:https://dx.doi.org/10.1371/journal.pone.0205173

Fairbrother, P., McCloughan, L., Adam, G., Brand, R., Brown, C., Watson, M., . . . McKinstry, B. (2016). Involving patients in clinical research: the Telescot Patient Panel. *Health Expectations, 19*(3), 691-701. doi:https://dx.doi.org/10.1111/hex.12132

Fancott, C., Baker, G. R., Judd, M., Humphrey, A., & Morin, A. (2018). Supporting Patient and Family Engagement for Healthcare Improvement: Reflections on "Engagement-Capable Environments" in Pan-Canadian Learning Collaboratives. *Healthcare Quarterly, 21*(SP), 12-30. doi:https://dx.doi.org/10.12927/hcq.2018.25642

Faulkner, M., Alikhaani, J., Brown, L., Cruz, H., Davidson, D., Gregoire, K., . . . Pletcher, M. J. (2018). Exploring Meaningful Patient Engagement in ADAPTABLE (Aspirin Dosing: A Patient-centric Trial Assessing Benefits and Long-term Effectiveness). *Medical Care, 56 Suppl 10 Suppl 1*, S11-S15. doi:https://dx.doi.org/10.1097/MLR.0000000000000949

Fernandes Agreli, H., Murphy, M., Creedon, S., Ni Bhuachalla, C., O'Brien, D., Gould, D., . . . Hegarty, J. (2019). Patient involvement in the implementation of infection prevention and control guidelines and associated interventions: a scoping review. *BMJ Open, 9*(3), e025824. doi:https://dx.doi.org/10.1136/bmjopen-2018-025824

Fleurence, R. L., Beal, A. C., Sheridan, S. E., Johnson, L. B., & Selby, J. V. (2014). Patient-powered research networks aim to improve patient care and health research. *Health Affairs, 33*(7), 1212-1219. doi:https://dx.doi.org/10.1377/hlthaff.2014.0113

Flowers, M., Gunderson, J., & Hall, D. (2020). Evaluating the Patient Family Advisor Experience in Patient-Oriented Research. *Healthcare Quarterly, 23*(1), 28-33. doi:https://dx.doi.org/10.12927/hcq.2020.26142

Forster, R., & Gabe, J. (2008). Voice or choice? Patient and public involvement in the National Health Service in England under New Labour. *International Journal of Health Services, 38*(2), 333-356.

Forsythe, L., Heckert, A., Margolis, M. K., Schrandt, S., & Frank, L. (2018). Methods and impact of engagement in research, from theory to practice and back again: early findings from the Patient-Centered Outcomes Research Institute. *Quality of Life Research, 27*(1), 17-31. doi:https://dx.doi.org/10.1007/s11136-017-1581-x

Forsythe, L. P., Carman, K. L., Szydlowski, V., Fayish, L., Davidson, L., Hickam, D. H., . . . Anyanwu, C. U. (2019). Patient Engagement In Research: Early Findings From The Patient-Centered Outcomes Research Institute. *Health Affairs, 38*(3), 359-367. doi:https://dx.doi.org/10.1377/hlthaff.2018.05067

Forsythe, L. P., Ellis, L. E., Edmundson, L., Sabharwal, R., Rein, A., Konopka, K., & Frank, L. (2016). Patient and Stakeholder Engagement in the PCORI Pilot Projects: Description and Lessons Learned. *Journal of General Internal Medicine, 31*(1), 13-21. doi:10.1007/s11606-015-3450-z

Forsythe, L. P., Szydlowski, V., Murad, M. H., Ip, S., Wang, Z., Elraiyah, T. A., . . . Hickam, D. H. (2014). A systematic review of approaches for engaging patients for research on rare diseases. *Journal of General Internal Medicine, 29 Suppl 3*, S788-800. doi:https://dx.doi.org/10.1007/s11606-014-2895-9

Foster, M., Fergusson, D. A., Hawrysh, T., Presseau, J., Kekre, N., Schwartz, S., . . . Lalu, M. M. (2020). Partnering with patients to get better outcomes with chimeric antigen receptor T-cell therapy: towards engagement of patients in early phase trials. *Research Involvement & Engagement, 6*, 61. doi:https://dx.doi.org/10.1186/s40900-020-00230-5

Fraenkel, L., Miller, A. S., Clayton, K., Crow-Hercher, R., Hazel, S., Johnson, B., . . . Nowell, W. B. (2016). When Patients Write the Guidelines: Patient Panel Recommendations for the Treatment of Rheumatoid Arthritis. *Arthritis care & research, 68*(1), 26-35. doi:https://dx.doi.org/10.1002/acr.22758

Frank, L., Forsythe, L., Ellis, L., Schrandt, S., Sheridan, S., Gerson, J., . . . Daugherty, S. (2015). Conceptual and practical foundations of patient engagement in research at the patient-centered outcomes research institute. *Quality of Life Research, 24*(5), 1033-1041. doi:https://dx.doi.org/10.1007/s11136-014-0893-3

Freedman, D. B. (2006). Involvement of patients in Clinical Governance. *Clinical Chemistry & Laboratory Medicine, 44*(6), 699-703.

Frost, J., Gibson, A., Harris-Golesworthy, F., Harris, J., & Britten, N. (2018). Patient involvement in qualitative data analysis in a trial of a patient-centred intervention: Reconciling lay knowledge and scientific method. *Health Expectations, 21*(6), 1111-1121. doi:https://dx.doi.org/10.1111/hex.12814

Fudge, N., Wolfe, C. D., & McKevitt, C. (2007). Involving older people in health research. *Age & Ageing, 36*(5), 492-500.

Gagnon, J., Abramovitch, A., Caminsky, S., Jack, S., Reoch, J., & Hersson-Edery, F. (2020). Participation in the Diabetes Empowerment Group Program Research Project: Patient Partners' Perspectives. *Canadian Journal of Diabetes, 44*(5), 442-444. doi:https://dx.doi.org/10.1016/j.jcjd.2020.01.002

Gagnon, M. P., Candas, B., Desmartis, M., Gagnon, J., La Roche, D., Rhainds, M., . . . Legare, F. (2014). Involving patient in the early stages of health technology assessment (HTA): a study protocol. *BMC Health Services Research, 14*, 273. doi:https://dx.doi.org/10.1186/1472-6963-14-273

Gagnon, M. P., Dipankui, M. T., Poder, T. G., Payne-Gagnon, J., Mbemba, G., & Beretta, V. (2021). Patient and public involvement in health technology assessment: update of a systematic review of international experiences. *International Journal of Technology Assessment in Health Care, 37*, 16. doi:10.1017/s0266462321000064

Gagnon, M. P., Gagnon, J., St-Pierre, M., Gauvin, F. P., Piron, F., Rhainds, M., . . . Legare, F. (2012). Involving patients in HTA activities at local level: a study protocol based on the collaboration between researchers and knowledge users. *BMC Health Services Research, 12*, 14. doi:https://dx.doi.org/10.1186/1472-6963-12-14

Garnett, R. T., Bowman, J., & Ganton, J. (2017). Patient and Citizen Innovation Council in family practice. *Canadian Family Physician, 63*(2), e102-e106.

Ghersi, D. (2002). Making it happen: approaches to involving consumers in Cochrane reviews. *Evaluation & the Health Professions, 25*(3), 270-283.

Giebel, C., Roe, B., Hodgson, A., Britt, D., Clarkson, P., Ho, S. T. D. H. S. D. P., & Patient Public Carer Involvement, G. (2019). Effective public involvement in the HoST-D Programme for dementia home care support: From proposal and design to methods of data collection (innovative practice). *Dementia-International Journal of Social Research and Practice, 18*(7-8), 3173-3186. doi:10.1177/1471301216687698

Gillard, S., Simons, L., Turner, K., Lucock, M., & Edwards, C. (2012). Patient and public involvement in the coproduction of knowledge: reflection on the analysis of qualitative data in a mental health study. *Qualitative Health Research, 22*(8), 1126-1137. doi:https://dx.doi.org/10.1177/1049732312448541

Gilmore, N. J., Canin, B., Whitehead, M., Sedenquist, M., Griggs, L., Finch, L., . . . Mohile, S. G. (2019). Engaging older patients with cancer and their caregivers as partners in cancer research. *Cancer, 125*(23), 4124-4133. doi:10.1002/cncr.32402

Goel, N. (2020). Conducting research in psoriatic arthritis: the emerging role of patient research partners. *Rheumatology, 59*(Suppl 1), i47-i55. doi:https://dx.doi.org/10.1093/rheumatology/kez338

Gold, S. K., Abelson, J., & Charles, C. A. (2005). From rhetoric to reality: including patient voices in supportive cancer care planning. *Health Expectations, 8*(3), 195-209.

Gombeski, W. R., Jr., Taylor, J., Britt, J., Riggs, K., Wray, T., Springate, S., . . . Bernard, P. (2010). Customer advisory groups: another way to listen to the marketplace. *Health Marketing Quarterly, 27*(1), 86-96. doi:https://dx.doi.org/10.1080/07359680903519826

Gordon, L., Dickinson, A., Offredy, M., & Smiddy, J. (2017). A research note on the benefit of patient and public involvement in research: The experience of prostate cancer patients regarding information in radiotherapy. *Radiography (London), 23*(2), 167-170. doi:https://dx.doi.org/10.1016/j.radi.2017.02.004

Graham, N., Mandy, A., Clarke, C., & Morriss-Roberts, C. (2017). Using children and young people as advocates to inform research design. *British Journal of Occupational Therapy, 80*(11), 684-688. doi:10.1177/0308022617725491

Greene, J., Farley, D., Amy, C., & Hutcheson, K. (2018). How Patient Partners Influence Quality Improvement Efforts. *Joint Commission Journal on Quality & Patient Safety, 44*(4), 186-195. doi:https://dx.doi.org/10.1016/j.jcjq.2017.09.006

Gregory, S., Wells, K., Forysth, K., Latto, C., Szyra, H., Saunders, S., . . . Milne, R. (2018). Research participants as collaborators: Background, experience and policies from the PREVENT Dementia and EPAD programmes. *Dementia, 17*(8), 1045-1054. doi:https://dx.doi.org/10.1177/1471301218789307

Grogan, A., Coughlan, M., B, O. M., & McKee, G. (2012). The development of a patient partnership programme and its impact on quality improvements in a comprehensive haemophilia care service. *Haemophilia, 18*(6), 875-880. doi:https://dx.doi.org/10.1111/j.1365-2516.2012.02885.x

Groot, B., Dedding, C., Slob, E., Maitland, H., Teunissen, T., Rutjes, N., & Vijverberg, S. (2021). Adolescents' experiences with patient engagement in respiratory medicine. *Pediatric Pulmonology, 56*(1), 211-216. doi:https://dx.doi.org/10.1002/ppul.25150

Group, U. N. C. C. H. H. P. E., Kixmiller, S., Sloan, A. P., Wadsworth, S., Brown, F., Chaney, L., . . . Thomas, K. (2021). Experiences of an HCV Patient engagement group: a seven-year journey. *Research Involvement & Engagement, 7*(1), 7. doi:https://dx.doi.org/10.1186/s40900-021-00249-2

Grundy, A., Keetharuth, A. D., Barber, R., Carlton, J., Connell, J., Taylor Buck, E., . . . Brazier, J. (2019). Public involvement in health outcomes research: lessons learnt from the development of the recovering quality of life (ReQoL) measures. *Health & Quality of Life Outcomes, 17*(1), 60. doi:https://dx.doi.org/10.1186/s12955-019-1123-z

Gurung, G., Richardson, A., Wyeth, E., Edmonds, L., & Derrett, S. (2020). Child/youth, family and public engagement in paediatric services in high-income countries: A systematic scoping review. *Health Expectations, 23*(2), 261-273. doi:https://dx.doi.org/10.1111/hex.13017

Gustavsson, S. M. K., & Andersson, T. (2019). Patient involvement 2.0: Experience-based co-design supported by action research. *Action Research, 17*(4), 469-491. doi:10.1177/1476750317723965

Haarsma, F., Moser, A., Beckers, M., van Rijswijk, H., Stoffers, E., & Beurskens, A. (2015). The perceived impact of public involvement in palliative care in a provincial palliative care network in the Netherlands: a qualitative study. *Health Expectations, 18*(6), 3186-3200. doi:https://dx.doi.org/10.1111/hex.12308

Hacker, K. E., & Smith, A. B. (2018). Engaging Stakeholders and Patient Partners. *Surgical Oncology Clinics of North America, 27*(4), 665-673. doi:https://dx.doi.org/10.1016/j.soc.2018.05.007

Haesebaert, J., Samson, I., Lee-Gosselin, H., Guay-Belanger, S., Proteau, J. F., Drouin, G., . . . Legare, F. (2020). "They heard our voice!" patient engagement councils in community-based primary care practices: a participatory action research pilot study. *Research Involvement & Engagement, 6*, 54. doi:https://dx.doi.org/10.1186/s40900-020-00232-3

Hamakawa, N., Kogetsu, A., Isono, M., Yamasaki, C., Manabe, S., Takeda, T., . . . Kato, K. (2021). The practice of active patient involvement in rare disease research using ICT: experiences and lessons from the RUDY JAPAN project. *Research Involvement & Engagement, 7*(1), 9. doi:https://dx.doi.org/10.1186/s40900-021-00253-6

Hamilton, C. B., Hoens, A. M., McQuitty, S., McKinnon, A. M., English, K., Backman, C. L., . . . Li, L. C. (2018). Development and pre-testing of the Patient Engagement In Research Scale (PEIRS) to assess the quality of engagement from a patient perspective. *PLoS ONE [Electronic Resource], 13*(11), e0206588. doi:https://dx.doi.org/10.1371/journal.pone.0206588

Happell, B., Ewart, S. B., Platania‐Phung, C., & Stanton, R. (2016). Participative mental health consumer research for improving physical health care: An integrative review. *International Journal of Mental Health Nursing, 25*(5), 399-408. doi:10.1111/inm.12226

Happell, B., Gordon, S., Bocking, J., Ellis, P., Roper, C., Liggins, J., . . . Scholz, B. (2018). How did I not see that? Perspectives of nonconsumer mental health researchers on the benefits of collaborative research with consumers. *International Journal of Mental Health Nursing, 27*(4), 1230-1239. doi:https://dx.doi.org/10.1111/inm.12453

Happell, B., Gordon, S., Bocking, J., Ellis, P., Roper, C., Liggins, J., . . . Platania-Phung, C. (2018). Turning the Tables: Power Relations Between Consumer Researchers and Other Mental Health Researchers. *Issues in Mental Health Nursing, 39*(8), 633-640. doi:https://dx.doi.org/10.1080/01612840.2018.1445328

Happell, B., Gordon, S., Bocking, J., Ellis, P., Roper, C., Liggins, J., . . . Platania-Phung, C. (2019). "Chipping away": non-consumer researcher perspectives on barriers to collaborating with consumers in mental health research. *Journal of Mental Health, 28*(1), 49-55. doi:https://dx.doi.org/10.1080/09638237.2018.1466051

Happell, B., Gordon, S., Roper, C., Scholz, B., Ellis, P., Waks, S., . . . Platania‐Phung, C. (2020). 'It is always worth the extra effort': Organizational structures and barriers to collaboration with consumers in mental health research: Perspectives of non‐consumer researcher allies. *International Journal of Mental Health Nursing, 29*(6), 1168-1180. doi:10.1111/inm.12757

Happell, B., & Roper, C. (2007). Consumer participation in mental health research: articulating a model to guide practice. *Australasian Psychiatry, 15*(3), 237-241.

Happell, B., & Roper, C. (2009). Promoting genuine consumer participation in mental health education: a consumer academic role. *Nurse Education Today, 29*(6), 575-579. doi:https://dx.doi.org/10.1016/j.nedt.2008.01.004

Happell, B., Scholz, B., Gordon, S., Bocking, J., Ellis, P., Roper, C., . . . Platania-Phung, C. (2018). "I don't think we've quite got there yet": The experience of allyship for mental health consumer researchers. *Journal of Psychiatric & Mental Health Nursing, 25*(8), 453-462. doi:https://dx.doi.org/10.1111/jpm.12476

Harris, J., Graue, M., Dunning, T., Haltbakk, J., Austrheim, G., Skille, N., . . . Kirkevold, M. (2015). Involving people with diabetes and the wider community in diabetes research: a realist review protocol. *Systematic Reviews, 4*, 146. doi:https://dx.doi.org/10.1186/s13643-015-0127-y

Harris, J., Haltbakk, J., Dunning, T., Austrheim, G., Kirkevold, M., Johnson, M., & Graue, M. (2019). How patient and community involvement in diabetes research influences health outcomes: A realist review. *Health Expectations, 22*(5), 907-920. doi:10.1111/hex.12935

Harrison, J. D., Anderson, W. G., Fagan, M., Robinson, E., Schnipper, J., Symczak, G., . . . Auerbach, A. D. (2019). Patient and Family Advisory Councils for Research: Recruiting and Supporting Members From Diverse and Hard-to-Reach Communities. *Journal of Nursing Administration, 49*(10), 473-479. doi:https://dx.doi.org/10.1097/NNA.0000000000000790

Harrison, J. D., Anderson, W. G., Fagan, M., Robinson, E., Schnipper, J., Symczak, G., . . . Auerbach, A. D. (2018). Patient and Family Advisory Councils (PFACs): Identifying Challenges and Solutions to Support Engagement in Research. *The Patient: Patient-Centered Outcomes Research, 11*(4), 413-423. doi:https://dx.doi.org/10.1007/s40271-018-0298-4

Harrison, J. D., Archuleta, M., Avitia, E., Banta, J., Benn, J., Burden, M., . . . Leykum, L. (2020). Developing a Patient- and Family-Centered Research Agenda for Hospital Medicine: The Improving Hospital Outcomes through Patient Engagement (i-HOPE) Study. *Journal of Hospital Medicine (Online), 15*(6), 331-337. doi:https://dx.doi.org/10.12788/jhm.3386

Harrison, J. D., Auerbach, A. D., Anderson, W., Fagan, M., Carnie, M., Hanson, C., . . . Weiss, R. (2019). Patient stakeholder engagement in research: A narrative review to describe foundational principles and best practice activities. *Health Expectations, 22*(3), 307-316. doi:https://dx.doi.org/10.1111/hex.12873

Harrison, M., & Palmer, R. (2015). Exploring patient and public involvement in stroke research: a qualitative study. *Disability & Rehabilitation, 37*(23), 2174-2183. doi:https://dx.doi.org/10.3109/09638288.2014.1001525

Haycock, C., & Wahl, C. (2013). Achieving patient and family engagement through the implementation and evolution of advisory councils across a large health care system. *Nursing Administration Quarterly, 37*(3), 242-246. doi:https://dx.doi.org/10.1097/NAQ.0b013e318295f54c

Hayes, G., Costello, H., Nurock, S., Cornwall, A., & Francis, P. (2018). Ticking boxes or meaningful partnership - The experience of lay representation, participant and study partner involvement in Brains for Dementia Research. *Dementia, 17*(8), 1023-1034. doi:https://dx.doi.org/10.1177/1471301218789308

Healey, A., van Beinum, A., Hornby, L., Wilson, L. C., Bedard, S., Berrigan, H., . . . Shemie, S. D. (2020). Patient engagement in a Canadian consensus forum for heart donation after circulatory determination of death. *Canadian Journal of Anaesthesia, 67*(12), 1738-1748. doi:https://dx.doi.org/10.1007/s12630-020-01808-z

Henderson, J. L., Hawke, L. D., & Relihan, J. (2018). Youth engagement in the YouthCan IMPACT trial. *CMAJ Canadian Medical Association Journal, 190*(Suppl), S10-S12. doi:https://dx.doi.org/10.1503/cmaj.180328

Hillier, D. R., Tang, M., Clark, W., MacDonald, C., Connolly, C., Large, C., . . . Rosenblum, N. D. (2020). A Framework to Ensure Patient Partners Have Equal and Contributing Voices Throughout the Research Program Evaluation Process. *Canadian Journal of Kidney Health & Disease, 7*, 2054358120970093. doi:https://dx.doi.org/10.1177/2054358120970093

Hirst, E., Irving, A., & Goodacre, S. (2016). Patient and public involvement in emergency care research. *Emergency Medicine Journal, 33*(9), 665-670. doi:https://dx.doi.org/10.1136/emermed-2016-205700

Hoeg, B. L., Tjornhoj-Thomsen, T., Skaarup, J. A., Langstrup, H., Zoffmann, V., Saltbaek, L., . . . Bidstrup, P. E. (2019). Whose perspective is it anyway? Dilemmas of patient involvement in the development of a randomized clinical trial - a qualitative study. *Acta Oncologica, 58*(5), 634-641. doi:https://dx.doi.org/10.1080/0284186X.2019.1566776

Hoekstra, F., Martin Ginis, K. A., Allan, V., Kothari, A., & Gainforth, H. L. (2018). Evaluating the impact of a network of research partnerships: a longitudinal multiple case study protocol. *Health Research Policy & Systems, 16*(1), 107. doi:https://dx.doi.org/10.1186/s12961-018-0377-y

Horsfall, J., Cleary, M., Walter, G., & Malins, G. (2007). Challenging conventional practice: placing consumers at the centre of the research enterprise. *Issues in Mental Health Nursing, 28*(11), 1201-1213.

Hovey, R. B., Morck, A., Nettleton, S., Robin, S., Bullis, D., Findlay, A., & Massfeller, H. (2010). Partners in our care: patient safety from a patient perspective. *Quality & Safety in Health Care, 19*(6), e59. doi:https://dx.doi.org/10.1136/qshc.2008.030908

Hruslinski, J., Menio, D. A., Hymes, R. A., Jaffe, J. D., Langlois, C., Ramsey, L., . . . Regional Versus General Anesthesia for Promoting Independence After Hip Fracture, I. (2021). Engaging patients as partners in a multicentre trial of spinal versus general anaesthesia for older adults. *British Journal of Anaesthesia, 126*(2), 395-403. doi:https://dx.doi.org/10.1016/j.bja.2020.09.052

Huang, J., Lipman, P. D., & Daniel Mullins, C. (2017). Bridging the divide: building infrastructure to support community-academic partnerships and improve capacity to conduct patient-centered outcomes research. *Translational Behavioral Medicine, 7*(4), 773-782. doi:https://dx.doi.org/10.1007/s13142-017-0487-z

Hubbard, G., Kidd, L., Donaghy, E., McDonald, C., & Kearney, N. (2007). A review of literature about involving people affected by cancer in research, policy and planning and practice. *Patient Education & Counseling, 65*(1), 21-33.

Hughes, L., Busija, K. R., Chaudhuri, E. R., & Popescu, I. C. (2017). Patients as Front-Line Owners and Partners in Improving Quality and Safety. *Healthcarepapers, 17*(1), 25-28.

Hull, D., Barton, D., Guo, K., Russell, C., Aucott, B., & Wiles, D. (2012). Patient and public involvement to support liver disease research. *British Journal of Nursing, 21*(16), 972-976.

Humphries, S. M., Rondung, E., Norlund, F., Sundin, O., Tornvall, P., Held, C., . . . Olsson, E. M. G. (2020). Designing a Web-Based Psychological Intervention for Patients With Myocardial Infarction With Nonobstructive Coronary Arteries: User-Centered Design Approach. *Journal of Medical Internet Research, 22*(9), e19066. doi:https://dx.doi.org/10.2196/19066

Hutchinson, A., & Lovell, A. (2013). Participatory action research: moving beyond the mental health 'service user' identity. *Journal of Psychiatric & Mental Health Nursing (John Wiley & Sons, Inc.), 20*(7), 641-649. doi:10.1111/jpm.12001

Hutchison, C., & McCreaddie, M. (2007). The process of developing audiovisual patient information: challenges and opportunities. *Journal of Clinical Nursing, 16*(11), 2047-2055. doi:10.1111/j.1365-2702.2006.01758.x

Hwang, A., & Warshaw, G. (2019). Joint AGS-CCEHI Survey Offers Insights into Patient Engagement in Geriatric Clinical Settings. *Journal of the American Geriatrics Society, 67*(9), 1791-1794. doi:https://dx.doi.org/10.1111/jgs.16016

Iliffe, S., McGrath, T., & Mitchell, D. (2013). The impact of patient and public involvement in the work of the Dementias & Neurodegenerative Diseases Research Network (DeNDRoN): case studies. *Health Expectations, 16*(4), 351-361. doi:https://dx.doi.org/10.1111/j.1369-7625.2011.00728.x

Iwata, A. J., Olden, H. A., Kippen, K. E., Swegal, W. C., Johnson, C. C., & Chang, S. S. (2019). Flexible model for patient engagement: Achieving quality outcomes and building a research agenda for head and neck cancer. *Head & Neck, 41*(4), 1087-1093. doi:https://dx.doi.org/10.1002/hed.25584

Iyer, S. P., Pancake, L. S., Dandino, E. S., & Wells, K. B. (2015). Consumer-Involved Participatory Research to Address General Medical Health and Wellness in a Community Mental Health Setting. *Psychiatric Services, 66*(12), 1268-1270. doi:https://dx.doi.org/10.1176/appi.ps.201500157

Jackson, T., Pinnock, H., Liew, S. M., Horne, E., Ehrlich, E., Fulton, O., . . . De Simoni, A. (2020). Patient and public involvement in research: from tokenistic box ticking to valued team members. *Bmc Medicine, 18*(1), 79. doi:https://dx.doi.org/10.1186/s12916-020-01544-7

James, D., & Willitts, M. (2000). Board membership. House of unrepresentatives. *Health Service Journal, 110*(5732), 32-33.

Janamian, T., Crossland, L., & Wells, L. (2016). On the road to value co-creation in health care: the role of consumers in defining the destination, planning the journey and sharing the drive. *Medical Journal of Australia, 204*(7 Suppl), S12-14.

Janney, C. A., Brzoznowski, K. F., Richardson, C. R., Dopp, R. R., Segar, M. L., Ganoczy, D., . . . Valenstein, M. (2017). Moving Towards Wellness: Physical activity practices, perspectives, and preferences of users of outpatient mental health service. *General Hospital Psychiatry, 49*, 63-66. doi:10.1016/j.genhosppsych.2017.07.004

Janvier, A., Bourque, C. J., Dahan, S., Robson, K., Barrington, K. J., & on behalf of the Partenariat Famille, t. (2019). Integrating Parents in Neonatal and Pediatric Research. *Neonatology, 115*(4), 283-291. doi:https://dx.doi.org/10.1159/000492502

Jennings, H., Slade, M., Bates, P., Munday, E., & Toney, R. (2018). Best practice framework for Patient and Public Involvement (PPI) in collaborative data analysis of qualitative mental health research: methodology development and refinement. *Bmc Psychiatry, 18*(1), 213. doi:https://dx.doi.org/10.1186/s12888-018-1794-8

Jewell, A., Pritchard, M., Barrett, K., Green, P., Markham, S., McKenzie, S., . . . Stewart, R. (2019). The Maudsley Biomedical Research Centre (BRC) data linkage service user and carer advisory group: creating and sustaining a successful patient and public involvement group to guide research in a complex area. *Research Involvement & Engagement, 5*, 20. doi:https://dx.doi.org/10.1186/s40900-019-0152-4

Jha, V., Quinton, N. D., Bekker, H. L., & Roberts, T. E. (2009). Strategies and interventions for the involvement of real patients in medical education: a systematic review. *Medical Education, 43*(1), 10-20. doi:https://dx.doi.org/10.1111/j.1365-2923.2008.03244.x

Johnson, B. H. (2016). Promoting Patient- and Family-Centered Care Through Personal Stories. *Academic Medicine, 91*(3), 297-300. doi:https://dx.doi.org/10.1097/ACM.0000000000001086

Johnson, D. S., Bush, M. T., Brandzel, S., & Wernli, K. J. (2016). The patient voice in research-evolution of a role. *Research Involvement & Engagement, 2*, 6. doi:https://dx.doi.org/10.1186/s40900-016-0020-4

Johnson, K. E., Mroz, T. M., Abraham, M., Figueroa Gray, M., Minniti, M., Nickel, W., . . . Hsu, C. (2016). Promoting Patient and Family Partnerships in Ambulatory Care Improvement: A Narrative Review and Focus Group Findings. *Advances in Therapy, 33*(8), 1417-1439. doi:https://dx.doi.org/10.1007/s12325-016-0364-z

Johnstone, M. J., & Kanitsaki, O. (2009). Engaging patients as safety partners: some considerations for ensuring a culturally and linguistically appropriate approach. *Health Policy, 90*(1), 1-7. doi:https://dx.doi.org/10.1016/j.healthpol.2008.08.007

Jones, E., & Hahn, S. (2007). Working in partnership to deliver training. *A Life in the Day, 11*(4), 6-9. Retrieved from http://libaccess.mcmaster.ca/login?url=http://search.ebscohost.com/login.aspx?direct=true&db=cin20&AN=105943506&site=ehost-live&scope=site

Jones, K., & Potter, T. (2019). A Toolkit to Improve Diversity in Patient and Family Advisory Councils: A New Method to Advance Health Equity. *Creative Nursing, 25*(2), 176-181. doi:https://dx.doi.org/10.1891/1078-4535.25.2.176

Jones, M., & Pietila, I. (2018). "The citizen is stepping into a new role"-Policy interpretations of patient and public involvement in Finland. *Health & Social Care in the Community, 26*(2), e304-e311. doi:https://dx.doi.org/10.1111/hsc.12520

Joss, N., Cooklin, A., & Oldenburg, B. (2016). A scoping review of end user involvement in disability research. *Disability & Health Journal, 9*(2), 189-196. doi:https://dx.doi.org/10.1016/j.dhjo.2015.10.001

Kaiser, B. L., Thomas, G. R., & Bowers, B. J. (2017). A Case Study of Engaging Hard-to-Reach Participants in the Research Process: Community Advisors on Research Design and Strategies (CARDS). *Research in Nursing & Health, 40*(1), 70-79. doi:https://dx.doi.org/10.1002/nur.21753

Kang, K. I., & Joung, J. (2020). Outcomes of Consumer Involvement in Mental Health Nursing Education: An Integrative Review. *International Journal of Environmental Research & Public Health [Electronic Resource], 17*(18), 16. doi:https://dx.doi.org/10.3390/ijerph17186756

Karazivan, P., Dumez, V., Flora, L., Pomey, M. P., Del Grande, C., Ghadiri, D. P., . . . Lebel, P. (2015). The patient-as-partner approach in health care: a conceptual framework for a necessary transition. *Academic Medicine, 90*(4), 437-441. doi:https://dx.doi.org/10.1097/ACM.0000000000000603

Katz, M. L., Archer, L. E., Peppercorn, J. M., Kereakoglow, S., Collyar, D. E., Burstein, H. J., . . . Partridge, A. H. (2012). Patient advocates' role in clinical trials: perspectives from Cancer and Leukemia Group B investigators and advocates. *Cancer, 118*(19), 4801-4805. doi:https://dx.doi.org/10.1002/cncr.27485

Kelly, G., Wang, S. Y., Lucas, G., Fraenkel, L., & Gross, C. P. (2017). Facilitating Meaningful Engagement on Community Advisory Committees in Patient-Centered Outcome Research. *Progress in Community Health Partnerships, 11*(3), 243-251. doi:https://dx.doi.org/10.1353/cpr.2017.0029

Kemper, C., Blackburn, C., Doyle, J. A., & Hyman, D. (2013). Engaging patients and families in system-level improvement: a safety imperative. *Nursing Administration Quarterly, 37*(3), 203-215. doi:https://dx.doi.org/10.1097/NAQ.0b013e318295f61e

Khodyakov, D., Grant, S., Denger, B., Kinnett, K., Martin, A., Peay, H., & Coulter, I. (2020). Practical Considerations in Using Online Modified-Delphi Approaches to Engage Patients and Other Stakeholders in Clinical Practice Guideline Development. *The Patient: Patient-Centered Outcomes Research, 13*(1), 11-21. doi:https://dx.doi.org/10.1007/s40271-019-00389-4

Kidd, S., Kenny, A., & Endacott, R. (2007). Consumer advocate and clinician perceptions of consumer participation in two rural mental health services. *International Journal of Mental Health Nursing, 16*(3), 214-222.

Kimminau, K. S., Jernigan, C., LeMaster, J., Aaronson, L. S., Christopher, M., Ahmed, S., . . . Waitman, L. R. (2018). Patient vs. Community Engagement: Emerging Issues. *Medical Care, 56 Suppl 10 Suppl 1*, S53-S57. doi:https://dx.doi.org/10.1097/MLR.0000000000000772

Kirwan, J. R., Ahlmen, M., de Wit, M., Heiberg, T., Hehir, M., Hewlett, S., . . . Richards, P. (2005). Progress since OMERACT 6 on including patient perspective in rheumatoid arthritis outcome assessment. *Journal of Rheumatology, 32*(11), 2246-2249.

Kirwan, J. R., de Wit, M., Frank, L., Haywood, K. L., Salek, S., Brace-McDonnell, S., . . . Bartlett, S. J. (2017). Emerging Guidelines for Patient Engagement in Research. *Value in Health, 20*(3), 481-486. doi:10.1016/j.jval.2016.10.003

Klein, A. V., Hardy, S., Lim, R., & Marshall, D. A. (2016). Regulatory Decision Making in Canada-Exploring New Frontiers in Patient Involvement. *Value in Health, 19*(6), 730-733. doi:https://dx.doi.org/10.1016/j.jval.2016.03.1855

Knaapen, L., & Lehoux, P. (2016). Three Conceptual Models of Patient and Public Involvement in Standard-setting: From Abstract Principles to Complex Practice. *Science as Culture, 25*(2), 239-263. doi:10.1080/09505431.2015.1125875

Komporozos-Athanasiou, A., Fudge, N., Adams, M., & McKevitt, C. (2018). Citizen Participation as Political Ritual: Towards a Sociological Theorizing of 'Health Citizenship'. *Sociology-the Journal of the British Sociological Association, 52*(4), 744-761. doi:10.1177/0038038516664683

Koniotou, M., Evans, B. A., Chatters, R., Fothergill, R., Garnsworthy, C., Gaze, S., . . . Snooks, H. (2015). Involving older people in a multi-centre randomised trial of a complex intervention in pre-hospital emergency care: implementation of a collaborative model. *Trials [Electronic Resource], 16*, 298. doi:https://dx.doi.org/10.1186/s13063-015-0821-z

Kovacs Burns, K. (2008). Canadian patient safety champions: collaborating on improving patient safety. *Healthcare Quarterly, 11*(3 Spec No.), 95-100.

Kreindler, S. A. (2009). Patient involvement and the politics of methodology. *Canadian Public Administration-Administration Publique Du Canada, 52*(1), 113-124. doi:10.1111/j.1754-7121.2009.00062.x

Kuehn, C. M. (2018). Patient Experience Data in US Food and Drug Administration (FDA) Regulatory Decision Making: A Policy Process Perspective. *Therapeutic Innovation & Regulatory Science, 52*(5), 661-668. doi:10.1177/2168479017753390

Kushner, C., & Davis, D. (2014). Improving safety: engaging with patients and families makes a difference! *Healthcare Quarterly, 17 Spec No*, 41-44.

Kwon, S. C., Tandon, S. D., Islam, N., Riley, L., & Trinh-Shevrin, C. (2018). Applying a community-based participatory research framework to patient and family engagement in the development of patient-centered outcomes research and practice. *Translational Behavioral Medicine, 8*(5), 683-691. doi:10.1093/tbm/ibx026

LaBoube, J., Pruitt, K., George, P. R., Mainda, D., Gregory, W., Allen, B., . . . Klocek, J. (2012). Partners in Change: Bringing People in Recovery into the Process of Evaluating Recovery Oriented Services. *American Journal of Psychiatric Rehabilitation, 15*(3), 255-273. doi:10.1080/15487768.2012.703546

Lammers, J., & Happell, B. (2004). Mental health reforms and their impact on consumer and carer participation: a perspective from Victoria, Australia. *Issues in Mental Health Nursing, 25*(3), 261-276.

Lammers, J., & Happell, B. (2004). Research involving mental health consumers and carers: a reference group approach. *International Journal of Mental Health Nursing, 13*(4), 262-266.

Larsen, T., & Sagvaag, H. (2018). Empowerment and pathologization: A case study in Norwegian mental health and substance abuse services. *Health Expectations, 21*(6), 1231-1240. doi:https://dx.doi.org/10.1111/hex.12828

Lauckner, H., Doucet, S., & Wells, S. (2012). Patients as educators: the challenges and benefits of sharing experiences with students. *Medical Education, 46*(10), 992-1000. doi:https://dx.doi.org/10.1111/j.1365-2923.2012.04356.x

Laurance, J., Henderson, S., Howitt, P. J., Matar, M., Al Kuwari, H., Edgman-Levitan, S., & Darzi, A. (2014). IMPROVING CARE & HEALTH. Patient Engagement: Four Case Studies That Highlight The Potential For Improved Health Outcomes And Reduced Costs. *Health Affairs, 33*(9), 1627-1634. doi:10.1377/hlthaff.2014.0375

Lavallee, D. C., Blakeney, E. A., Yu, Y., Johnson, R., Liner, D. A., Murphy, N. L., . . . Zierler, B. K. (2019). Engaging patients and families to transform heart failure care. *Journal of Interprofessional Care*, 1-4. doi:https://dx.doi.org/10.1080/13561820.2019.1696286

Lavallee, D. C., Blakeney, E. A., Yu, Y., Johnson, R., Liner, D. A., Murphy, N. L., . . . Zierler, B. K. (2020). Engaging patients and families to transform heart failure care. *Journal of Interprofessional Care, 34*(6), 835-838. doi:https://dx.doi.org/10.1080/13561820.2019.1696286

Lavoie-Tremblay, M., O'Connor, P., Biron, A., MacGibbon, B., Cyr, G., & Fréchette, J. (2016). The Experience of Patients Engaged in Co-designing Care Processes. *Health Care Manager, 35*(4), 284-293. doi:10.1097/HCM.0000000000000132

Leese, J., Macdonald, G., Kerr, S., Gulka, L., Hoens, A. M., Lum, W., . . . Li, L. C. (2018). 'Adding another spinning plate to an already busy life'. Benefits and risks in patient partner-researcher relationships: a qualitative study of patient partners' experiences in a Canadian health research setting. *BMJ Open, 8*(8), e022154. doi:https://dx.doi.org/10.1136/bmjopen-2018-022154

Lefebvre, H., Brault, I., Roy, O., Levert, M.-J., Lecocq, D., Larrivière, M., . . . Maddalena, N. (2018). Partenariat entre patients, leaders en soins infirmiers et chercheurs : résultats d'une stratégie web de transfert des connaissances pour planifier le congé d'hôpital et faciliter les transitions au cours des soins oncologiques...Partnership between patients, nurse leaders and researchers: Outcomes of a web-based KT strategy for hospital discharge planning and care transitions in oncology. *Canadian Oncology Nursing Journal, 28*(2), 102-117. doi:10.5737/23688076282102109

Leff, B., Sheehan, O. C., Harrison, K. L., England, A. E., Mickler, A., Basyal, P. S., . . . Ritchie, C. S. A Home-Based Care Research Agenda by and for Homebound Older Adults and Caregivers. *Journal of Applied Gerontology*, 7. doi:10.1177/07334648211004731

Legare, F., Boivin, A., van der Weijden, T., Packenham, C., Tapp, S., & Burgers, J. (2009). A knowledge synthesis of patient and public involvement in clinical practice guidelines: study protocol. *Implementation Science, 4*. doi:10.1186/1748-5908-4-30

LeMaster, J. W. (2020). PPIE in intervention studies: Randomized trials and clinical quality improvement. *Health Expectations, 23*(1), 1-2. doi:https://dx.doi.org/10.1111/hex.13009

L'Esperance, A., O'Brien, N., Gregoire, A., Abelson, J., Canfield, C., Del Grande, C., . . . Boivin, A. (2021). Developing a Canadian evaluation framework for patient and public engagement in research: study protocol. *Research Involvement & Engagement, 7*(1), 10. doi:https://dx.doi.org/10.1186/s40900-021-00255-4

Lessard, D., Engler, K., Vicente, S., Bilodeau, M., & Lebouche, B. (2020). Challenges of Patient Engagement in an HIV Clinical Research Program: A Qualitative Analysis of Stakeholder Accounts. *Journal of Patient Experience, 7*(6), 925-930. doi:https://dx.doi.org/10.1177/2374373520975728

Lindblom, S., Flink, M., Elf, M., Laska, A. C., von Koch, L., & Ytterberg, C. The manifestation of participation within a co-design process involving patients, significant others and health-care professionals. *Health Expectations*, 12. doi:10.1111/hex.13233

Linhorst, D. M., Eckert, A., Hamilton, G., & Young, E. (2001). The involvement of a consumer council in organizational decision making in a public psychiatric hospital. *Journal of Behavioral Health Services & Research, 28*(4), 427-438.

Livingston, J. D., Nijdam-Jones, A., Lapsley, S., Calderwood, C., & Brink, J. (2013). Supporting recovery by improving patient engagement in a forensic mental health hospital: results from a demonstration project. *Journal of the American Psychiatric Nurses Association, 19*(3), 132-145. doi:https://dx.doi.org/10.1177/1078390313489730

Lock, M. J., Thomas, D. P., Anderson, I. P., & Pattison, P. (2011). Indigenous participation in an informal national indigenous health policy network. *Australian Health Review, 35*(3), 309-315. doi:https://dx.doi.org/10.1071/AH09812

Lopatina, E., Marshall, D. A., Barber, C. E. H., Miller, J. L., Teare, S. R., Marlett, N. J., . . . Woodhouse, L. J. (2019). The voice of patients in system redesign: A case study of redesigning a centralized system for intake of referrals from primary care to rheumatologists for patients with suspected rheumatoid arthritis. *Health Expectations, 22*(3), 348-363. doi:10.1111/hex.12855

Lopes, E., Carter, D., & Street, J. (2015). Power relations and contrasting conceptions of evidence in patient-involvement processes used to inform health funding decisions in Australia. *Social Science & Medicine, 135*, 84-91. doi:https://dx.doi.org/10.1016/j.socscimed.2015.04.021

Loud, F., Jain, N., & Thomas, N. (2013). How to develop a patient and carer advisory group in a quality improvement study. *Journal of Renal Care, 39 Suppl 2*, 2-9. doi:https://dx.doi.org/10.1111/j.1755-6686.2013.12032.x

Ludwig, C., Graham, I. D., Gifford, W., Lavoie, J., & Stacey, D. (2020). Partnering with frail or seriously ill patients in research: a systematic review. *Research Involvement & Engagement, 6*, 52. doi:https://dx.doi.org/10.1186/s40900-020-00225-2

Macarthur, C., Walsh, C. M., Buchanan, F., Karoly, A., Pires, L., McCreath, G., & Jones, N. L. (2021). Development of the patient-oriented research curriculum in child health (PORCCH). *Research Involvement & Engagement, 7*(1), 27. doi:https://dx.doi.org/10.1186/s40900-021-00276-z

MacSweeney, N., Bowman, S., & Kelly, C. (2019). More than just characters in a story: effective and meaningful involvement of young people in mental health research. *Journal of Public Mental Health, 18*(1), 14-16. doi:10.1108/JPMH-07-2018-0053

Mader, L. B., Harris, T., Klager, S., Wilkinson, I. B., & Hiemstra, T. F. (2018). Inverting the patient involvement paradigm: defining patient led research. *Research Involvement & Engagement, 4*, 21. doi:https://dx.doi.org/10.1186/s40900-018-0104-4

Majid, U., & Gagliardi, A. (2019). Clarifying the degrees, modes, and muddles of "meaningful" patient engagement in health services planning and designing. *Patient Education and Counseling, 102*(9), 1581-1589. doi:10.1016/j.pec.2019.04.006

Malfait, S., Van Hecke, A., De Bodt, G., Palsterman, N., & Eeckloo, K. (2018). Patient and public involvement in hospital policy-making: Identifying key elements for effective participation. *Health Policy, 122*(4), 380-388. doi:https://dx.doi.org/10.1016/j.healthpol.2018.02.007

Malterud, K., & Elvbakken, K. T. (2020). Patients participating as co-researchers in health research: A systematic review of outcomes and experiences. *Scandinavian Journal of Public Health, 48*(6), 617-628. doi:https://dx.doi.org/10.1177/1403494819863514

Mamzer, M. F., Duchange, N., Darquy, S., Marvanne, P., Rambaud, C., Marsico, G., . . . Herve, C. (2017). Partnering with patients in translational oncology research: ethical approach. *Journal of Translational Medicine, 15*(1), 74. doi:https://dx.doi.org/10.1186/s12967-017-1177-9

Manafo, E., Petermann, L., Mason-Lai, P., & Vandall-Walker, V. (2018). Patient engagement in Canada: a scoping review of the 'how' and 'what' of patient engagement in health research. *Health Research Policy & Systems, 16*(1), 5. doi:https://dx.doi.org/10.1186/s12961-018-0282-4

Mandel, L. A., & Qazilbash, J. (2005). Youth voices as change agents: moving beyond the medical model in school-based health center practice. *Journal of School Health, 75*(7), 239-242.

Markle-Reid, M., Ganann, R., Ploeg, J., Heald-Taylor, G., Kennedy, L., McAiney, C., & Valaitis, R. (2021). Engagement of older adults with multimorbidity as patient research partners: Lessons from a patient-oriented research program. *Journal of Comorbidity, 11*, 2633556521999508. doi:https://dx.doi.org/10.1177/2633556521999508

Martini, J., Tijou Traore, A., & Mahieu, C. (2019). Chronic patient as intermittent partner for policy-makers: the case of patient participation in the fight against diabetes and HIV/AIDS in Mali. *Bmc Public Health, 19*(1), 1179. doi:https://dx.doi.org/10.1186/s12889-019-7453-2

Mathie, E., Wythe, H., Munday, D., Millac, P., Rhodes, G., Roberts, N., . . . Jones, J. (2018). Reciprocal relationships and the importance of feedback in patient and public involvement: A mixed methods study. *Health Expectations, 21*(5), 899-908. doi:https://dx.doi.org/10.1111/hex.12684

McCarron, T. L., Clement, F., Rasiah, J., Moffat, K., Wasylak, T., & Santana, M. J. (2021). Co-designing strategies to support patient partners during a scoping review and reflections on the process: a commentary. *Research Involvement & Engagement, 7*(1), 25. doi:https://dx.doi.org/10.1186/s40900-021-00272-3

McClean, J., & Trigger, K. (2017). Not just tea and biscuits; the Gold Coast Primary Health Network process of designing, implementing and operating a Community Advisory Council. *Australian Journal of Primary Health, 23*(6), 504-508. doi:https://dx.doi.org/10.1071/PY16157

McDaid, S. (2009). An equality of condition framework for user involvement in mental health policy and planning: evidence from participatory action research. *Disability & Society, 24*(4), 461-474. Retrieved from http://libaccess.mcmaster.ca/login?url=http://search.ebscohost.com/login.aspx?direct=true&db=cin20&AN=105543573&site=ehost-live&scope=site

McGrady, M. E., Norris, R. E., & Pai, A. L. H. (2018). Collaborating with Adolescents and Young Adults with Cancer as Advisors. *Journal of Adolescent & Young Adult Oncology, 7*(4), 499-503. doi:https://dx.doi.org/10.1089/jayao.2018.0013

McMillan, F. V., Browne, N., Green, S., & Donnelly, D. (2009). A card before you leave: participation and mental health in Northern Ireland. *Health & Human Rights, 11*(1), 61-72.

Melchior, I., van der Heijden, A., Stoffers, E., Suntjens, F., & Moser, A. (2021). Patient and public involvement cultures and the perceived impact in the vulnerable context of palliative care: A qualitative study. *Health Expectations, 24*(2), 456-467. doi:https://dx.doi.org/10.1111/hex.13186

Mercer, R. E., Chambers, A., Mai, H., McDonald, V., McMahon, C., & Chan, K. K. W. (2020). Are We Making a Difference? A Qualitative Study of Patient Engagement at the pan-Canadian Oncology Drug Review: Perspectives of Patient Groups. *Value in Health, 23*(9), 1157-1162. doi:https://dx.doi.org/10.1016/j.jval.2020.06.003

Merkel, P. A., Manion, M., Gopal-Srivastava, R., Groft, S., Jinnah, H. A., Robertson, D., . . . Rare Diseases Clinical Research, N. (2016). The partnership of patient advocacy groups and clinical investigators in the rare diseases clinical research network. *Orphanet Journal Of Rare Diseases, 11*(1), 66. doi:https://dx.doi.org/10.1186/s13023-016-0445-8

Mescouto, K., & Setchell, J. (2020). Patients as Partners in Research: A Practical Example of How Researchers Are Contributing to the Patient-Partnership Revolution. *Journal of Orthopaedic & Sports Physical Therapy, 50*(7), 347-349. doi:https://dx.doi.org/10.2519/jospt.2020.0105

Miller, C., Pradeep, V., Mohamad, M., Izmeth, Z., Reynolds, M. T. P., & Gulati, G. (2020). Patients and carers as teachers in psychiatric education: a literature review and discussion. *Irish Journal of Psychological Medicine, 37*(2), 126-133. doi:10.1017/ipm.2016.45

Mjøsund, N. H., Eriksson, M., Espnes, G. A., Haaland‐Øverby, M., Jensen, S. L., Norheim, I., . . . Vinje, H. F. (2017). Service user involvement enhanced the research quality in a study using interpretative phenomenological analysis - the power of multiple perspectives. *Journal of Advanced Nursing (John Wiley & Sons, Inc.), 73*(1), 265-278. doi:10.1111/jan.13093

Morris, R. L., Ruddock, A., Gallacher, K., Rolfe, C., Giles, S., & Campbell, S. (2021). Developing a patient safety guide for primary care: A co-design approach involving patients, carers and clinicians. *Health Expectations, 24*(1), 42-52. doi:https://dx.doi.org/10.1111/hex.13143

Moser, A., Melchior, I., Veenstra, M., Stoffers, E., Derks, E., & Jie, K. S. (2021). Improving the experience of older people with colorectal and breast cancer in patient‐centred cancer care pathways using experience‐based co‐design. *Health Expectations, 24*(2), 478-490. doi:10.1111/hex.13189

Moulon, I., & Dedes, N. (2010). The patients' and consumers' working party at the European Medicines Agency: a model of interaction between patients, consumers, and medicines regulatory authorities. *Journal of Ambulatory Care Management, 33*(3), 190-197. doi:https://dx.doi.org/10.1097/JAC.0b013e3181e59322

Mwinga, A., & Moodley, K. (2015). Engaging with Community Advisory Boards (CABs) in Lusaka Zambia: perspectives from the research team and CAB members. *BMC Medical Ethics, 16*, 39. doi:https://dx.doi.org/10.1186/s12910-015-0031-y

Nambisan, P., & Nambisan, S. (2009). Models of consumer value cocreation in health care. *Health Care Management Review, 34*(4), 344-354. doi:https://dx.doi.org/10.1097/HMR.0b013e3181abd528

Nancarrow, S., Johns, A., & Vernon, W. (2004). 'The squeaky wheel gets the grease': a case study of service user engagement in service development. *Journal of Integrated Care, 12*(6), 14-21. Retrieved from http://libaccess.mcmaster.ca/login?url=http://search.ebscohost.com/login.aspx?direct=true&db=cin20&AN=106549828&site=ehost-live&scope=site

Naqshbandi Hayward, M., Paquette-Warren, J., Harris, S. B., & Team, F. A. P. (2016). Developing community-driven quality improvement initiatives to enhance chronic disease care in Indigenous communities in Canada: the FORGE AHEAD program protocol. *Health Research Policy & Systems, 14*(1), 55. doi:https://dx.doi.org/10.1186/s12961-016-0127-y

Nathan, S., Stephenson, N., & Braithwaite, J. (2014). Sidestepping questions of legitimacy: how community representatives manoeuvre to effect change in a health service. *Health: an Interdisciplinary Journal for the Social Study of Health, Illness & Medicine, 18*(1), 23-40. doi:https://dx.doi.org/10.1177/1363459312473617

Needham, J., Taylor, J., & Nomikos, D. (2021). Integrating Patient-Centred Research in the Canadian Cancer Trials Group. *Current Oncology, 28*(1), 630-639. doi:https://dx.doi.org/10.3390/curroncol28010062

Nelson, G., Macnaughton, E., Curwood, S. E., Egalité, N., Voronka, J., Fleury, M. J., . . . Goering, P. (2016). Collaboration and involvement of persons with lived experience in planning Canada's At Home/ Chez Soi project. *Health & Social Care in the Community, 24*(2), 184-193. doi:10.1111/hsc.12197

Nguyen-Truong, C. K. Y., Fritz, R. L., Junghee, L., Lau, C., Cang, L., Kim, J., . . . Van Son, C. (2018). Interactive CO-learning for Research Engagement and Education (I-COREE) Curriculum to Build Capacity Between Community Partners and Academic Researchers. *Asian Pacific Island Nursing Journal, 3*(4), 126-138. Retrieved from http://libaccess.mcmaster.ca/login?url=http://search.ebscohost.com/login.aspx?direct=true&db=cin20&AN=134691572&site=ehost-live&scope=site

Nierse, C. J., Schipper, K., van Zadelhoff, E., van de Griendt, J., & Abma, T. A. (2012). Collaboration and co-ownership in research: dynamics and dialogues between patient research partners and professional researchers in a research team. *Health Expectations, 15*(3), 242-254. doi:https://dx.doi.org/10.1111/j.1369-7625.2011.00661.x

Nikiphorou, E., Alunno, A., Carmona, L., Kouloumas, M., Bijlsma, J., & Cutolo, M. (2017). Patient-physician collaboration in rheumatology: a necessity. *RMD Open, 3*(1), e000499. doi:https://dx.doi.org/10.1136/rmdopen-2017-000499

Norburn, L., & Thomas, L. (2020). Expertise, experience, and excellence. Twenty years of patient involvement in health technology assessment at NICE: an evolving story. *International Journal of Technology Assessment in Health Care, 37*, e15. doi:https://dx.doi.org/10.1017/S0266462320000860

Nyirenda, D., Sariola, S., Gooding, K., Phiri, M., Sambakunsi, R., Moyo, E., . . . Desmond, N. (2018). 'We are the eyes and ears of researchers and community': Understanding the role of community advisory groups in representing researchers and communities in Malawi. *Developing World Bioethics, 18*(4), 420-428. doi:https://dx.doi.org/10.1111/dewb.12163

Ochocka, J., Janzen, R., & Nelson, G. (2002). Sharing power and knowledge: professional and mental health consumer/survivor researchers working together in a participatory action research project. *Psychiatric Rehabilitation Journal, 25*(4), 379-387.

O'Donnell, D., She, E. N., McCarthy, M., Thornton, S., Doran, T., Smith, F., . . . Marie, T. C. (2019). Enabling public, patient and practitioner involvement in co-designing frailty pathways in the acute care setting. *Bmc Health Services Research, 19*(1), 11. doi:10.1186/s12913-019-4626-8

Okun, S., & Goodwin, K. (2017). Building a learning health community: By the people, for the people. *Learning Health Systems, 1*(3), e10028. doi:https://dx.doi.org/10.1002/lrh2.10028

Oldfield, B. J., Harrison, M. A., Genao, I., Greene, A. T., Pappas, M. E., Glover, J. G., & Rosenthal, M. S. (2019). Patient, Family, and Community Advisory Councils in Health Care and Research: a Systematic Review. *Journal of General Internal Medicine, 34*(7), 1292-1303. doi:10.1007/s11606-018-4565-9

Oliver, S., Armes, D. G., & Gyte, G. (2009). Public Involvement in Setting a National Research Agenda A Mixed Methods Evaluation. *Patient-Patient Centered Outcomes Research, 2*(3), 179-190. doi:10.2165/11314860-000000000-00000

Opava, C. H., & Carlsson, A. (2012). The role of patient organisations in musculoskeletal care. *Best Practice & Research in Clinical Rheumatology, 26*(3), 399-407. doi:https://dx.doi.org/10.1016/j.berh.2012.05.003

Oxland, P., Foster, N., Fiest, K. M., & Skrobik, Y. (2020). Engaging Patients and Families to Help Research Inform and Advance Patient and Family-Centered Care in Critical Care Medicine. *Critical Care Nursing Clinics of North America, 32*(2), 211-226. doi:https://dx.doi.org/10.1016/j.cnc.2020.02.004

Pagura, S. M. C., Oxenham-Murphy, L., Savage, D., Zarem, A., & Khan, A. (2018). Untapped Potential: Engaging in Meaningful Client and Family Partnerships to Drive High-Quality, Safe Care. *Healthcare Quarterly, 21*(2), 30-34. doi:https://dx.doi.org/10.12927/hcq.2018.25625

Parry, M., Bjornnes, A. K., Toupin-April, K., Najam, A., Wells, D., Sivakumar, A., . . . Marlin, S. (2020). Patient Engagement Partnerships in Clinical Trials: Development of Patient Partner and Investigator Decision Aids. *The Patient: Patient-Centered Outcomes Research, 13*(6), 745-756. doi:https://dx.doi.org/10.1007/s40271-020-00460-5

Patterson, S., Trite, J., & Weaver, T. (2014). Activity and views of service users involved in mental health research: UK survey. *British Journal of Psychiatry, 205*(1), 68-75. doi:https://dx.doi.org/10.1192/bjp.bp.113.128637

Paxton, S., & Stephens, D. (2007). Challenges to the meaningful involvement of HIV-positive people in the response to HIV/AIDS in Cambodia, India and Indonesia. *Asia-Pacific Journal of Public Health, 19*(1), 8-13.

Peikes, D., O'Malley, A. S., Wilson, C., Crosson, J., Gaddes, R., Natzke, B., . . . Ralston, J. (2016). Early Experiences Engaging Patients Through Patient and Family Advisory Councils. *Journal of Ambulatory Care Management, 39*(4), 316-324. doi:https://dx.doi.org/10.1097/JAC.0000000000000150

Pelletier, J. F., Lesage, A., Boisvert, C., Denis, F., Bonin, J. P., & Kisely, S. (2015). Feasibility and acceptability of patient partnership to improve access to primary care for the physical health of patients with severe mental illnesses: an interactive guide. *International Journal for Equity in Health, 14*, 78. doi:https://dx.doi.org/10.1186/s12939-015-0200-0

Perfetto, E. M., Burke, L., Oehrlein, E. M., & Epstein, R. S. (2015). Patient-Focused Drug Development: A New Direction for Collaboration. *Medical Care, 53*(1), 9-17. doi:https://dx.doi.org/10.1097/MLR.0000000000000273

Perlmutter, J., Roach, N., & Smith, M. L. (2015). Involving Advocates in Cancer Research. *Seminars in Oncology, 42*(5), 681-685. doi:https://dx.doi.org/10.1053/j.seminoncol.2015.07.008

Petit-Zeman, S., Philpots, E., & Denegri, S. (2010). "Natural ground" for medical research charities: public and patient involvement in research funding. *Journal of Ambulatory Care Management, 33*(3), 249-256. doi:https://dx.doi.org/10.1097/JAC.0b013e3181e5d279

Pflugeisen, B. M., Patterson, P., Macpherson, C. F., Ray, B. C., Jacobsen, R. L., Hornyak, N., & Johnson, R. H. (2019). Putting Adolescents and Young Adults in a Room Together: Launching an Adolescent and Young Adult Oncology Council. *Journal of Adolescent & Young Adult Oncology, 8*(5), 540-546. doi:10.1089/jayao.2018.0139

Phelps, D. (2017). The Voices of Young Carers in Policy and Practice. *Social Inclusion, 5*(3), 113-121. doi:10.17645/si.v5i3.965

Phoenix, M., Nguyen, T., Gentles, S. J., VanderKaay, S., Cross, A., & Nguyen, L. (2018). Using qualitative research perspectives to inform patient engagement in research. *Research Involvement & Engagement, 4*, 20. doi:https://dx.doi.org/10.1186/s40900-018-0107-1

Pickles, J., Hide, E., & Maher, L. (2008). Experience based design: a practical method of working with patients to redesign services. *Clinical Governance: An International Journal, 13*(1), 51-58. Retrieved from http://libaccess.mcmaster.ca/login?url=http://search.ebscohost.com/login.aspx?direct=true&db=cin20&AN=105727758&site=ehost-live&scope=site

Pii, K. H., Schou, L. H., Piil, K., & Jarden, M. (2019). Current trends in patient and public involvement in cancer research: A systematic review. *Health Expectations, 22*(1), 3-20. doi:10.1111/hex.12841

Poleshuck, E., Wittink, M., Crean, H., Gellasch, T., Sandler, M., Bell, E., . . . Cerulli, C. (2015). Using patient engagement in the design and rationale of a trial for women with depression in obstetrics and gynecology practices. *Contemporary Clinical Trials, 43*, 83-92. doi:https://dx.doi.org/10.1016/j.cct.2015.04.010

Pollard, K., Donskoy, A. L., Moule, P., Donald, C., Lima, M., & Rice, C. (2015). Developing and evaluating guidelines for patient and public involvement (PPI) in research. *International Journal of Health Care Quality Assurance, 28*(2), 141-155. doi:https://dx.doi.org/10.1108/IJHCQA-01-2014-0001

Pomey, M. P., Bush, P. L., Demers-Payette, O., L'Esperance, A., Lochhead, L., Ganache, I., & Roy, D. (2020). Developing recommendations for the diagnosis and treatment of Lyme disease: the role of the patient's perspective in a controversial environment. *International Journal of Technology Assessment in Health Care, 37*, e11. doi:https://dx.doi.org/10.1017/S0266462320002123

Pomey, M. P., & Lebel, P. (2016). Patient Engagement: The Quebec Path. *Healthcarepapers, 16*(2), 78-83.

Pomey, M.-P., Lebel, P., Clavel, N., Morin, É., Morin, M., Neault, C., . . . Mulliez, A.-P. E. (2018). Development of Patient-Inclusive Teams: Toward a Structured Methodology. *Healthcare Quarterly, 21*, 38-44. Retrieved from http://libaccess.mcmaster.ca/login?url=http://search.ebscohost.com/login.aspx?direct=true&db=cin20&AN=133597660&site=ehost-live&scope=site

Portalupi, L. B., Lewis, C. L., Miller, C. D., Whiteman-Jones, K. L., Sather, K. A., Nease, D. E., Jr., & Matlock, D. D. (2017). Developing a patient and family research advisory panel to include people with significant disease, multimorbidity and advanced age. *Family Practice, 34*(3), 364-369. doi:https://dx.doi.org/10.1093/fampra/cmw138

Potter, D. A. (2010). 'Wrong parents' and 'right parents': shared perspectives about citizen participation in policy implementation. *Social Science & Medicine, 70*(11), 1705-1713. doi:https://dx.doi.org/10.1016/j.socscimed.2010.01.025

Preston, J. L., Berryman, V. R., Hancock, A., Pattrick, M., Worthington, A., Hitman, G. A., & Hood, G. A. (2019). Developing patient and public involvement and engagement (PPIE) in diabetes research: a local approach. *Practical Diabetes, 36*(3), 81-85. doi:10.1002/pdi.2220

Price, A., Albarqouni, L., Kirkpatrick, J., Clarke, M., Liew, S. M., Roberts, N., & Burls, A. (2018). Patient and public involvement in the design of clinical trials: An overview of systematic reviews. *Journal of Evaluation in Clinical Practice, 24*(1), 240-253. doi:https://dx.doi.org/10.1111/jep.12805

Quennell, P. (2001). Getting their say, or getting their way? Has participation strengthened the patient "voice" in the National Institute for Clinical Excellence? *Journal of Management in Medicine, 15*(3), 202-219.

Quennell, P. (2003). Getting a word in edgeways? Patient group participation in the appraisal process of the National Institute for Clinical Excellence. *Clinical Governance: An International Journal, 8*(1), 39-45. Retrieved from http://libaccess.mcmaster.ca/login?url=http://search.ebscohost.com/login.aspx?direct=true&db=cin20&AN=106844965&site=ehost-live&scope=site

Rabeharisoa, V. (2003). The struggle against neuromuscular diseases in France and the emergence of the "partnership model" of patient organisation. *Social Science & Medicine, 57*(11), 2127-2136.

Rae, S. (2017). Service Users perspectives in PROMISE and research. *Psychiatria Danubina, 29*(Suppl 3), 490-494.

Ramazani, S., Bayer, N. D., Gottfried, J. A., Wagner, J., Leonard, M. S., Lynn, J., & Schriefer, J. (2020). The Value of Family Advisors as Coleaders in Pediatric Quality Improvement Efforts: A Qualitative Theme Analysis. *Journal of Patient Experience, 7*(6), 1708-1714. doi:https://dx.doi.org/10.1177/2374373520939827

Rashid, A., Thomas, V., Shaw, T., & Leng, G. (2017). Patient and Public Involvement in the Development of Healthcare Guidance: An Overview of Current Methods and Future Challenges. *The Patient: Patient-Centered Outcomes Research, 10*(3), 277-282. doi:https://dx.doi.org/10.1007/s40271-016-0206-8

Reeve, P., Cornell, S., D'Costa, B., Janzen, R., & Ochocka, J. (2002). From our perspective: consumer researchers speak abut their experience in a community mental health research project. *Psychiatric Rehabilitation Journal, 25*(4), 403-408.

Renedo, A., & Marston, C. (2011). Healthcare professionals' representations of 'patient and public involvement' and creation of 'public participant' identities: Implications for the development of inclusive and bottom-up community participation initiatives. *Journal of Community & Applied Social Psychology, 21*(3), 268-280. doi:10.1002/casp.1092

Renedo, A., & Marston, C. (2015). Developing patient-centred care: an ethnographic study of patient perceptions and influence on quality improvement. *BMC Health Services Research, 15*, 122. doi:https://dx.doi.org/10.1186/s12913-015-0770-y

Renedo, A., Marston, C. A., Spyridonidis, D., & Barlow, J. (2015). Patient and Public Involvement in Healthcare Quality Improvement: How organizations can help patients and professionals to collaborate. *Public Management Review, 17*(1), 17-34. doi:10.1080/14719037.2014.881535

Repper, J., Perkins, R., & Network, U. F. M. (2006). Looking through users' eyes. *Mental Health Today*, 25-28.

Rhodes, P., Nocon, A., Wright, J., & Harrison, S. (2001). Involving patients in research: setting up a service users' advisory group. *Journal of Management in Medicine, 15*(2), 167-171.

Robbins, M., Tufte, J., & Hsu, C. (2016). Learning to "Swim" with the Experts: Experiences of Two Patient Co-Investigators for a Project Funded by the Patient-Centered Outcomes Research Institute. *Permanente Journal, 20*(2), 85-88. doi:https://dx.doi.org/10.7812/TPP/15-162

Rose, D., MacDonald, D., Wilson, A., Crawford, M., Barnes, M., & Omeni, E. (2016). Service user led organisations in mental health today. *Journal of Mental Health, 25*(3), 254-259. doi:https://dx.doi.org/10.3109/09638237.2016.1139070

Roth, D. (2011). A third seat at the table: an insider's perspective on patient representatives. *Hastings Center Report, 41*(1), 29-31. Retrieved from https://onlinelibrary.wiley.com/doi/abs/10.1002/j.1552-146X.2011.tb00097.x?sid=nlm%3Apubmed

Roy, C. M., & Cain, R. (2001). The involvement of people living with HIV/AIDS in community-based organizations: contributions and constraints. *AIDS Care, 13*(4), 421-432.

Sage, L., Russo, M. L., Byers, P. H., Demasi, J., Morris, S. A., Puryear, L. N., . . . Vascular Ehlers-Danlos Syndrome Research, C. (2020). Setting a research agenda for vascular Ehlers-Danlos syndrome using a patient and stakeholder engagement model. *Journal of Vascular Surgery, 72*(4), 1436-1444.e1432. doi:https://dx.doi.org/10.1016/j.jvs.2019.12.043

Sandvin Olsson, A. B., Strom, A., Haaland-Overby, M., Fredriksen, K., & Stenberg, U. (2020). How can we describe impact of adult patient participation in health-service development? A scoping review. *Patient Education & Counseling, 103*(8), 1453-1466. doi:https://dx.doi.org/10.1016/j.pec.2020.02.028

Sangill, C., Buus, N., Hybholt, L., & Berring, L. L. (2019). Service user's actual involvement in mental health research practices: A scoping review. *International Journal of Mental Health Nursing, 28*(4), 798-815. doi:https://dx.doi.org/10.1111/inm.12594

Saunders, C., & Girgis, A. (2011). Enriching health research through consumer involvement--learning through atypical exemplars. *Health Promotion Journal of Australia, 22*(3), 196-202.

Sbaih, L., & Hackin, J. (2002). Working with patients: developing a service user group in one A&E department. *Accident & Emergency Nursing, 10*(1), 26-30.

Schlaudecker, J. D., & Goodnow, K. (2021). The Virtual Patient and Family Advisory Council in the COVID-19 Era. *Journal of the American Board of Family Medicine: JABFM, 34*(Suppl), S37-S39. doi:https://dx.doi.org/10.3122/jabfm.2021.S1.200449

Scholz, B., Bocking, J., & Happell, B. (2017). How do consumer leaders co-create value in mental health organisations? *Australian Health Review, 41*(5), 505-510. doi:https://dx.doi.org/10.1071/AH16105

Scholz, B., Bocking, J., & Happell, B. (2018). Improving exchange with consumers within mental health organizations: Recognizing mental ill health experience as a 'sneaky, special degree'. *International Journal of Mental Health Nursing, 27*(1), 227-235. doi:https://dx.doi.org/10.1111/inm.12312

Scholz, B., Bocking, J., Hedt, P., Lu, V. N., & Happell, B. (2020). 'Not in the room, but the doctors were': an Australian story-completion study about consumer representation. *Health Promotion International, 35*(4), 752-761. doi:https://dx.doi.org/10.1093/heapro/daz070

Scholz, B., Bocking, J., Platania-Phung, C., Banfield, M., & Happell, B. (2018). "Not an afterthought": Power imbalances in systemic partnerships between health service providers and consumers in a hospital setting. *Health Policy, 122*(8), 922-928. doi:https://dx.doi.org/10.1016/j.healthpol.2018.06.007

Scholz, B., Gordon, S., & Happell, B. (2017). Consumers in mental health service leadership: A systematic review. *International Journal of Mental Health Nursing, 26*(1), 20-31. doi:https://dx.doi.org/10.1111/inm.12266

Scholz, B., Roper, C., Juntanamalaga, P., & Happell, B. (2019). Understanding the Role of Allies in Systemic Consumer Empowerment: A Literature Review. *Issues in Mental Health Nursing, 40*(4), 354-361. doi:https://dx.doi.org/10.1080/01612840.2018.1553004

Selig, W., Banks, I., Davis, A., DeCotiis, G., Hohman, R., & Schlager, L. (2019). Incorporating Patient Advocates in Oncology Clinical Development: Lessons Learned From a Novel Pilot Program. *Therapeutic Innovation & Regulatory Science, 53*(3), 349-353. doi:https://dx.doi.org/10.1177/2168479018790533

Sharma, A., Angel, L., & Bui, Q. (2015). Patient Advisory Councils: Giving Patients a Seat at the Table. *Family Practice Management, 22*(4), 22-27. Retrieved from https://www.aafp.org/fpm/2015/0700/p22.pdf

Sharma, A. E., Huang, B., Knox, M., Willard-Grace, R., & Potter, M. B. (2018). Patient Engagement in Community Health Center Leadership: How Does it Happen? *Journal of Community Health, 43*(6), 1069-1074. doi:https://dx.doi.org/10.1007/s10900-018-0523-z

Sharma, A. E., Knox, M., Mleczko, V. L., & Olayiwola, J. N. (2017). The impact of patient advisors on healthcare outcomes: a systematic review. *BMC Health Services Research, 17*(1), 693. doi:https://dx.doi.org/10.1186/s12913-017-2630-4

Sharma Mahendra, V., Ranauta, A., Yuvraj, A., Santella, A. J., Taslim, A., & Doughty, J. (2020). The role of patient and public involvement in oral health and HIV/AIDS research, practice and policy. *Oral Diseases, 26 Suppl 1*, 117-122. doi:https://dx.doi.org/10.1111/odi.13584

Shea, B., Santesso, N., Qualman, A., Heiberg, T., Leong, A., Judd, M., . . . Cochrane Musculoskeletal Consumer, G. (2005). Consumer-driven health care: building partnerships in research. *Health Expectations, 8*(4), 352-359.

Sheikhan, N. Y., Hawke, L. D., Cleverley, K., Darnay, K., Courey, L., Szatmari, P., . . . Henderson, J. (2021). 'It reshaped how I will do research': A qualitative exploration of team members' experiences with youth and family engagement in a randomized controlled trial. *Health Expectations, 24*(2), 589-600. doi:https://dx.doi.org/10.1111/hex.13206

Shen, S., Doyle-Thomas, K. A. R., Beesley, L., Karmali, A., Williams, L., Tanel, N., & McPherson, A. C. (2017). How and why should we engage parents as co-researchers in health research? A scoping review of current practices. *Health Expectations, 20*(4), 543-554. doi:https://dx.doi.org/10.1111/hex.12490

Shklarov, S., Marshall, D. A., Wasylak, T., & Marlett, N. J. (2017). "Part of the Team": Mapping the outcomes of training patients for new roles in health research and planning. *Health Expectations, 20*(6), 1428-1436. doi:https://dx.doi.org/10.1111/hex.12591

Skovlund, P. C., Nielsen, B. K., Thaysen, H. V., Schmidt, H., Finset, A., Hansen, K. A., & Lomborg, K. (2020). The impact of patient involvement in research: a case study of the planning, conduct and dissemination of a clinical, controlled trial. *Research Involvement & Engagement, 6*, 43. doi:https://dx.doi.org/10.1186/s40900-020-00214-5

Smith, E., Donovan, S., Beresford, P., Manthorpe, J., Brearley, S., Sitzia, J., & Ross, F. (2009). Getting ready for user involvement in a systematic review. *Health Expectations, 12*(2), 197-208. doi:https://dx.doi.org/10.1111/j.1369-7625.2009.00535.x

Smith, G. (2000). Consumers' experiences of mental health policymaking. *New Directions for Mental Health Services*(85), 95-103.

Smith, G. (2014). *A qualitative study to investigate service user experience of participating in research.* (M.A.). University of Central Lancashire (United Kingdom), Retrieved from http://libaccess.mcmaster.ca/login?url=http://search.ebscohost.com/login.aspx?direct=true&db=cin20&AN=109786470&site=ehost-live&scope=site Available from EBSCOhost cin20 database.

Smith, K. (2017). The evolving role of people with MS in clinical research-Some progress but more is needed. *Multiple Sclerosis, 23*(12), 1579-1582. doi:https://dx.doi.org/10.1177/1352458517729459

Smith, S., Abbas, M., & Zegarra, A. (2020). Overcoming challenges in service user involvement in an older people's mental health service. *Mental Health & Social Inclusion, 24*(3), 151-155. doi:10.1108/MHSI-04-2020-0016

Solomon, R., Smith, C., Kallio, J., Fenollosa, A., Benerofe, B., Jones, L., . . . Bickell, N. A. (2017). Speaking Up: How Patient and Physician Voices Shaped a Trial to Improve Goals-of-Care Discussions. *Patient-Patient Centered Outcomes Research, 10*(4), 489-501. doi:10.1007/s40271-017-0226-z

Souliotis, K., Peppou, L. E., Agapidaki, E., Tzavara, C., Debiais, D., Hasurdjiev, S., & Sarkozy, F. (2018). Health democracy in Europe: Cancer patient organization participation in health policy. *Health Expectations, 21*(2), 474-484. doi:https://dx.doi.org/10.1111/hex.12638

Souliotis, K., Peppou, L. E., Tzavara, C., Agapidaki, E., Varvaras, D., Buonomo, O., . . . Sarkozy, F. (2018). Cancer patients' organisation participation in heath policy decision-making: a snapshot/cluster analysis of the EU-28 countries. *BMJ Open, 8*(8), e018896. doi:https://dx.doi.org/10.1136/bmjopen-2017-018896

South, A., Hanley, B., Gafos, M., Cromarty, B., Stephens, R., Sturgeon, K., . . . Vale, C. L. (2016). Models and impact of patient and public involvement in studies carried out by the Medical Research Council Clinical Trials Unit at University College London: findings from ten case studies. *Trials [Electronic Resource], 17*, 376. doi:https://dx.doi.org/10.1186/s13063-016-1488-9

Speers, J., & Lathlean, J. (2015). Service user involvement in giving mental health students feedback on placement: A participatory action research study. *Nurse Education Today, 35*(9), e84-89. doi:https://dx.doi.org/10.1016/j.nedt.2015.07.004

Squire, S., Greco, M., O'Hagan, B., Dickinson, K., & Wall, D. (2006). Being patient-centred: creating health care for our grandchildren. *Clinical Governance: An International Journal, 11*(1), 8-16. Retrieved from http://libaccess.mcmaster.ca/login?url=http://search.ebscohost.com/login.aspx?direct=true&db=cin20&AN=106312851&site=ehost-live&scope=site

Squire, S., & Hill, P. (2006). The expert patients programme. *Clinical Governance: An International Journal, 11*(1), 17-21. Retrieved from http://libaccess.mcmaster.ca/login?url=http://search.ebscohost.com/login.aspx?direct=true&db=cin20&AN=106312850&site=ehost-live&scope=site

Staniszewska, S., Adebajo, A., Barber, R., Beresford, P., Brady, L. M., Brett, J., . . . Williamson, T. (2011). Developing the evidence base of patient and public involvement in health and social care research: the case for measuring impact. *International Journal of Consumer Studies, 35*(6), 628-632. doi:10.1111/j.1470-6431.2011.01020.x

Stergiopoulos, S., Michaels, D. L., Kunz, B. L., & Getz, K. A. (2019). Measuring the Impact of Patient Engagement and Patient Centricity in Clinical Research and Development. *Therapeutic Innovation & Regulatory Science*, 2168479018817517. doi:https://dx.doi.org/10.1177/2168479018817517

Stevens, T., Wilde, D., Hunt, J., & Ahmedzai, S. H. (2003). Overcoming the challenges to consumer involvement in cancer research. *Health Expectations, 6*(1), 81-88.

Stevenson, M., & Taylor, B. J. (2019). Involving individuals with dementia as co-researchers in analysis of findings from a qualitative study. *Dementia, 18*(2), 701-712. doi:https://dx.doi.org/10.1177/1471301217690904

Stewart, M. K., Felix, H. C., Olson, M., Cottoms, N., Bachelder, A., Smith, J., . . . Greene, P. G. (2015). Community Engagement in Health-Related Research: A Case Study of a Community-Linked Research Infrastructure, Jefferson County, Arkansas, 2011-2013. *Preventing Chronic Disease, 12*, E115. doi:https://dx.doi.org/10.5888/pcd12.140564

Stewart, R., & Liabo, K. (2012). Involvement in research without compromising research quality. *Journal of Health Services & Research Policy, 17*(4), 248-251. doi:https://dx.doi.org/10.1258/jhsrp.2012.011086

Stickley, T., Rush, B., Shaw, R., Smith, A., Collier, R., Cook, J., . . . Roberts, S. (2009). Participation In Nurse Education: the PINE project...Participation in Nurse Education. *Journal of Mental Health Training, Education & Practice, 4*(1), 11-18. Retrieved from http://libaccess.mcmaster.ca/login?url=http://search.ebscohost.com/login.aspx?direct=true&db=cin20&AN=103807632&site=ehost-live&scope=site

Straiton, N., McKenzie, A., Bowden, J., Nichol, A., Murphy, R., Snelling, T., . . . Symons, T. (2020). Facing the Ethical Challenges: Consumer Involvement in COVID-19 Pandemic Research. *Journal of Bioethical Inquiry, 17*(4), 743-748. doi:https://dx.doi.org/10.1007/s11673-020-10060-5

Strassle, C. L., & Pearson, S. D. (2020). A proposed framework for patient engagement throughout the broader research enterprise. *Journal of Comparative Effectiveness Research, 9*(6), 387-393. doi:https://dx.doi.org/10.2217/cer-2019-0175

Stuhlfauth, S., Foss, C., & Knutsen, I. R. (2019). Coming from two different worlds—A qualitative, exploratory study of the collaboration between patient representatives and researchers. *Health Expectations, 22*(3), 496-503. doi:10.1111/hex.12875

Susanti, H., James, K., Utomo, B., Keliat, B. A., Lovell, K., Irmansyah, I., . . . Brooks, H. (2020). Exploring the potential use of patient and public involvement to strengthen Indonesian mental health care for people with psychosis: A qualitative exploration of the views of service users and carers. *Health Expectations, 23*(2), 377-387. doi:10.1111/hex.13007

Switzer, S., Chan Carusone, S., Guta, A., & Strike, C. (2019). A Seat at the Table: Designing an Activity-Based Community Advisory Committee With People Living With HIV Who Use Drugs. *Qualitative Health Research, 29*(7), 1029-1042. doi:https://dx.doi.org/10.1177/1049732318812773

Synnot, A. J., Cherry, C. L., Summers, M. P., Stuckey, R., Milne, C. A., Lowe, D. B., & Hill, S. J. (2018). Consumer engagement critical to success in an Australian research project: reflections from those involved. *Australian Journal of Primary Health, 24*(3), 197-203. doi:https://dx.doi.org/10.1071/PY17107

Tanner, D. (2012). Co-research with older people with dementia: experience and reflections. *Journal of Mental Health, 21*(3), 296-306. doi:https://dx.doi.org/10.3109/09638237.2011.651658

Taylor, C., Gill, L., Gibson, A., Byng, R., & Quinn, C. (2018). Engaging "seldom heard" groups in research and intervention development: Offender mental health. *Health Expectations, 21*(6), 1104-1110. doi:https://dx.doi.org/10.1111/hex.12807

Taylor, J., Dekker, S., Jurg, D., Skandsen, J., Grossman, M., Marijnissen, A. K., . . . Investigators, A. P. (2021). Making the patient voice heard in a research consortium: experiences from an EU project (IMI-APPROACH). *Research Involvement & Engagement, 7*(1), 24. doi:https://dx.doi.org/10.1186/s40900-021-00267-0

Taylor, S. (2006). A new approach to empowering older people's forums: identifying barriers to encourage participation. *Practice (09503153), 18*(2), 117-128. Retrieved from http://libaccess.mcmaster.ca/login?url=http://search.ebscohost.com/login.aspx?direct=true&db=cin20&AN=106345883&site=ehost-live&scope=site

Telford, R., Boote, J. D., & Cooper, C. L. (2004). What does it mean to involve consumers successfully in NHS research? A consensus study. *Health Expectations, 7*(3), 209-220.

Telford, R., & Faulkner, A. (2004). Learning about service user involvement in mental health research. *Journal of Mental Health, 13*(6), 549-559. Retrieved from http://libaccess.mcmaster.ca/login?url=http://search.ebscohost.com/login.aspx?direct=true&db=cin20&AN=106606464&site=ehost-live&scope=site

Tempfer, C. B., & Nowak, P. (2011). Consumer participation and organizational development in health care: a systematic review. *Wiener Klinische Wochenschrift, 123*(13-14), 408-414. doi:https://dx.doi.org/10.1007/s00508-011-0008-x

Thompson, A. P., MacDonald, S. E., Wine, E., & Scott, S. D. (2020). An Evaluation of Parents' Experiences of Patient Engagement in Research to Develop a Digital Knowledge Translation Tool: Protocol for a Multi-Method Study. *JMIR Research Protocols, 9*(8), e19108. doi:https://dx.doi.org/10.2196/19108

Thompson, J., Bissell, P., Cooper, C. L., Armitage, C. J., & Barber, R. (2014). Exploring the impact of patient and public involvement in a cancer research setting. *Qualitative Health Research, 24*(1), 46-54. doi:https://dx.doi.org/10.1177/1049732313514482

Thornton, H. (2002). Patient perspectives on involvement in cancer research in the UK. *European Journal of Cancer Care, 11*(3), 205-209.

Thornton, H. (2006). Patients and health professionals working together to improve clinical research: where are we going? *European Journal of Cancer, 42*(15), 2454-2458.

Thornton, H., Edwards, A., & Elwyn, G. (2003). Evolving the multiple roles of 'patients' in health-care research: reflections after involvement in a trial of shared decision-making. *Health Expectations, 6*(3), 189-197.

Tierney, E., McEvoy, R., O'Reilly-de Brun, M., de Brun, T., Okonkwo, E., Rooney, M., . . . MacFarlane, A. (2016). A critical analysis of the implementation of service user involvement in primary care research and health service development using normalization process theory. *Health Expectations, 19*(3), 501-515. doi:https://dx.doi.org/10.1111/hex.12237

Tremblay, M. C., Bradette-Laplante, M., Berube, D., Briere, E., Moisan, N., Niquay, D., . . . Witteman, H. O. (2020). Engaging indigenous patient partners in patient-oriented research: lessons from a one-year initiative. *Research Involvement & Engagement, 6*, 44. doi:https://dx.doi.org/10.1186/s40900-020-00216-3

Trivedi, P., & Wykes, T. (2002). From passive subjects to equal partners: qualitative review of user involvement in research. *British Journal of Psychiatry, 181*, 468-472.

Troya, M. I., Chew‐Graham, C. A., Babatunde, O., Bartlam, B., Higginbottom, A., & Dikomitis, L. (2019). Patient and Public Involvement and Engagement in a doctoral research project exploring self‐harm in older adults. *Health Expectations, 22*(4), 617-631. doi:10.1111/hex.12917

Truman, C., & Raine, P. (2002). Experience and meaning of user involvement: some explorations from a community mental health project. *Health & Social Care in the Community, 10*(3), 136-143.

Tullo, E. S., Robinson, L., & Newton, J. (2015). Comparing the perceptions of academics and members of the public about patient and public involvement in ageing research. *Age and Ageing, 44*(3), 533-536. doi:10.1093/ageing/afu193

Tuttle, K. R., Knight, R., Appelbaum, P. S., Arora, T., Bansal, S., Bebiak, J., . . . Kidney Precision Med, P. (2021). Integrating Patient Priorities with Science by Community Engagement in the Kidney Precision Medicine Project. *Clinical Journal of the American Society of Nephrology, 16*(4), 660-668. doi:10.2215/cjn.10270620

Vale, C. L., Cragg, W. J., Cromarty, B., Hanley, B., South, A., Stephens, R., . . . Gafos, M. (2018). When participants get involved: reconsidering patient and public involvement in clinical trials at the MRC Clinical Trials Unit at UCL. *Trials [Electronic Resource], 19*(1), 95. doi:https://dx.doi.org/10.1186/s13063-018-2471-4

Vale, C. L., Tierney, J. F., Spera, N., Whelan, A., Nightingale, A., & Hanley, B. (2012). Evaluation of patient involvement in a systematic review and meta-analysis of individual patient data in cervical cancer treatment. *Systematic Reviews, 1*, 23. doi:https://dx.doi.org/10.1186/2046-4053-1-23

van der Ham, A. J., van Erp, N., & Broerse, J. E. (2016). Monitoring and evaluation of patient involvement in clinical practice guideline development: lessons from the Multidisciplinary Guideline for Employment and Severe Mental Illness, the Netherlands. *Health Expectations, 19*(2), 471-482. doi:https://dx.doi.org/10.1111/hex.12370

van Draanen, J., Jeyaratnam, J., O'Campo, P., Hwang, S., Harriott, D., Koo, M., & Stergiopoulos, V. (2013). Meaningful inclusion of consumers in research and service delivery. *Psychiatric Rehabilitation Journal, 36*(3), 180-186. doi:https://dx.doi.org/10.1037/prj0000014

van Staa, A., Jedeloo, S., Latour, J. M., & Trappenburg, M. J. (2010). Exciting but exhausting: experiences with participatory research with chronically ill adolescents. *Health Expectations, 13*(1), 95-107. doi:https://dx.doi.org/10.1111/j.1369-7625.2009.00574.x

van Wersch, A., & Eccles, M. (2001). Involvement of consumers in the development of evidence based clinical guidelines: practical experiences from the North of England evidence based guideline development programme. *Quality in Health Care, 10*(1), 10-16.

Vat, L. E., Finlay, T., Robinson, P., Barbareschi, G., Boudes, M., Diaz Ponce, A. M., . . . Schuitmaker-Warnaar, T. J. (2021). Evaluation of patient engagement in medicine development: A multi-stakeholder framework with metrics. *Health Expectations, 24*(2), 491-506. doi:https://dx.doi.org/10.1111/hex.13191

Vat, L. E., Ryan, D., & Etchegary, H. (2017). Recruiting patients as partners in health research: a qualitative descriptive study. *Research Involvement & Engagement, 3*, 15. doi:https://dx.doi.org/10.1186/s40900-017-0067-x

Vat, L. E., Warren, M., Goold, S., Davidge, E. B., Porter, N., Schuitmaker-Warnaar, T. J., . . . Etchegary, H. (2020). Giving patients a voice: a participatory evaluation of patient engagement in Newfoundland and Labrador Health Research. *Research Involvement & Engagement, 6*, 39. doi:https://dx.doi.org/10.1186/s40900-020-00206-5

Vogsen, M., Geneser, S., Rasmussen, M. L., Horder, M., & Hildebrandt, M. G. (2020). Learning from patient involvement in a clinical study analyzing PET/CT in women with advanced breast cancer. *Research Involvement & Engagement, 6*, 1. doi:https://dx.doi.org/10.1186/s40900-019-0174-y

Wale, J. L., & Sullivan, M. (2020). Exploration of the visibility of patient input in final recommendation documentation for three health technology assessment bodies. *International Journal of Technology Assessment in Health Care, 36*(3), 197-203. doi:https://dx.doi.org/10.1017/S0266462320000240

Walker, S. (2018). Involving People Who Self-Harm in Research Design. *Journal of Nursing Research (Lippincott Williams & Wilkins), 26*(1), 68-70. doi:10.1097/jnr.0000000000000208

Wall, D., & Window, S. (2004). NHS support team. Clinical Governance Support Team: patients as a virtue. *Clinical Governance: An International Journal, 9*(1), 67-72. Retrieved from http://libaccess.mcmaster.ca/login?url=http://search.ebscohost.com/login.aspx?direct=true&db=cin20&AN=106781731&site=ehost-live&scope=site

Warren, M., Leamon, T., Hall, A., Twells, L., Street, C., Stordy, A., . . . Etchegary, H. (2020). The Role of Patient Advisory Councils in Health Research: Lessons From Two Provincial Councils in Canada. *Journal of Patient Experience, 7*(6), 898-905. doi:https://dx.doi.org/10.1177/2374373520909598

Warren, N. T., Gaudino, J. A., Jr., Likumahuwa-Ackman, S., Dickerson, K., Robbins, L., Norman, K., . . . DeVoe, J. E. (2018). Building Meaningful Patient Engagement in Research: Case Study From ADVANCE Clinical Data Research Network. *Medical Care, 56 Suppl 10 Suppl 1*, S58-S63. doi:https://dx.doi.org/10.1097/MLR.0000000000000791

Weeks, L., Polisena, J., Scott, A. M., Holtorf, A. P., Staniszewska, S., & Facey, K. (2017). Evaluation of Patient and Public Involvement Initiatives in Health Technology Assessment: A Survey of International Agencies. *International Journal of Technology Assessment in Health Care, 33*(6), 715-723. doi:https://dx.doi.org/10.1017/S0266462317000976

Wennerstrom, A., Springgate, B. F., Jones, F., Meyers, D., Henderson, N., Brown, A., . . . Norris, K. C. (2018). Lessons on Patient and Stakeholder Engagement Strategies for Pipeline to Proposal Awards. *Ethnicity & Disease, 28*(Suppl 2), 303-310. doi:https://dx.doi.org/10.18865/ed.28.S2.303

Westfall, J. M., VanVorst, R. F., Main, D. S., & Herbert, C. (2006). Community-based participatory research in practice-based research networks. *Annals of Family Medicine, 4*(1), 8-14.

Wheeler, S., MacKay, J., Moody, L., D'Souza, J., & Gilbert, J. (2020). Engaging Patient and Family Advisors in Health-Care System Planning: Experiences and Recommendations. *Journal of Patient Experience, 7*(3), 331-337. doi:https://dx.doi.org/10.1177/2374373519840343

Wilson, C., Fothergill, A., & Rees, H. (2010). A potential model for the first all Wales mental health service user and carer-led research group. *Journal of Psychiatric & Mental Health Nursing, 17*(1), 31-38. doi:https://dx.doi.org/10.1111/j.1365-2850.2009.01473.x

Wilson, H., Dashiell-Aje, E., Anatchkova, M., Coyne, K., Hareendran, A., Leidy, N. K., . . . Wyrwich, K. (2018). Beyond study participants: a framework for engaging patients in the selection or development of clinical outcome assessments for evaluating the benefits of treatment in medical product development. *Quality of Life Research, 27*(1), 5-16. doi:https://dx.doi.org/10.1007/s11136-017-1577-6

Young, H. M., Miyamoto, S., Henderson, S., Dharmar, M., Hitchcock, M., Fazio, S., & Tang-Feldman, Y. Meaningful Engagement of Patient Advisors in Research: Towards Mutually Beneficial Relationships. *Western Journal of Nursing Research*, 10. doi:10.1177/0193945920983332

Zibrowski, E., McDonald, S., Thiessen, H., VanDusen, R., Boden, C., Carr, T., . . . Groot, G. (2020). Developing a program theory of patient engagement in patient-oriented research and the impacts on the health care system: protocol for a rapid realist review. *CMAJ open, 8*(3), E530-E534. doi:https://dx.doi.org/10.9778/cmajo.20190181
